# A revision of the *Aleiodes bakeri* (Brues) species subgroup of the *A. seriatus* species group with the descriptions of 18 new species from the Neotropical Region

**DOI:** 10.3897/zookeys.964.56131

**Published:** 2020-08-27

**Authors:** Scott R. Shaw, Eduardo M. Shimbori, Angélica M. Penteado-Dias

**Affiliations:** 1 Department of Ecosystem Science and Management, University of Wyoming, Laramie, Wyoming 82071-3354, USA University of Wyoming Laramie United States of America; 2 ESALQ/USP, Departamento de Entomologia e Acarologia – LEA, Avenida Pádua Dias, 11 Piracicaba/SP, CEP 13418-900, Brazil ESALQ/USP, Departamento de Entomologia e Acarologia – LEA Piracicaba Brazil; 3 Universidade Federal de São Carlos, Rodovia Washington Luiz, km 235, CEP 13 565-905, São Carlos, SP, Brazil Universidade Federal de São Carlos São Carlos Brazil

**Keywords:** Aleiodini, Erebidae, koinobionts, parasitoid wasps, taxonomy

## Abstract

The *Aleiodes bakeri* (Brues) species subgroup of the *A. seriatus* species group is defined based on two previously described species, *A. bakeri* and *A. nigristemmaticum* (Enderlein), and is greatly expanded in this paper with an identification key, descriptions, and illustrations of 18 new species from the Neotropical Region: *A. andinus* Shaw & Shimbori, **sp. nov.**; *angustus* Shimbori & Shaw, **sp. nov.**; *asenjoi* Shimbori & Shaw, **sp. nov.**; *bahiensis* Shimbori & Shaw, **sp. nov.**; *barrosi* Shimbori & Shaw, **sp. nov**.; *brevicarina* Shimbori & Shaw, **sp. nov.**; *coariensis* Shimbori & Shaw, **sp. nov.**; *goiasensis* Shimbori & Shaw, **sp. nov**.; *gonodontivorus* Shaw & Shimbori, **sp. nov.**; *hyalinus* Shimbori & Shaw, **sp. nov.**; *inga* Shimbori & Shaw, **sp. nov.**; *joaquimi* Shimbori & Shaw, **sp. nov.**; *lidiae* Shimbori & Shaw, **sp. nov.**; *mabelae* Shimbori & Shaw, **sp. nov.**; *maculosus* Shimbori & Shaw, **sp. nov.**; *ovatus* Shimbori & Shaw, **sp. nov.**; *santarosensis* Shaw & Shimbori, **sp. nov.**; and *taurus* Shimbori & Penteado-Dias, **sp. nov.** It is hypothesized that the *A. bakeri* species subgroup is a monophyletic lineage within the larger and probably artificial *A. seriatus* species group (those *Aleiodes* with a comb of flat setae at the apex of the hind tibia), and can be distinguished from other members of the *seriatus* group by having the hind wing vein r present, although weakly indicated; the hind wing marginal cell suddenly widened at junction of veins RS and r; the subbasal cell of the fore wing mostly glabrous but often with two rows of short setae subapically; glabrous regions of the wings also commonly found in the first subdiscal, discal, and basal cells of the fore wing, and the basal cell of hind wing; ocelli quite large, with the width of a lateral ocellus being distinctly larger than the ocellar-ocular distance; and being relatively large *Aleiodes* species with body almost entirely brownish yellow or reddish brown. In addition, a new replacement name, *Aleiodes buntikae* Shimbori & Shaw, **nom. nov.**, is proposed for the species formerly called Aleiodes (Hemigyroneuron) bakeri Butcher & Quicke, 2011.

## Introduction

*Aleiodes* Wesmael (Hymenoptera: Braconidae: Rogadinae; tribe Aleiodini) is the most common and species-rich rogadine genus worldwide (S.R. Shaw et al. 1997; Areekul-Butcher and Quicke 2011; Shimbori and S.R. Shaw 2014; van Achterberg et al. 2020). *Aleiodes* species are sometimes commonly called “mummy wasps” (S.R. Shaw 2006) because of their peculiar and distinctive habit of pupating inside the remains of the host caterpillar, which shrinks and dries into a distinctive caterpillar “mummy” (M.R. Shaw 1983, 1994; M.R. Shaw and Huddleston 1991; S.R. Shaw et al.1997; S.R. Shaw 2006; Zaldívar–Riverón et al. 2008; Shimbori and S.R. Shaw 2014). There are at least 212 named *Aleiodes* species described from the New World Region, of which at least 143 occur in the Nearctic Region and 108 in the Neotropical Region (Yu et al. 2012; Garro et al. 2017). The larger number of named species in Nearctic Region is most likely due to more research effort in this region (S.R. Shaw et al. 1997, 1998a, 1998b, 2006, 2013; Marsh and S.R. Shaw 1998, 1999, 2001, 2003; Fortier 2009), rather than any particular biological process (Quicke 2012). However, the number of known *Aleiodes* species from the Neotropical Region has been rising with increasing discovery and focus of studies of the tropical fauna (S.R. Shaw 1993; Townsend and S.R. Shaw 2009; Shimbori and Penteado-Dias 2011; Shimbori and S.R. Shaw 2014; Shimbori et al. 2015; Garro et al. 2017).

Due to the high diversity of species in this genus, revisionary studies of *Aleiodes* have progressed in recent years by defining and examining species groups (S.R. Shaw et al.1997, 1998a, 1998b, 2006, 2013; Marsh and S.R. Shaw 1998, 1999, 2001, 2003; Fortier 2009; Townsend and S.R. Shaw 2009; Shimbori and S.R. Shaw 2014). S.R. Shaw et al. (1997) divided *Aleiodes* into 15 species groups, with two additional groups proposed after additional phylogenetic analyses (Fortier and S.R. Shaw 1999) plus one subgenus (Shimbori et al. 2016). One of these, the *A. seriatus* species group, was the initial focus for this study. Marsh and S.R. Shaw (1998) defined the *A. seriatus* species group as comprising those *Aleiodes* species with a row (or comb) of flattened setae at the apex of the hind tibia on the inner side (Fig. 3). Marsh and S.R. Shaw (1998) circumscribed the *A. seriatus* species group as including *Aleiodes seriatus* (Herrich-Schäffer), eight other named species, and five new species from the Nearctic Region. Subsequently, studies by Townsend and S.R. Shaw, (2009)Shimbori and S.R. Shaw (2014) proposed additional new species of the *A. seriatus* species group, and indicated that the group may be particularly diverse in the neotropics. Phylogenetic research by Fortier (1999) supports the hypothesis that the *A. seriatus* species group is a monophyletic group as defined by Marsh and S.R. Shaw (1998) and Fortier and S.R. Shaw (1999), although some subsequent studies suggest that similarly appearing combs of flat setae may have evolved independently in some other lineages within Aleiodini (Areekul-Butcher and Quicke 2012; van Achterberg and M.R. Shaw 2016). For example, a molecular phylogeny for Thai species of *Aleiodes*, based solely on the DNA barcoding region of the gene COI, recovered at least two separate lineages where the specialized comb of setae is present, one of which is paraphyletic (Areekul-Butcher and Quicke 2012). Additionally, the Neotropical subgenus Athacryvac (Braet & van Achterberg), which is morphologically distinct from any of the species groups previously defined, and is clearly independent from the *A. seriatus* species group, also exhibits a distinct comb of specialized setae on hind tibia reinforcing the homoplasious nature of this character (Shimbori et al. 2016). For the Palearctic fauna, van Achterberg and M.R. Shaw (2016) adopted a different system of division, including overall less species groups when compared with the division based on the Nearctic fauna (S.R. Shaw et al. 1997). Further supported by a molecular phylogeny, also based on DNA barcoding, the system circumscribes six or seven species groups, comprising most of the Palearctic species, but with several species left outside species groups because their relationships are not yet resolved (van Achterberg et al. 2020).

Some confusion could result since a similar comb of flat hind tibial setae has also evolved in some genera of the tribe Rogadini such as *Rogas* Nees, *Triraphis* Ruthe, *Cystomastax* Szepligeti, and *Macrostomion* Szeplegeti. It is therefore important that specimens are carefully identified as belonging to the genus *Aleiodes* first, using identification keys such as those of van Achterberg (1991) or S.R. Shaw (1997), before applying the species-group concepts used within *Aleiodes*. Additional care must be taken when examining specimens for the presence or absence of the comb of flattened setae on the hind tibia, not only because this feature is microscopically small but also because it only occurs on the inner side, and on the hind tibia only (not on the middle tibia). Despite these challenges, the row of flattened setae along the inner margin of the hind tibia has proven to be a consistently valuable characteristic for recognizing members of the *A. seriatus* species group from the Neotropical Region, where the group appears to be quite diverse (but see Braet and van Achterberg (2011) and Shimbori et al. (2016) for a distinction between the *A. seriatus* species group and the subgenus Athacryvac).

**Figures 1, 2.**
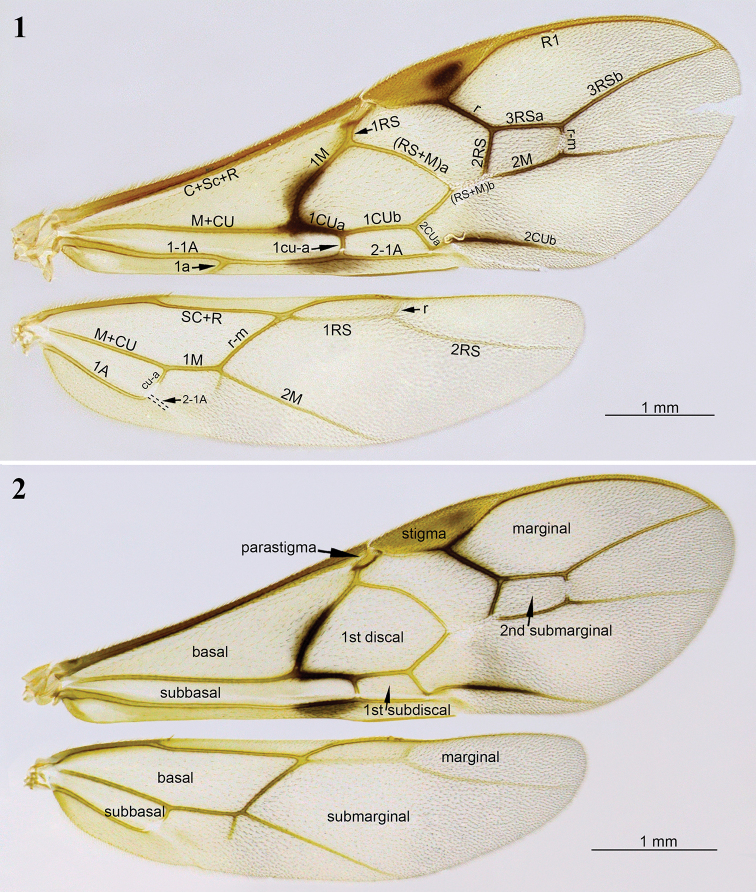
*Aleiodes bakeri* (Brues) species subgroup. **1** Wings with principal veins labelled **2** wings with principal cells labelled.

During our studies we discovered that many of the more commonly encountered specimens of the *A. seriatus* species group from the Neotropical Region fall into a particular presumed lineage characterized by having the hind wing vein r present (as in Figs 1, 23); the marginal cell suddenly widened at junction of veins RS and r (as in Figs 1, 2, 23), the subbasal cell of the fore wing mostly glabrous (as in Figs 2, 27) and usually with two rows of short setae subapically (as in Figs 2, 32), glabrous areas in the first subdiscal, discal, and basal cells of the fore wing (as in Fig. 2) and the basal cell of hind wing (as in Figs 2, 28), ocelli large to enormous (as in Figs 7, 9, 15, 20, 24, 26, 30, 36, 38, 45, 49, 52, 57, 61, 65, 67, 70, 74, 77, 80), with the width of lateral ocellus being distinctly larger than the ocellar-ocular distance (at least 1.8–9.0 × larger), tarsi with well-developed apical spines (as in Fig. 40), and being relatively large specimens with body almost entirely brownish yellow (as in Figs 18, 22, 34, 41, 51, 59, 64, 69, 81) or reddish brown (as in Figs 29, 48, 55, 73). The oldest available name for a species in this distinctive lineage is *Aleiodes bakeri* (Brues), therefore in this paper we propose to call this presumed lineage the *Aleiodes bakeri* (Brues) species subgroup of the *A. seriatus* species group. A technical argument might be made that since “species groups” are informal constructs that merely designate groups of similar or related species, this lineage might be equally well called a “species group” but we prefer the term “subgroup” to remind the reader that this is a cluster of species within a previously named species group (the *A. seriatus* species group).

Although *Aleiodes bakeri* (Brues) was described and named more than a century ago, and is among the commonest of species covered in this manuscript, its identity and relationships to other species have remained largely obscure. A closely related species, *Aleiodes nigristemmaticum* (Enderlein) is the only other previously named species in this subgroup, and the only one to extend its range into the southern parts of the Nearctic Region (Marsh and S.R. Shaw 1998). Otherwise the species subgroup is found exclusively in the neotropics. In this paper, we describe and name 18 other new species of the *Aleiodes bakeri* (Brues) species subgroup of the *A. seriatus* species group.

## Materials and methods

For identification of the braconid subfamily Rogadinae see van Achterberg (1993) or Sharkey (1997). For recognition of rogadine genera refer to the identification keys of van Achterberg (1991) or S.R. Shaw (1997). The definition of *Aleiodes* adopted here follows that of van Achterberg (1991), S.R. Shaw (1993, 1997, 2006), and S.R. Shaw et al. (1997). Species groups within *Aleiodes* have been defined and clarified by S.R. Shaw et al. (1997), Marsh and S.R. Shaw (1998), Fortier and S.R. Shaw (1999), Zaldívar–Riverón et al. 2008, and Townsend and S.R. Shaw, (2009); although for the Western Palearctic fauna van Achterberg and M.R. Shaw (2016) and van Achterberg et al. (2020) divided the genus along different lines.

Morphological terminology for descriptions follows that of Sharkey and Wharton (1997), S.R. Shaw et al. (1997), Shimbori et al. (2015, 2016), and Garro et al. (2017). Microsculpture terminology follows that of Harris (1979). Wing vein terminology follows the system adopted by Sharkey and Wharton (1997) (see Figs 1, 2). The term “inclivous” is applied to describe the orientation of the vein fore wing 2CUa, where the more transverse (= vertical) veins are considered less inclivous. Measurements were taken following Shimbori et al. (2016), except for the pronotal collar length, which refers to the median length of pronotum in dorsal view. We follow Karlsson and Ronquist (2012) in defining the mesosomal area just lateral to the mesoscutellar disc (or scutellum) as the “mesoscutellar trough”. The occipital carina in this group of species (and in *Aleiodes* in general) is either complete (as in Fig. 7) or interrupted mid-dorsally (as in Fig. 15). In some cases, among other species of the *A. seriatus* species group not treated in this paper, the interruption in the occipital carina is accompanied by a deviation of the carina toward the ocelli and/or an indentation on the occiput, therefore the descriptions use the terms “occiput indented”(or not) medially, and “occipital carina curved” (or not) towards the ocelli. Abbreviations used throughout the descriptions are as follows:

**OOL** distance between eye and lateral ocellus

**OD** diameter of lateral ocellus

**POL** distance between lateral ocelli

**T1** metasomal tergite 1

**T2** metasomal tergite 2

**T3** metasomal tergite 3

A number of specimens from Área de Conservación Guanacaste (ACG) in Costa Rica had sequences of the COI DNA barcoding region generated by standard protocols for the ACG barcode inventory, which are described in detail by Smith et al. (2007, 2008). All sequences are deposited in the Barcode of Life Data System (BOLD, http://www.boldsystems.org; Ratnasingham and Hebert 2007), and access codes are provided for each barcoded specimen.

Examined specimens and types are deposited at the following collections:

**CNCI** Canadian National Collection, Ottawa, Canada

**DCBU** Coleção Entomológica do Departamento de Ecologia e Biologia Evolutiva da Universidade Federal de São Carlos, São Carlos, Brazil

**DZUP** Coleção Entomológica Padre Jesus S. Moure, Departamento de Zoologia da Universidade Federal do Paraná, Curitiba, Brazil

**INBIO** Instituto Nacional de Biodiversidad, Santo Domingo de Heredia, Costa Rica

**MCZC** Museum of Comparative Zoology, Harvard University, Cambridge, USA

**MUSM** Colección de Entomologia del Museo de Historia Natural de La Universidad Nacional Mayor de San Marcos, Lima, Peru

**MZUSP** Museu de Zoologia da Universidade de São Paulo, São Paulo, Brazil

**PASW** Polish Academy of Sciences, Warsaw, Poland

**UFES** Coleção de Insetos do Departamento de Ciências Biológicas da Universidade Federal do Espírito Santo, Vitória, Brazil

**UPP** University of Pennsylvania, Philadelphia, USA

**UWIM** University of Wyoming Insect Museum, University of Wyoming, Laramie, USA

## *Aleiodes bakeri* species subgroup of the *seriatus* species group

### Included species

*Aleiodes bakeri* (Brues, 1912); *nigristemmaticum* (Enderlein, 1920); *andinus* Shaw & Shimbori, sp. nov.; *angustus* Shimbori & Shaw, sp. nov.; *asenjoi* Shimbori & Shaw, sp. nov.; *bahiensis* Shimbori & Shaw, sp. nov.; *barrosi* Shimbori & Shaw, sp. nov.; *brevicarina* Shimbori & Shaw, sp. nov.; *coariensis* Shimbori & Shaw, sp. nov.; *goiasensis* Shimbori & Shaw, sp. nov.; *gonodontivorus* Shaw & Shimbori, sp. nov.; *hyalinus* Shimbori & Shaw, sp. nov.; *inga* Shimbori & Shaw, sp. nov.; *joaquimi* Shimbori & Shaw, sp. nov.; *lidiae* Shimbori & Shaw, sp. nov.; *mabelae* Shimbori & Shaw, sp. nov.; *maculosus* Shimbori & Shaw, sp. nov.; *ovatus* Shimbori & Shaw, sp. nov.; *santarosensis* Shaw & Shimbori, sp. nov.; and *taurus* Shimbori & Penteado-Dias, sp. nov.

### Species subgroup diagnosis

Hind tibia with row of flattened setae along inner margin (as in Fig. 3). Hind wing vein r present, usually weakly indicated (Figs 1, 23); marginal cell suddenly widened at junction of veins RS and r (Figs 1, 2, 23). Subbasal cell of fore wing mostly glabrous (Figs 2, 27, 47, 50, 54, 63, 75), usually with two rows of short setae subapically (Figs 2, 32). Glabrous regions on the wings are also commonly found in the first subdiscal, discal and basal cells of the fore wing (Fig. 2) and the basal cell of hind wing (Figs 2, 28). Ocelli large to enormous (Figs 7, 9, 15, 20, 24, 27, 30, 36, 38, 45, 49, 52, 57, 61, 65, 67, 70, 77, 80), with the width of lateral ocellus being distinctly larger than the ocellar-ocular distance (at least 1.8–9.0 × larger). Tarsi with well-developed apical spines (as in Fig. 40). Relatively large specimens with body almost entirely brownish yellow (as in Figs 18, 22, 34, 41, 51, 59, 64, 69, 81) or reddish brown (as in Figs 29, 48, 55, 73).

**Figure 3.**
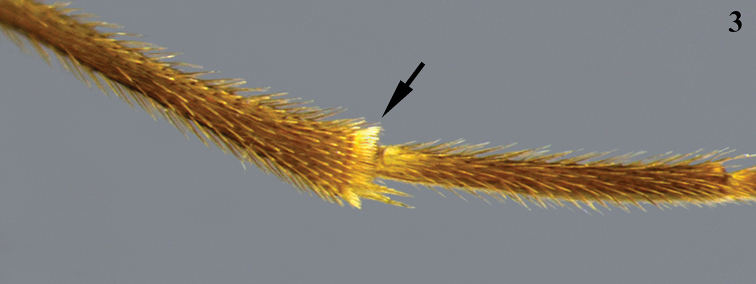
Inner side of hind tibia showing the comb of specialized setae apically (arrow) – *Aleiodes* sp.

### Distribution

Known only from the New World with most species occurring in the Neotropical parts of South America and Central America. The northern limits of the group are set by a few species that occur in Mexico, parts of the Caribbean, and southern Florida. Species of this group have been recorded from the following countries: Bolivia, Brazil, Colombia, Costa Rica, Cuba, Dominican Republic, Ecuador, Honduras, Mexico, Panama Peru, southeastern USA (Florida), Suriname, and Venezuela.

### Biology

As far as known, species of this group are koinobiont endoparasitoids of Noctuoidea caterpillars, with all confirmed hosts of three of the treated species being from the family Erebidae (subfamilies Calpinae, Eulepidotinae and Erebinae). They have been reared from mummified caterpillars of several erebid species including hosts of economical relevance (i.e., *Mocis latipes* (Guenée), an occasional pest of pasture).

### Comments

We propose that the presence of the vein r on hind wing is a putative synapomorphy of this monophyletic group of species within the larger *seriatus* species group.

## Results

### Key to species of the *Aleiodes bakeri* species subgroup

**Table:** 

1	Occipital carina interrupted mid-dorsally (as in Figs 24, 65); subbasal cell of fore wing usually with sparse setae basally (as in Figs 2, 23)	**2**
–	Occipital carina complete dorsally (as in Figs 7, 36, 57); subbasal cell of fore wing rarely with setae basally (as in Figs 32, 75)	**5**
2	Fore wing vein 1a absent (as in Figs 2, 23); hind femur honey yellow to orange-brown (as in Fig. 22)	**3**
–	Fore wing vein 1a present and tubular (as in Figs 1, 12); hind femur orange-brown with infuscate apex (as in Figs 8, 14)	**4**
3	Fore wing second submarginal cell comparatively short (Figs 2, 23), vein 3RSa approx. as long as vein r; flagellum entirely the same color, varying from yellow to light brownish orange (Figs 21, 22)	***A. bakeri* Brues**
–	Fore wing second submarginal cell longer (Fig. 64), vein 3RSa more than 2.0 × longer than vein r; flagellum black at base, gradually lightening toward yellow apex (Fig. 64)	***A. mabelae* sp. nov.**
4	Basal polished triangular area of T1 long, distinctly extending over dorsal surface (Fig. 7). Females with large ovipositor, sheaths 1.4 × longer than hind basitarsus (Figs 8, 11); division of T2 and 3 weak, T3 weakly granulate and without longitudinal carina (Fig. 10)	***A. angustus* sp. nov.**
–	Basal triangular area of T1 short, not extending dorsally (Fig. 16). Females with ovipositor sheaths 0.5–0.7 × longer than hind basitarsus (Figs 14, 17); T2 and T3 divided by deep and crenulate sulcus, T3 striate and with longitudinal carina on basal 0.75 (Fig. 16)	***A. asenjoi* sp. nov.**
5	Hind tibia whitish yellow basally and dark brown or black apically, fore and mid tibia basally or entirely whitish yellow; all tarsomeres 1–4 whitish yellow (Figs 29, 55, 77); body reddish brown or brownish orange (Figs 29, 55–57, 73)	**6**
–	Hind tibia and tarsi usually entirely brownish yellow, if tibia basally and tarsomeres 1–4 whitish yellow, then apex of hind tibia not dark brown and body brownish yellow (Fig. 25)	**9**
6	Thorax mottled light pale yellow and brown (Fig. 66), mesoscutum pale yellow contrasting with brown tegula and scutellum (Fig. 67)	***A. maculosus* sp. nov.**
–	Thorax entirely dark reddish brown (Fig. 55) or brownish orange; mesoscutum, scutellum and tegula of similar dark color (Figs 57, 74)	**7**
7	Fore wing with distinct, rounded infuscate spot around veins 1M and 1CUa (Fig. 75); head light yellow, but brown at lower face and dark brown at vertex and around occipital carina (Fig. 74); palpi dark brown (Fig. 74)	***A. ovatus* sp. nov.**
–	Fore wing without distinct infuscate spot but basal half of vein 1M infuscate (Fig. 32); head entirely dark reddish brown to yellowish brown, including palpi (Fig. 29)	**8**
8	Fore wing first discal cell evenly, rather densely setose (Fig. 32); basal cell mostly setose but less densely than first discal cell (Fig. 32). Hind tibia and femur dark brown apically (Fig. 29). Fore wing with distinct infuscate area present at basal half of vein 1M (Fig. 32). Hind wing vein 2-1A absent (Fig. 31)	***A. brevicarina* sp. nov.**
–	Fore wing discal cell with distinct glabrous spot along veins 1M and 1CU; basal cell mostly glabrous, setose below costal vein and anteriorly near vein 1M. Fore wing without infuscate spots (Fig. 55). Hind wing vein 2-1A present, although short (Fig. 58)	***A. joaquimi* sp. nov.**
9	First subdiscal cell of fore wing widening apically and relatively long (Fig. 28); vein 1CUb ~ 1.7–2.2 × longer than 1CUa. Vein 2CUa strongly inclivous, vein 1CUa 0.9–1.2 × longer than 2CUa (Figs 27–28)	***A. barrosi* sp. nov.**
–	First subdiscal cell of fore wing not widening apically and shorter; vein 1CUb 1.00–1.25 × longer than 1CUa. Vein 2CUa less inclivous, vein 1CUa 1.5–2.0 × longer than vein 2CUa	**10**
10	Antenna entirely yellowish (as in Fig. 18)	**11**
–	Antenna dark brown basally, apically light brown to yellow (as in Figs 33, 34); rarely flagellum mostly yellowish with few basal segments slightly darker, but at least pedicel dark brown and scape with lateral brown patch	**13**
11	All wing veins evenly brown, membrane hyaline without distinct infuscate patches around veins (Fig. 51)	***A. hyalinus* sp. nov.**
–	Veins 1M, 1CUa, and part of 2CUb dark brown, darker than remaining veins, membrane around these veins, and below vein 1-1A apically, at least weakly infuscate (as in Figs 19, 78)	**12**
12	All legs with tarsomeres 1–4 and at least base of tibiae whitish yellow, contrasting with brownish orange femur (Fig. 76)	***A. santarosensis* sp. nov.**
–	Legs honey yellow (Fig. 18)	***A. bahiensis* sp. nov.**
13	All femora dark brown apically (Fig. 34). Stigma mostly dark brown, yellow at basal and apical tips (Fig. 35)	***A. coariensis* sp. nov.**
–	Fore and mid femora yellow, hind femur sometimes mostly dark brown. Stigma mostly or entirely yellow (Fig. 4)	**14**
14	Flagellum with two colors, black basally and yellow apically, not gradually lighter, usually with one “transitional” flagellomere, lighter than basal and darker than apical flagellomeres (Fig. 4). Ovipositor sheaths variable, most species with rounded apex and apical point (Figs 46, 62, 82) but some with sheaths truncated apically, without point	**15**
–	Flagellum gradually lightening toward apex. Ovipositor sheaths truncated apically, without point (as in Fig. 40)	**18**
15	Basal cell of fore wing evenly, rather densely setose, without large glabrous spots (Fig. 6). Ovipositor sheaths truncated apically, without point (Fig. 5)	***A. andinus* sp. nov.**
–	Basal cell of fore wing largely glabrous (Fig. 59), with few sparse setae. Ovipositor sheaths with distinct point apically (Figs 62, 82)	**16**
16	Frons entirely rugulose (Fig. 80); second submarginal cell long and rectangular (Fig. 83), vein 3RSa 2.1 × longer than 2RS; median carina of propodeum defined at basal 0.3, effaced in posterior 0.7 (Fig. 84)	***A. taurus* sp. nov.**
–	Frons shiny coriaceous (Figs 60, 61), sometimes with longitudinal rugae medially; second submarginal cell shorter and trapezoidal (Fig. 59), vein 3RSa ~ 1.4–1.7 × longer than 2RS; median carina of propodeum complete or nearly so	**17**
17	Hind femur mostly dark brown (Fig. 59); dark markings around veins 1M/1CUa and vein r conspicuous (Fig. 63), veins dark brown and wing membrane clearly infuscate around veins. Diameter of lateral ocellus 2.4–2.5 × distance between ocelli (Fig. 61). Scape shorter, 1.7–1.9 × longer than pedicel (Fig. 60)	***A. lidiae* sp. nov.**
–	Hind femur brownish yellow (Fig. 41); veins 1M/1CUa and vein r faintly darker, membrane around veins not distinctly infuscate (Figs 44, 47). Diameter of lateral ocellus 2.9–3.8 × distance between ocelli (Fig. 45). Scape longer, 2.5–2.6 × longer than pedicel (Figs 42–43)	***A. gonodontivorus* sp. nov.**
18	Second submarginal cell of fore wing long, vein 3RSa ~ 2.0 × longer than 2RS (Fig. 54). Frons without distinct lateral carina (Fig. 52)	***A. inga* sp. nov.**
–	Second submarginal cell short, vein 3RSa 1.3–1.5 × longer than vein 2RS (Figs 39, 71). Frons with distinct lateral carina (Figs 38, 70)	**19**
19	Occipital carina in dorsal view distinctly curved medially, carina weaker mid-dorsally (Fig. 38). Vertex rugose-granular (Fig. 38). Fore wing vein r ~ 1.5 × longer than vein 2RS (Fig. 39)	***A. goiasensis* sp. nov.**
–	Occipital carina straight or weakly bent mid-dorsally (Fig. 70). Vertex weakly granular-coriaceous (Fig. 70). Fore wing vein r 1.0–1.1 × longer than vein 2RS (Fig. 71)	***A. nigristemmaticum* Enderlein**

### Species descriptions

#### Aleiodes andinus

Taxon classificationAnimaliaHymenopteraBraconidae

Shaw & Shimbori sp. nov.

8A698044-D054-591A-A410-559BA1970759

http://zoobank.org/095E7E57-B313-4549-BFC8-97F655CD97AA

Figs 4–7

##### Type material.

Holotype, female (MUSM) “PERU: CUSCO, La Convención, Echarate, C. Segakiato. 11°45'38.6"S, 73°14'57.7"W 908m. 01.ii.2011. M. Alvarado & E Rázuri.”

##### Description.

Body length 8.1 mm. Fore wing length 6.4 mm.

***Head.*** In dorsal view eye length/temple 4.0. Eye height/head width 0.4. Eye height/minimum distance between eyes 1.1. OD/POL 2.2. OD/OOL 2.4. Frons excavated. Frons lateral carina present. Occipital carina dorsally complete, weakly curved. Occiput in dorsal view nearly straight, not indented medially. Occipital carina ventrally meeting hypostomal carina. Mid-longitudinal crest at upper face present. Hypoclypeal depression/face width 0.33. Malar space/eye height 0.19. Face height/width 0.7. Clypeus height/width 0.66. Clypeus convex, granulate. Sculpture of head mostly shiny granulate. Face weakly rugose, transversely rugose-striate around median crest.

***Antenna.*** Antennal segments 55. Antenna/body length 1.2. Scape/pedicel length 2.3. Length of first/second flagellomere 1.2. Fourth flagellomere length/apical width 1.7. Tip of apical segment of antenna pointed.

***Mesosoma.*** Length/height ~ 1.6. Width of mesoscutum/width of head 0.7. Mesoscutum length/width ~ 1.1. Pronotal collar/vertex 0.7. Prescutellar sulcus with complete median carina, rugose laterally without distinct lateral carinae. Mesoscutum posterior border with distinct complete carina. Metanotum with mid-longitudinal carina complete, connecting to a carinate pit posteriorly, carina bisecting posterior pit, although weaker posteriorly. Metanotum mid-pit present, delimited by carinae. Mid-longitudinal carina of propodeum present at basal 0.7, absent posteriorly. Ventral mid-line of mesopleuron set within shallow smooth sulcus; pit at ventral mid-line absent. Notauli weakly indicated anteriorly, indistinctly crenulate. Sternaulus weakly indicated anteriorly, rugose. Sculpture of mesosoma mostly granulate. Pronotum rugose laterally, or granulate ventrally, pronotal groove crenulate anteriorly, short subventral longitudinal carina present. Mesopleuron rugose below subalar groove. Subalar groove crenulate. Mid-posterior region of mesoscutum rugose. Mesoscutellar trough entirely costate. Metanotum mostly smooth and weakly crenulate. Propodeum mostly rugose.

***Wings.*** Fore wing: Stigma length/height 3.4. Vein r/2RS 1.3. Vein r/RS+Mb 1.2. Vein 3RSa/2RS 1.8. Vein 3RSa/2M 0.83. Vein 3RSa/3RSb 0.46. Vein 1CUa/1CUb 1.0. Vein 1CUa/2CUa 2.1. Vein 1cu-a weakly inclivous. Vein 1M nearly straight. Vein RS+Ma virtually straight. Vein M+CU weakly sinuate. Vein 1-1A very weakly sinuate apically. Vein 1a absent. Second submarginal cell trapezoidal. Subbasal cell glabrous, with two parallel rows of short setae subapically, a row of setae just below of vein 1CUa and M+CU apically, a row of setae apically just above vein 1-1A, and sparsely setose at base. Basal cell evenly setose. Hind wing: Vein RS bent at basal 0.3, with vein r. Marginal cell narrowest at base. Vein M+CU/1M 1.6. Vein M+CU/r-m 1.3. Vein m-cu present, spectral. Vein m-cu position relative to vein r-m antefurcal. Vein 2-1A absent. Basal cell sparsely setose, bare posteriorly.

***Hind legs.*** Femur length/width 5.0. Length of tibia/tarsi ~ 0.9. Length of basitarsus/tarsi 2–4 ~ 0.7. Sculpture of hind coxa dorsally mostly shiny granular-coriaceous, finely striate apically. Tarsal claws not pectinate.

***Metasoma.***T1 length/apical width ~ 1.3. T2 length/apical width ~ 0.9. T3 length/apical width 0.7. Mid-longitudinal carina extending until near apex of T3. Metasoma sculpture T1 rugose, T2 and most of T3 striate-rugose, remainder metasoma smooth. Ovipositor sheath/hind basitarsus 0.5. Ovipositor sheaths narrow, with truncate apex; apical point absent.

***Color.*** Brownish yellow. Antenna with basal 12–13 flagellomeres black, apical segments yellow. Wings tinged yellow; stigma and most veins yellow; vein 1M at basal 0.7 and vein 1CUa black, veins r, 2RS, and apex of and 2CUb brown; distinctly infuscate area around base of vein 1M and vein 1CUa, faintly infuscate spots below apex of vein 1-1A and around vein 2CUb.

**Male.** Unknown.

##### Diagnosis.

**Figures 4–7.**
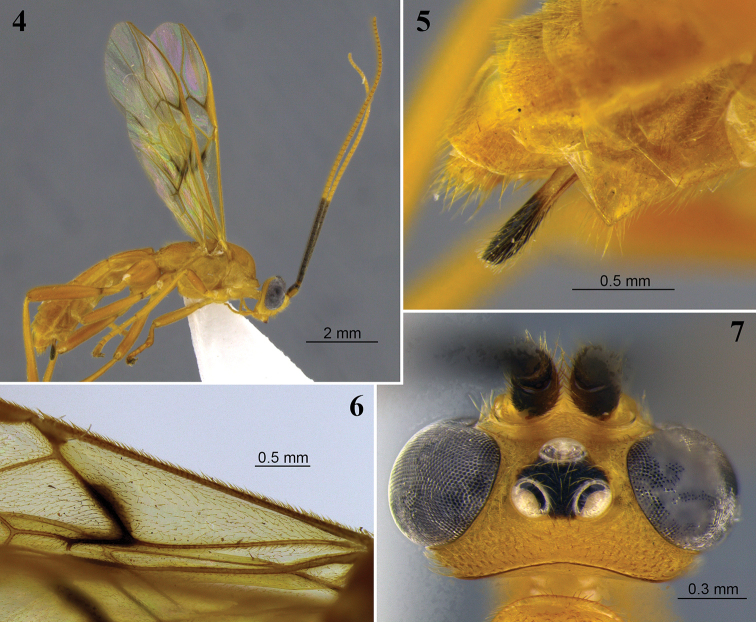
*Aleiodes andinus* sp. nov. **4** lateral habitus **5** apex of metasoma and ovipositor, lateral view **6** basal cell of fore wing showing dense setae **7** head, dorsal view.

*Aleiodes andinus* is similar to three other new species described in this paper, *A. gonodontivorus*, *A. lidiae*, and *A. taurus*, which also have a distinctly bicolored flagellum with rapid transition from dark to light color (Figs 4, 41, 59, 79). However, those three species have a fore wing basal cell that is largely glabrous (Figs 47, 63, 83) and ovipositor sheath with an apical point (Figs 46, 62, 82), whereas the basal cell of *A. andinus* is evenly setose (Fig. 6) and the the ovipositor sheath lacks an apical point (Fig. 5).

##### Distribution.

Known only from the type-locality in Cusco, Peru.

##### Etymology.

The name refers to the Andes Mountains, which are prominent features of the Cusco region of Peru where the holotype specimen was collected.

#### Aleiodes angustus

Taxon classificationAnimaliaHymenopteraBraconidae

Shimbori & Shaw sp. nov.

1EDA94BB-C1B3-5F52-BC5B-F7903BD6220A

http://zoobank.org/D0B5D8EA-E4B0-4795-BE9D-7E16376A895C

Figs 8–13

##### Type material.

Holotype, female (CNCI), top label: “Avispas, 400m. PERU Madre de Dios Dept. Sept. 12–20, 1962 L.E. Pena.”, bottom label: “divided Radiellan + Interanal New Genus [hand written] Det. W.R.M. Mason 75.”

Paratypes. 1 female, 1 male (CNCI), same as holotype; 1 male (CNCI) “BRAZIL: Mato Grosso Sinop, X.1974, 350m 12°31'S, 55°37'W malaise, M. Alvarenga”; 1 female (MUSM) “PERU: CUSCO, La Convención, Echarate, C.C. Timpia. 72°49'34.56"/ 12°06'47.04" 519m. 20–21.x.2009. Light. M. Alvarado & Rázuri”; 1 female (MUSM), same data except “... C.C. Pomareni. 72°50'8.89"/ 12°15'28.38" 477m. 08.xi.2009 Light C. Carranza y C. Rossi”; 5 females (MUSM) “PERU: MD, Rio Los Amigos, CICRA, Aeródromo, 276m, 12°33'36"S, 70°06'17.5"W 22–28.vii.2006, Light trap, A. Asenjo”; 3 females (MUSM) “PERU: JU, Pachitea River-System Stat. Panguana am. Rio Llullapichis, trop. Tiefland-Regenwald. 260m, 9°37'S, 74°56'W 2–20.x.2009, G. Riedel.”

##### Description.

Body length 7.3–8.0 mm. Fore wing length 5.9–6.3 mm.

***Head.*** In dorsal view eye length/temple 3.5–4.0. Eye height/head width 0.41–0.43. Eye height/minimum distance between eyes 1.1–1.2. OD/POL 2.4–2.6. OD/OOL 2.5–3.2. Frons excavated. Frons lateral carina weakly indicated. Occipital carina dorsally incomplete. Occiput in dorsal view weakly indented medially. Occipital carina not curved toward ocelli. Occipital carina ventrally meeting hypostomal carina. Mid-longitudinal crest at upper face present. Hypoclypeal depression/face width 0.35–0.45. Malar space/eye height 0.2. Face height/width 0.6–0.7. Clypeus height/width 0.56–0.60. Clypeus convex, granulate. Sculpture of head shiny granular-coriaceous. Face transversely rugose-striate, medially granular-coriaceous below crest.

***Antenna.*** Antennal segments 47–48. Antenna/body length 0.94–0.96. Scape/pedicel length 2.0–2.1. Length of first/second flagellomere 1.2–1.3. Fourth flagellomere length/apical width 1.3–1.4. Tip of apical segment of antenna pointed.

***Mesosoma.*** Length/height 1.5–1.6. Width of mesoscutum/width of head 0.76–0.83. Mesoscutum length/width ~ 1.1. Pronotal collar/vertex 0.6–0.8. Prescutellar sulcus with complete mid-longitudinal carina, and a few irregular and incomplete carinae laterally. Mesoscutum posterior border with distinct complete carina. Metanotum with mid-longitudinal carina present anteriorly. Metanotum mid-pit present, delimited by carinae. Mid-longitudinal carina of propodeum complete. Ventral mid-line of mesopleuron smooth, without distinct sulcus; pit at ventral mid-line present, shallow. Notauli weakly indicated anteriorly, indistinctly crenulate. Sternaulus weakly indicated anteriorly, rugose. Sculpture of mesosoma mostly granulate. Pronotum rugose laterally, pronotal groove curvedly crenulate anteriorly. Mesopleuron rugose below subalar groove. Subalar groove crenulate. Mid-posterior region of mesoscutum rugose. Mesoscutellar trough entirely costate. Metanotum mostly smooth, with one or two pairs of lateral carinae. Propodeum mostly shiny granular-coriaceous, with a few carinae radiating from mid-posterior knob.

***Wings.*** Fore wing: Stigma length/height 3.0–3.2. Vein r/2RS 1.1–1.3. Vein r/RS+Mb 1.4–1.6. Vein 3RSa/2RS 1.2–1.5. Vein 3RSa/2M 0.8–0.9. Vein 3RSa/3RSb 0.32–0.43. Vein 1CUa/1CUb ~ 0.8. Vein 1CUa/2CUa 1.65–1.75. Vein 1cu-a vertical. Vein 1M strongly curved at base. Vein RS+Ma weakly curved. Vein M+CU virtually straight. Vein 1-1A distinctly sinuate basally. Vein 1a present and tubular. Second submarginal cell trapezoidal. Subbasal cell mostly glabrous, with sparse setae basally, a small setose patch at the infuscate region bellow vein 1CUa, and two or three irregular rows of short setae subapically above vein 1-1A. Basal cell with more or less large glabrous region posteriorly, sometimes with sparse setae; costal and apical regions evenly setose. Hind wing: Vein RS bent at basal 0.3, with vein r present. Marginal cell narrowest at base. Vein M+CU/1M 2.3–2.5. Vein M+CU/r-m 1.7–1.8. Vein m-cu present, spectral. Vein m-cu position relative to vein r-m interstitial, or just postfurcal. Vein 2-1A absent. Basal cell sparsely setose, bare posteriorly.

***Hind legs.*** Femur length/width 3.7–4.0. Length of tibia/tarsi 1.2–1.3. Length of basitarsus/tarsi 2–4 ~ 0.7. Sculpture of hind coxa dorsally mostly shiny granular-coriaceous, finely striate apically. Tarsal claws not pectinate.

***Metasoma.***T1 length/apical width ~ 1.0. T2 length/apical width 0.8–0.9. T3 length/apical width 0.7. Mid-longitudinal carina extending until T2. Metasoma sculpture T1 and T2 costate, basal 0.2 of T3 finely costate, remainder terga granular-coriaceous. Ovipositor sheath/hind basitarsus 1.4. Ovipositor sheaths unusually long and with, with truncate apex; apical point absent

***Color.*** Brownish yellow. Hind femur dark brown at apical 0.2; all fifth tarsomeres light brown. Wings faintly tinged yellow; most veins yellow, infuscate spots at fore wing veins 1M/1CUa, apex of 1CUa, 2CUb, and veins enclosing second submarginal cell. Ovipositor sheaths honey brown with dark brown apex.

**Male.** Essentially as in female, but metasoma not laterally compressed apically. Body length 5.6–6.2 mm, fore wing length 4.2–5.4 mm; 42–44 antennomeres.

##### Diagnosis.

**Figures 8–13.**
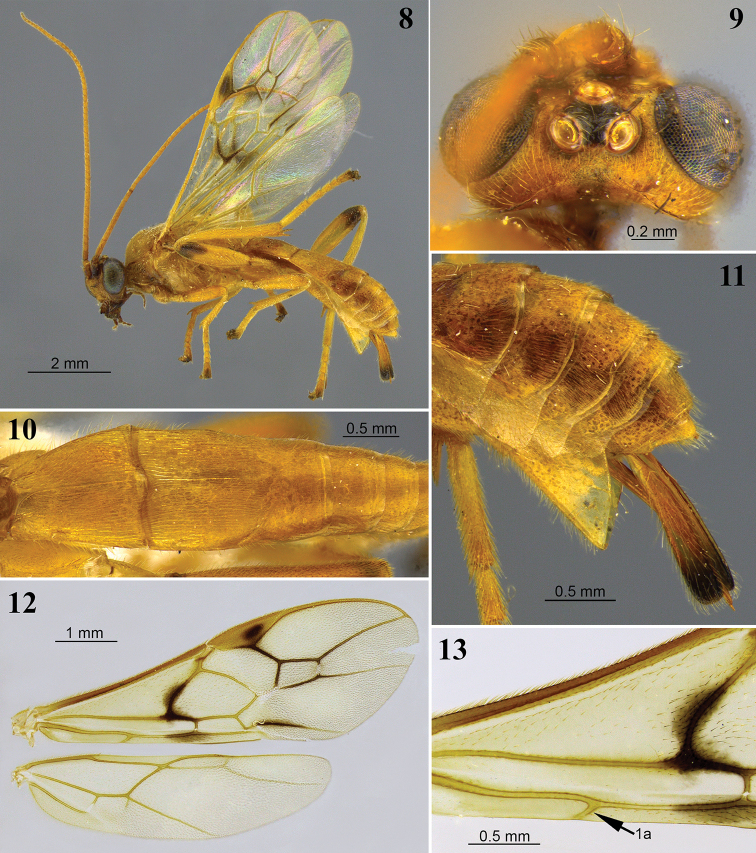
*Aleiodes angustus* sp. nov. **8** lateral habitus **9** head, dorsal view **10** metasoma, dorsal view **11** apex of metasoma and ovipositor sheaths, lateral view **12** wings **13** fore wing basally showing tubular and distinct vein 1a.

**Figures 14–17.**
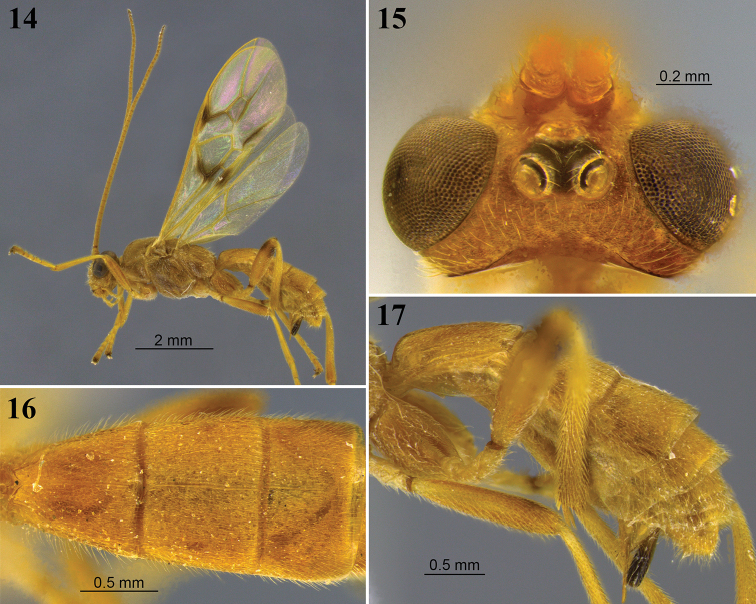
*Aleiodes asenjoi* sp. nov. **14** lateral habitus **15** head, dorsal view **16** metasoma, dorsal view **17** metasomal, lateral view.

*Aleiodes angustus* is the only species in this study with long and wide ovipositor sheaths that are distinctly longer than hind basitarsus (Fig. 11). It is most similar to *A. asenjoi* but has the ovipositor sheaths very long and large (Figs 8, 11); the division between T2/T3 is very weak and T3 is mostly smooth and without a longitudinal carina (Fig. 10); the metasoma is compressed laterally beyond T2 (Figs 10, 11); and the scutellum is entirely yellow. By contrast, in *A. asenjoi* the ovipositor sheaths are much shorter (Figs 14, 17); a division between T2/T3 is present and distinct (Fig. 16); and the scutellum is usually dark brown apically. Males are more difficult to separate; however, males of *A. angustus* have a longer basal triangular polished area that clearly extends dorsally, as compared with strictly basally in *A. asenjoi*.

##### Distribution.

Known from several localities in Peru, and in Mato Grosso state in Brazil.

##### Etymology.

The name *angustus* is from the Latin word for narrow or slender, being a reference to the compressed and narrow apex of the metasoma in this species (Fig. 10).

#### Aleiodes asenjoi

Taxon classificationAnimaliaHymenopteraBraconidae

Shimbori & Shaw sp. nov.

C0925D73-CC54-56E2-96B6-55CE0D846C29

http://zoobank.org/382E1219-318C-43D0-80F9-80AA4D6F3AC4

Figs 14–17

##### Type material.

Holotype, female (MUSM) “PERU: MD, Rio Los Amigos, CICRA, Aeródromo, 276m, 12°33'36"S, 70°06'17.5"W 22–28.vii.2006, Light trap, A. Asenjo.”

Paratypes. 2 females (MUSM), same as holotype; 1 female, 1 male (CNCI) “Avispas, 400m. PERU Madre de Dios Dept. Sept. 12–20, 1962 L.E. Pena”; 3 females (CNCI) “BRAZIL: Bahia, Encruzilhada, XI.1972, M. Alvarenga”; 1 male (DCBU 29634) “Piracuruca, PI, Brasil Parque Nacional Sete Cidades Adm. – Cerrado/Caatinga 04°06'03"S, 41°41'32"W Armadilha Luminosa 22.III.2013 A.S. Soares & E.M. Shimbori cols.”

##### Description.

Body length 5.4–6.2 mm. Fore wing length 4.9–5.4 mm.

***Head.*** In dorsal view eye length/temple 4.1–5.3. Eye height/head width 0.43–0.45. Eye height/minimum distance between eyes 1.1–1.2. OD/POL 1.8–2.5. OD/OOL 1.8–2.5. Frons excavated. Frons lateral carina present in addition to W-shaped carina. Occipital carina dorsally incomplete. Occiput in dorsal view weakly indented medially. Occipital carina not curved toward ocelli. Occipital carina ventrally meeting hypostomal carina. Mid-longitudinal crest at upper face present. Hypoclypeal depression/face width 0.36–0.42. Malar space/eye height 0.18–0.20. Face height/width 0.6–0.7. Clypeus height/width 0.5–0.6. Clypeus convex, strongly bulging, granulate. Sculpture of head shiny granular-coriaceous. Face transversely rugose-striate, medially granular-coriaceous below crest.

***Antenna.*** Antennal segments 45. Antenna/body length 1.0–1.1. Scape/pedicel length 2.0–2.1. Length of first/second flagellomere 1.0–1.1. Fourth flagellomere length/apical width 1.7–1.8. Tip of apical segment of antenna pointed.

***Mesosoma.*** Length/height 1.6–1.7. Width of mesoscutum/width of head 0.71–0.76. Mesoscutum length/width 1.0–1.1. Pronotal collar/vertex 0.6. Prescutellar sulcus with complete mid-longitudinal carina, and 2–4 pairs of rather incomplete carinae laterally. Mesoscutum posterior border with distinct complete carina. Metanotum with mid-longitudinal carina present anteriorly, and with carinate pit mid-posteriorly. Metanotum mid-pit present, delimited by carinae. Mid-longitudinal carina of propodeum complete, or nearly complete. Ventral mid-line of mesopleuron set within shallow smooth sulcus; pit at ventral mid-line absent. Notauli present anteriorly and indistinctly crenulate. Sternaulus absent. Sculpture of mesosoma mostly granulate. Pronotum granulate-rugose laterally, pronotal groove crenulate anteriorly, crenulation curved posteriorly into ventral curved striation. Mesopleuron rugose below subalar groove. Subalar groove sparsely crenulate. Mid-posterior region of mesoscutum rugose. Mesoscutellar trough entirely costate. Metanotum costate. Propodeum mostly rugose.

***Wings.*** Fore wing: Stigma length/height 2.8–3.0. Vein r/2RS 1.1–1.3. Vein r/RS+Mb 1.4–1.7. Vein 3RSa/2RS 1.2–1.5. Vein 3RSa/2M 0.8–0.9. Vein 3RSa/3RSb 0.34–0.42. Vein 1CUa/1CUb 0.75–0.95. Vein 1CUa/2CUa 1.6–1.8. Vein 1cu-a vertical. Vein 1M weakly curved basally. Vein RS+Ma weakly sinuate. Vein M+CU virtually straight. Vein 1-1A distinctly sinuate basally. Vein 1a present and tubular. Second submarginal cell trapezoidal. Subbasal cell mostly glabrous, with sparse setae basally, a small setose patch at the infuscate region bellow vein 1CUa, and two or three irregular rows of short setae subapically above vein 1-1A. Basal cell with more or less large glabrous region posteriorly, sometimes with sparse setae; costal and apical regions evenly setose. Hind wing: Vein RS Bent at basal 0.3, with vein r present. Marginal cell narrowest at base. Vein M+CU/1M 1.8–2.1. Vein M+CU/r-m 1.5–1.7. Vein m-cu present, spectral. Vein m-cu position relative to vein r-m interstitial. Vein 2-1A absent. Basal cell sparsely setose, bare posteriorly.

***Hind legs.*** Femur length/width 4.0–4.3. Length of tibia/tarsi 1.1–1.2. Length of basitarsus/tarsi 2–4 0.73–0.77. Sculpture of hind coxa dorsally mostly shiny granular-coriaceous, finely striate apically. Tarsal claws not pectinate.

***Metasoma.***T1 length/apical width 1.0–1.1. T2 length/apical width 0.75–0.80. T3 length/apical width 0.6–0.7. Mid-longitudinal carina extending until basal 0.5 of T3. Metasoma sculpture T1, T2 and basal 0.7 of T3 rugose-costate, sculpture weaker at T3, or remainder terga granular-coriaceous. Ovipositor sheath/hind basitarsus 0.5–0.7. Ovipositor sheaths relatively narrow, with roughly rounded apex; apical point absent.

***Color.*** Brownish yellow to light brown, including antenna. With more or less distinct brown spots at apex of hind femur and apex of scutellum, sometimes also at apex of mid femur. Wings moderately tinged yellow, vein yellow with typical darker regions on vein 1M, 1CUa and apex of 1-1A and at vein r, 2RS and 2CUb, stigma with a round brown spot mid-apically. Ovipositor sheaths dark brown.

**Male.** Essentially as in female, but fore wing vein 1a shorter. Body length 4.8–5.1 mm; fore wing length 3.8–4.4 mm; antenna with 41 segments.

##### Diagnosis.

*Aleiodes asenjoi* is most similar to *A. angustus* (they are the only two species in this study that have a distinct and tubular fore wing vein 1a) but these two can be separated by the characters discussed in the diagnosis for *A. angustus* (above). *Aleiodes asenjoi* is also very similar to *A. bakeri* but it has the occipital carina more widely absent dorsally (Fig. 15), fore wing vein 1a present (absent in *A. bakeri*); vein (RS+M)a only weakly curved and almost straight (Fig. 14), fore wing stigma with an infuscate dot centrally (Fig. 14), and female with longer and wider ovipositor sheaths (Figs 14, 17).

##### Distribution.

Known from localities in Brazil and Peru.

##### Etymology.

The name is a patronym for Angelico Asenjo, the collector of the holotype specimen.

#### Aleiodes bahiensis

Taxon classificationAnimaliaHymenopteraBraconidae

Shimbori & Shaw sp. nov.

3E902BB2-EE56-5BA3-A1C3-5290E7D16301

http://zoobank.org/078FA6CA-5C55-443F-A0E6-7A5471AEA860

Figs 18–20

##### Type material.

Holotype, female (CNCI) “BRAZIL: Bahia, Encruzilhada, XI.1972 M. Alvarenga.”

Paratype, female (CNCI), same as holotype.

##### Description.

Body length 6.2–6.4 mm. Fore wing length 5.0–5.3 mm.

***Head.*** In dorsal view eye length/temple 4.0–5.0. Eye height/head width 0.43–0.45. Eye height/minimum distance between eyes 1.3–1.4. OD/POL 2.8–3.2. OD/OOL 3.2–3.3. Frons excavated. Frons lateral carina present. Occipital carina dorsally complete and nearly straight. Occiput in dorsal view nearly straight, not indented medially. Occipital carina ventrally meeting hypostomal carina. Mid-longitudinal crest at upper face present. Hypoclypeal depression/face width 0.36–0.37. Malar space/eye height 0.2. Face height/width 0.70–0.75. Clypeus height/width 0.70–0.75. Clypeus convex, granulate. Sculpture of head shiny granular-coriaceous. Face transversely rugose-striate around median crest.

***Antenna.*** Antennal segments 48–49. Antenna/body length 1.1. Scape/pedicel length 1.7–1.8. Length of first/second flagellomere 1.2. Fourth flagellomere length/apical width 1.7–1.8. Tip of apical segment of antenna nipple-shaped.

***Mesosoma.*** Length/height ~ 1.7. Width of mesoscutum/width of head 0.65. Mesoscutum length/width 1.2. Pronotal collar/vertex 0.9–1.0. Prescutellar sulcus with five distinct carinae. Mesoscutum posterior border with distinct complete carina. Metanotum with mid-longitudinal carina complete, connecting to a carinate pit posteriorly. Metanotum mid-pit present, delimited by carinae. Mid-longitudinal carina of propodeum present at basal 0.7, absent posteriorly. Ventral mid-line of mesopleuron set within shallow smooth sulcus. Pit at ventral mid-line weakly indicated. Notauli weakly indicated anteriorly, indistinctly crenulate. Sternaulus weakly indicated anteriorly, rugose. Sculpture of mesosoma mostly granulate. Pronotum granulate ventrally, pronotal groove mostly crenulate, short subventral longitudinal carina present. Mesopleuron mostly rugose. Subalar groove crenulate. Mid-posterior region of mesoscutum rugose, with a short mid-longitudinal carina posteriorly. Mesoscutellar trough entirely costate. Metanotum mostly smooth, with one or two pairs of lateral carinae. Propodeum mostly rugose.

***Wings.*** Fore wing: Stigma length/height 3.5. Vein r/2RS 1.1–1.2. Vein r/RS+Mb 1.5. Vein 3RSa/2RS 1.5–1.8. Vein 3RSa/2M 0.75–0.80. Vein 3RSa/3RSb 0.34–0.42. Vein 1CUa/1CUb 0.85–0.90. Vein 1CUa/2CUa 1.9. Vein 1cu-a weakly inclivous. Vein 1M weakly curved basally. Vein RS+Ma weakly curved. Vein M+CU virtually straight. Vein 1-1A nearly straight. Vein 1a absent. Second submarginal cell trapezoidal. Subbasal cell glabrous, with two parallel rows of short setae subapically, and a narrow patch of setae just below vein 1CUa. Basal cell mostly evenly setose, sparsely setose posteriorly. Hind wing: Vein RS bent at basal 0.3, with vein r present. Marginal cell narrowest at base. Vein M+CU/1M 1.5–1.6. Vein M+CU/r-m 1.3. Vein m-cu present, spectral. Vein m-cu position relative to vein r-m interstitial, or antefurcal. Vein 2-1A absent. Basal cell evenly, rather sparsely setose, posteriorly with small bare area.

***Hind legs.*** Femur length/width 4.7–5.5. Length of tibia/tarsi ~ 1.0. Length of basitarsus/tarsi 2–4 ~ 0.7. Sculpture of hind coxa dorsally mostly shiny granular-coriaceous, finely striate apically. Tarsal claws not pectinate.

***Metasoma.***T1 length/apical width 1.2–1.3. T2 length/apical width ~ 0.9. T3 length/apical width 0.6. Mid-longitudinal carina extending until basal 0.7 of T3. Metasoma sculpture T1 rugose, T2 and most of T3 striate-rugose, remainder terga granular-coriaceous. Ovipositor sheath/hind basitarsus ~ 0.4. Apex of ovipositor sheaths truncate; apical point absent.

***Color.*** Entirely yellowish brown, except for stemmaticum black. Wings weakly tinged brownish yellow; veins and stigma yellow except 1M, 1CUa, apex of 1-1A, r, 2RS, 3RS, 2M and part of 2CUb brown; faintly infuscate areas around veins 1M, r and 2CUa, and bellow apex of vein 1-1A.

**Male.** Unknown

##### Diagnosis.

**Figures 18–20.**
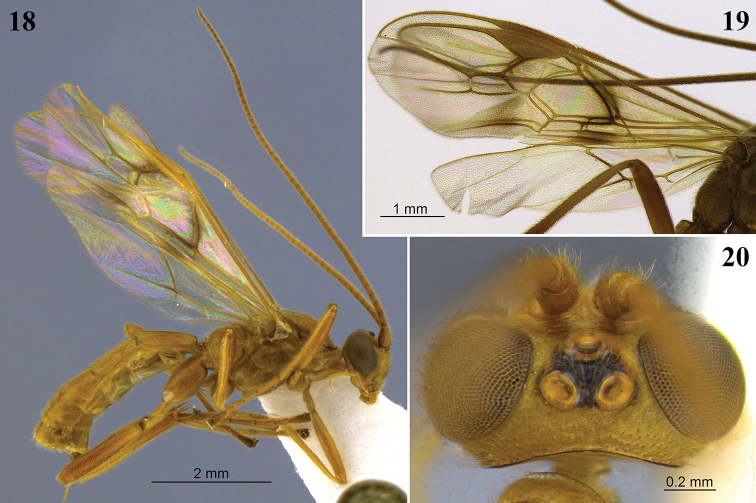
*Aleiodes bahiensis* sp. nov. **18** Lateral habitus **19** wings **20** head, dorsal view.

*Aleiodes bahiensis* is similar to *A. hyalinus* and *A. santarosensis*. These three species have the antenna entirely yellow (as in Fig. 18). *Aleiodes hyalinus* is easily distinguished by its entirely clear wings and evenly brown wing venation, while both *A. bahiensis* and *A. santarosensis* share similar wing markings: veins 1M, 1CUa, and part of 2CUb dark brown, darker than remaining veins, and the wing membrane around these veins, and below vein 1-1A apically, is weakly to distinctly infuscate (as in Fig. 19). *Aleiodes bahiensis* can be distinguished from *A. santarosensis* by having entirely yellow legs (Fig. 18), while in *A. santarosensis* the legs have tarsomeres 1–4 and at least the base of the tibia whitish yellow, contrasting with a brownish orange femur.

##### Distribution.

Known only from the type-locality in Bahia, Brazil.

##### Etymology.

The name *bahiensis* refers to Bahia State in northeastern Brazil, the type-locality of this species.

#### Aleiodes bakeri

Taxon classificationAnimaliaHymenopteraBraconidae

(Brues, 1912)

C5FC70F5-97B6-5070-B900-6A805236652E

Figs 21–24

Rhogas bakeri Brues, 1912: 222, fig 21.
Aleiodes bakeri Shenefelt, 1975: 1166.
not Aleiodes (Hemigyroneuron) bakeri Butcher & Quicke, 2011: 1417.

##### Type material examined.

Holotype, female (MCZ-Harvard). 7 labels: 1. “Rio Madeira, Brazil Mann & Baker.” 2. “Madeira-mamoré R.R. Co. Camp 39.” 3. “TYPE.” 4. “M.C.Z. H Type 29923.” 5. “*Rhogas bakeri* Brues.” 6. “MCZ Image Database.” 7 “MCZ-ENT 00029923.”

##### Non-type material examined.

BRAZIL: 2 females (CNCI), Encruzilhada, Bahia; 6 males (CNCI), Sinop, Mato Grosso; 25 females and 2 males (DCBU), PARNA Serra das Confusões, Caracolândia, Piauí; 3 females and 1 male (DCBU), PARNA Serra da Capivara, Coronel José Dias, Piauí; 3 females and 2 males (DCBU), PARNA Sete Cidades, Piracuruca, Piauí; 1 female (MZUSP), Buritis, Minas Gerais; 1 female (MZUSP), Cabeceiras, Goiás. PERU: 1 female (MUSM), CICRA, Madre de Dios.

##### Re-description of holotype.

Holotype in fair condition. All but the left front leg detached from body, two hind and two mid legs glued in a separate card, metasoma loose but still attached to body, both antennae broken before middle.

Body length 7.0 mm. Fore wing length 6.0 mm.

***Head.*** In dorsal view eye length/temple 3.2. Eye height/head width 0.36. Eye height/minimum distance between eyes 1.1. OD/POL 2.5. OD/OOL 2.5. Frons excavated. Frons lateral carina present. Occipital carina dorsally incomplete. Occiput in dorsal view nearly straight, not indented medially. Occipital carina not curved toward ocelli. Occipital carina ventrally meeting hypostomal carina. Mid-longitudinal crest at upper face present. Hypoclypeal depression/face width 0.35. Malar space/eye height 0.2. Face height/width 0.65. Clypeus height/width ~ 0.6. Clypeus convex, granulate. Sculpture of head shiny granular-coriaceous. Face weakly rugose, with bulging granulate area below crest, transversely rugose-striate around median crest.

***Antenna.*** Antennal segments (antenna broken). Antenna/body length unknown (antenna broken). Scape/pedicel length 2.0. Length of first/second flagellomere 1.3. Fourth flagellomere length/apical width 1.3.

***Mesosoma.*** Length/height 1.5–1.6. Width of mesoscutum/width of head 0.55. Mesoscutum length/width ~ 1.0. Pronotal collar/vertex 0.5. Prescutellar sulcus with complete mid-longitudinal carina, and a few irregular and incomplete carinae laterally. Mesoscutum posterior border with distinct complete carina. Metanotum with mid-longitudinal carina complete, connecting to a carinate pit posteriorly. Metanotum mid-pit present, delimited by carinae. Mid-longitudinal carina of propodeum present at basal 0.7, absent posteriorly. Ventral mid-line of mesopleuron smooth, without distinct sulcus; pit at ventral mid-line present, shallow. Notauli present anteriorly, shallow and weakly crenulate. Sternaulus absent. Sculpture of mesosoma mostly granulate. Metapleuron rugose posteriorly. Pronotum rugose laterally, pronotal groove curvedly crenulate anteriorly. Mesopleuron mostly rugose. Subalar groove crenulate. Mid-posterior region of mesoscutum destroyed by pin. Mesoscutellar trough entirely costate. Metanotum mostly smooth and weakly crenulate. Propodeum rugose posteriorly.

***Wings.*** Fore wing: Stigma length/height 3.6–3.9. Vein r/2RS 1.3. Vein r/RS+Mb 1.4. Vein 3RSa/2RS 1.3. Vein 3RSa/2M 0.8. Vein 3RSa/3RSb 0.3. Vein 1CUa/1CUb 0.9. Vein 1CUa/2CUa 1.8. Vein 1cu-a weakly inclivous. Vein 1M weakly curved basally. Vein RS+Ma distinctly curved. Vein M+CU virtually straight. Vein 1-1A weakly sinuate at apex. Vein 1a absent. Second submarginal cell short and trapezoidal. Subbasal cell glabrous, with two parallel rows of short setae subapically. Basal cell with more or less large glabrous region posteriorly, sometimes with sparse setae; costal and apical regions evenly setose. Hind wing: Vein RS bent at basal 0.3, with vein r present. Marginal cell narrowest at base. Vein M+CU/1M 1.9. Vein M+CU/r-m 1.6. Vein m-cu present, spectral. Vein m-cu position relative to vein r-m interstitial. Vein 2-1A absent. Basal cell evenly setose.

***Hind legs.*** Femur length/width 4.3. Length of tibia/tarsi ~ 1.0. Length of basitarsus/tarsi 2–4 0.75. Sculpture of hind coxa dorsally shiny granular-coriaceous. Tarsal claws not pectinate.

***Metasoma.***T1 length/apical width ~ 1.1. T2 length/apical width ~ 0.8. T3 length/apical width 0.6. Mid-longitudinal carina extending until basal 0.7 of T3. Metasoma sculpture of T1, T2, and basal 0.7 of T3 rugose-costate, sculpture weaker at T3, remainder metasoma smooth. Ovipositor sheath/hind basitarsus 0.3. Apex of ovipositor sheaths roughly rounded; apical point relatively long and curved.

***Color.*** Mostly pale honey yellow; all coxa, trochanter and trochantellus, and base of femur whitish (fore legs lighter than hind, hind coxa light yellow); stemmaticum and mandible tips brown; wings weakly tinged yellow, with two infuscate regions on fore wing, one around vein 1M, extending to a infuscate region below apex of subbasal cell, and another at stigma level, including the second submarginal cell and part of vein 2CUb (in original description the infuscate regions are described as cross-bands, maybe specimen lost color during the past 100 years; in holotype and in younger specimens the infuscate regions do not form cross bands. Instead there are infuscate regions around vein 1M, below apex of vein 1-1A, around vein r and veins forming the second submarginal cell, and around vein 2CUb medially); stigma brownish yellow without any dark spot; veins yellow, brown in the infuscate regions: veins 1M at basal ¾, 1CUa, apex of 1-1A, r, 2RS, 3RS, and 2CUb subapically.

##### Description of non-type specimens.

Body length 6.3–7.5 mm. Fore wing length 5.3–6.0 mm.

***Head.*** In dorsal view eye length/temple 3.2–4.1. Eye height/head width 0.36–0.42. Eye height/minimum distance between eyes 1.1–1.3. OD/POL 2.5–3.7. OD/OOL 2.5–4.0. Frons excavated. Frons lateral carina present. Occipital carina dorsally incomplete. Occiput in dorsal view weakly indented medially. Occipital carina not curved toward ocelli. Occipital carina ventrally meeting hypostomal carina. Mid-longitudinal crest at upper face present. Hypoclypeal depression/face width 0.32–0.37. Malar space/eye height 0.14–0.20. Face height/width 0.6–0.7. Clypeus height/width 0.57–0.67. Clypeus convex, granulate. Sculpture of head shiny granular-coriaceous. Face weakly rugose, with bulging granulate area below crest, transversely rugose-striate around median crest.

***Antenna.*** Antennal segments 46–51. Antenna/body length 1.1. Scape/pedicel length 2.0. Length of first/second flagellomere 1.2–1.3. Fourth flagellomere length/apical width 1.3–1.4. Tip of apical segment of antenna pointed.

***Mesosoma.*** Length/height 1.7–1.8. Width of mesoscutum/width of head 0.65–0.68. Mesoscutum length/width 1.1–1.2. Pronotal collar/vertex 0.6–0.7. Prescutellar sulcus with complete mid-longitudinal carina, and a few irregular and incomplete carinae laterally. Mesoscutum posterior border with distinct complete carina. Metanotum with mid-longitudinal carina complete, connecting to a carinate pit posteriorly. Metanotum mid-pit present, delimited by carinae. Mid-longitudinal carina of propodeum present at basal 0.7, absent posteriorly. Ventral mid-line of mesopleuron smooth, without distinct sulcus; pit at ventral mid-line present, shallow. Notauli present anteriorly, shallow and weakly crenulate. Sternaulus absent. Sculpture of mesosoma mostly granulate, metapleuron rugose posteriorly. Pronotum rugose laterally, pronotal groove sparsely crenulate anteriorly. Mesopleuron mostly rugose. Subalar groove crenulate. Mid-posterior region of mesoscutum rugose with long and irregular mid-longitudinal carina. Mesoscutellar trough entirely costate. Metanotum mostly smooth and weakly crenulate. Propodeum rugose posteriorly.

***Wings.*** Fore wing: Stigma length/height 3.7–4.0. Vein r/2RS 1.3–1.5. Vein r/RS+Mb 1.5–1.7. Vein 3RSa/2RS 1.4–1.6. Vein 3RSa/2M 0.82–0.85. Vein 3RSa/3RSb 0.37–0.40. Vein 1CUa/1CUb 0.9–1.0. Vein 1CUa/2CUa 1.6–2.0. Vein 1cu-a weakly inclivous, or nearly vertical. Vein 1M weakly curved basally. Vein RS+Ma distinctly curved. Vein M+CU virtually straight. Vein 1-1A weakly sinuate at apex. Second submarginal cell short and trapezoidal. Subbasal cell mostly glabrous, with two parallel rows of short setae subapically, and few scattered setae medially. Basal cell mostly evenly, rather sparsely setose, with narrow glabrous anal spot. Hind wing: Vein RS bent at basal 0.3, with vein r present. Marginal cell narrowest at base. Vein M+CU/1M 1.6–1.7. Vein M+CU/r-m 1.3–1.4. Vein m-cu present, spectral. Vein m-cu position relative to vein r-m interstitial, or antefurcal. Vein 2-1A absent. Basal cell evenly, rather sparsely setose, posteriorly with small bare area.

***Hind legs.*** Femur length/width 4.4–4.6. Length of tibia/tarsi 0.9–1.0. Length of basitarsus/tarsi 2–4 0.72–0.74. Sculpture of hind coxa dorsally shiny granular-coriaceous. Tarsal claws not pectinate.

***Metasoma.***T1 length/apical width 1.0–1.1. T2 length/apical width 0.8–0.9. T3 length/apical width 0.6–0.7. Mid-longitudinal carina extending until near apex of T3, or extending until basal 0.7 of T3. Metasoma sculpture T1, T2 and basal 0.7 of T3 rugose-costate, remainder metasoma smooth. Ovipositor sheath/hind basitarsus 0.27–0.45. Apex of ovipositor sheaths roughly rounded; apical point relatively long and curved.

***Color.*** Essentially as in holotype. Body color varying from brownish yellow to pale yellow. Some specimens have a brown subapical spot on the pterostigma.

**Male.** Essentially as in female. Body length 5.6–6.6 mm; fore wing length 4.3–5.4 mm; antenna with 48–50 segments.

##### Diagnosis.

The color patterns, body proportions, and other features of *Aleiodes bakeri* are similar to those in *A. nigristemmaticum* (Enderlein). The most useful characters to distinguish them are the occipital carina, which is incomplete at the vertex in *bakeri* (Fig. 24) but is complete in *nigristemmaticum*, and the hind wing venation, with vein M+CU being more than 2 × longer than 1M in *bakeri* (Fig. 23), as compared with ~ 1.5 × longer in *nigristemmaticum*. Specimens of *A. nigristemmaticum* have the antenna dark brown basally, lightening toward apex, as compared with entirely honey yellow in *A. bakeri* (Fig. 22). Three of the new species, *A. angustus*, *A. asenjoi*, and *A. mabelae*, also have a dorsally incomplete occipital carina. Two of these, *A. angustus* and *A. asenjoi*, are easily distinguished by having the fore wing vein 1a present (as in Fig. 1), while this vein is absent in *A. bakeri* (Fig. 2). *Aleiodes mabelae* can be distinguished by its longer fore wing second submarginal cell (Fig. 64) and the flagellum which is black at the base (Fig. 64). The second submarginal cell is comparatively shorter in *A. bakeri* (Figs 2, 23), and the flagellum is entirely the same color, yellow or orange, without being black basally (Fig. 22).

**Figures 21–24.**
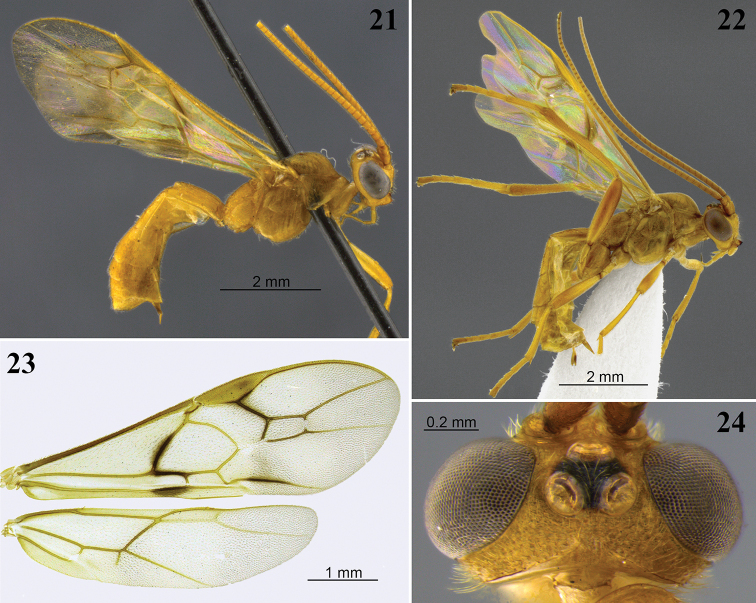
*Aleiodes bakeri* (Brues). **21** Holotype female, lateral view **22** non-type female, lateral view **23** wings **24** head, dorsal view.

##### Distribution.

*Aleiodes bakeri* is known from localities in Brazil and Peru.

##### Nomenclatural note.

Butcher and Quicke (2011) synonymized *Hemigyroneuron* Baker as a junior synonym of *Aleiodes* but retained *Hemigyroneuron* as a subgenus. The species Aleiodes (Hemigyroneuron) bakeri Butcher & Quicke, 2011 is not the same species as *Aleiodes bakeri* (Brues, 1912). Despite its assignment to a different subgenus, *Aleiodes bakeri* Butcher & Quicke, 2011 is a junior homonym of *Aleiodes bakeri* (Brues, 1912) and a replacement name is needed. There we hereby propose the new name, *Aleiodes buntikae* Shimbori & Shaw, nom. nov., for the species formerly called Aleiodes (Hemigyroneuron) bakeri Butcher & Quicke, 2011: p. 1417. The new name is a patronym in honor of Buntika Areekul-Butcher, author of the species formerly called Aleiodes (Hemigyroneuron) bakeri.

#### Aleiodes barrosi

Taxon classificationAnimaliaHymenopteraBraconidae

Shimbori & Shaw sp. nov.

4E251533-2192-5D21-B702-4A1AEF008919

http://zoobank.org/AF3B7B7D-C358-4F18-97CE-B38EE202F433

Figs 25–28

##### Type material.

Holotype, female (DCBU #21889) “Faz. Sto. Antônio do Paraíso, Itiquira, MT Armadilha Malaise 1.IX.1999 M.M. Barros & J.C.M. Lutz cols.”

Paratypes. 1 female (DCBU #22357) “Luís Antônio, SP, Brasil Estação Ecológica do Jataí, Luz 24.IV.2001 L.A. Joaquim col.”; 1 female (DCBU #21906), same data except “... Mata Ciliar 21°36'54"S, 47°47'02"W 04.I.2007 Armadilha Malaise 2 N.W. Perioto col.”, 1 female (DCBU #21907), same data except “01.III.2007”; 1 female (DCBU #28167) “Rio Branco, AC, Brasil, 09°59'30"S, 67°48'36"W Armadilha Malaise 01.I.2010 A.S. Soares col.”; 1 female (MUSM) “ PERU: MD, Parque Nacional Bahuaha – Sonene 70°0758.2"W, 13°11'38.7"S 347m 03–19.vi.2013 J. Grados Leg”; 1 female (MUSM) “PERU: MD, Rio Los Amigos, CICRA, Aeródromo, 276m, 12°33'36"S, 70°06'17.5"W 22–28.vii.2006, Light trap, A. Asenjo”; 1 male (MUSM) “PERU: CUSCO, La Convención, Echarate, C. Segakiato. 11°45'38.6"S, 73°14'57.7"W 908m. 01.ii.2011. M. Alvarado & E Rázuri.”; 1 female (INBIO) “Rancho Quemado, 200m, Peninsula de Osa, Prov. Puntarenas, Costa Rica Set 1992. F. Quesada L-S 292500, 51000”; 1 female (INBIO), same data except “Nov 1992”; 2 females (INBIO), same data except “… Oct 1992, M. Segura …”

##### Description.

Body length 7.6–9.4 mm. Fore wing length 7.0–8.6 mm.

***Head.*** In dorsal view eye length/temple 3.4–4.5. Eye height/head width 0.38–0.43. Eye height/minimum distance between eyes 1.1–1.3. OD/POL 3.0–5.0. OD/OOL 3.1–4.2. Frons excavated. Frons lateral carina present. Occipital carina dorsally complete, weakly curved. Occiput in dorsal view nearly straight, not indented medially. Occipital carina ventrally nearly touching hypostomal carina, or meeting hypostomal carina. Mid-longitudinal crest at upper face present. Hypoclypeal depression/face width 0.36–0.42. Malar space/eye height 0.11–0.19. Face height/width 0.8–0.9. Clypeus height/width 0.6–0.7. Clypeus convex, granulate. Sculpture of head shiny granular-coriaceous. Face transversely rugose-striate, medially granular-coriaceous below crest.

***Antenna.*** Antennal segments 58–67. Antenna/body length 1.1–1.2. Scape/pedicel length 2.3–2.9. Length of first/second flagellomere 1.0–1.2. Fourth flagellomere length/apical width 1.1–1.3. Tip of apical segment of antenna pointed.

***Mesosoma.*** Length/height 1.47–1.51. Width of mesoscutum/width of head 0.7–0.8. Mesoscutum length/width 1.0–1.2. Pronotal collar/vertex 0.5–0.7. Prescutellar sulcus with 3–5 distinct carinae. Mesoscutum posterior border with distinct complete carina. Metanotum with mid-longitudinal carina complete, connecting to a carinate pit posteriorly. Metanotum mid-pit present, delimited by carinae. Mid-longitudinal carina of propodeum complete, or nearly complete. Ventral mid-line of mesopleuron without sulcus anteriorly, shallow smooth sulcus present posteriorly; pit at ventral mid-line absent. Notauli weakly indicated anteriorly, indistinctly crenulate. Sternaulus absent. Sculpture of mesosoma mostly granulate. Pronotum rugose laterally, granulate ventrally, pronotal groove crenulate anteriorly, short subventral longitudinal carina present. Mesopleuron mostly rugose. Subalar groove sparsely crenulate. Mid-posterior region of mesoscutum rugose, with irregularly carinate notauli. Mesoscutellar trough entirely costate. Metanotum mostly smooth, with one or two pairs of lateral carinae. Propodeum mostly granulate, rugose posteriorly.

***Wings.*** Fore wing: Stigma length/height 3.6–3.8. Vein r/2RS 1.3–1.6. Vein r/RS+Mb 1.4–1.7. Vein 3RSa/2RS 1.1–1.3. Vein 3RSa/2M 0.76–0.85. Vein 3RSa/3RSb 0.24–0.29. Vein 1CUa/1CUb 0.45–0.60. Vein 1CUa/2CUa 0.9–1.1. Vein 1cu-a vertical. Vein 1M weakly curved basally, or nearly straight. Vein RS+Ma virtually straight. Vein M+CU weakly sinuate. Vein 1-1A strongly sinuate. Vein 1-1A distinctly changing thickness along apical half. Vein 1a absent. Second submarginal cell short and trapezoidal. Subbasal cell glabrous, with a narrow patch of setae subapically just below vein 1CUa. Basal cell with more or less large glabrous region posteriorly, sometimes with sparse setae; costal and apical regions evenly setose. Hind wing: Vein RS bent at basal 0.3, with vein r present. Marginal cell narrowest at base. Vein M+CU/1M 1.3–1.6. Vein M+CU/r-m 1.2–1.4. Vein m-cu present, partly tubular. Vein m-cu position relative to vein r-m interstitial, or antefurcal. Vein 2-1A absent. Basal cell sparsely setose, bare posteriorly.

***Hind legs.*** Femur length/width 4.5–5.0. Length of tibia/tarsi 0.9–1.0. Length of basitarsus/tarsi 2–4 0.7–0.8. Sculpture of hind coxa dorsally mostly shiny granular-coriaceous, finely striate apically. Tarsal claws not pectinate.

***Metasoma.***T1 length/apical width 1.0–1.2. T2 length/apical width 0.6–0.8. T3 length/apical width 0.5–0.7. Mid-longitudinal carina extending until basal 0.5 or 0.7 of T3. Metasoma sculpture T1 rugose, T2 and most of T3 striate-rugose, sculpture weaker at T3, remainder terga granular-coriaceous. Ovipositor sheath/hind basitarsus 0.36–0.56. Apex of ovipositor sheaths truncate; apical point very short, not distinctly visible in some paratypes.

***Color.*** Brownish yellow or pale honey yellow. Antenna dark brown basally, gradually lightening toward brown or brownish yellow apex. Legs with same color as body, rarely hind femur mostly dark brown. Wings tinged brown, stigma and most veins light brown; fore wing veins 1M, 1CU, apex of 1-1A, 2CUb medially, r, and veins of second submarginal cell dark brown. Ovipositor sheaths dark brown.

**Male.** The only male paratype is very similar to the females with dark brown hind femur. Body length 7.8 mm; fore wing length 6.7 mm; antenna with 44 antennomeres.

##### Diagnosis.

*Aleiodes barrosi* is similar to *A. joaquimi* in that both species have the first subdiscal cell relatively long and widening apically (Figs 27, 28), and both have the vein 1CUb 1.7–2.1 × longer than 1CUa (0.9–1.3 × in other species). These two species are easily separated by the mainly yellow body color in *A. barrosi* (deep reddish brown in *A. joaquimi*), yellow palpi and tegula (dark brown in *A. joaquimi*), and entirely yellow tibia and tarsi (tibia basally and tarsi 1–4 white in *A. joaquimi*). Additionally, the hind wing vein 2-1A is absent in *A. barrosi* (Fig. 28), but present in *A. joaquimi* (Fig. 58).

**Figures 25–28.**
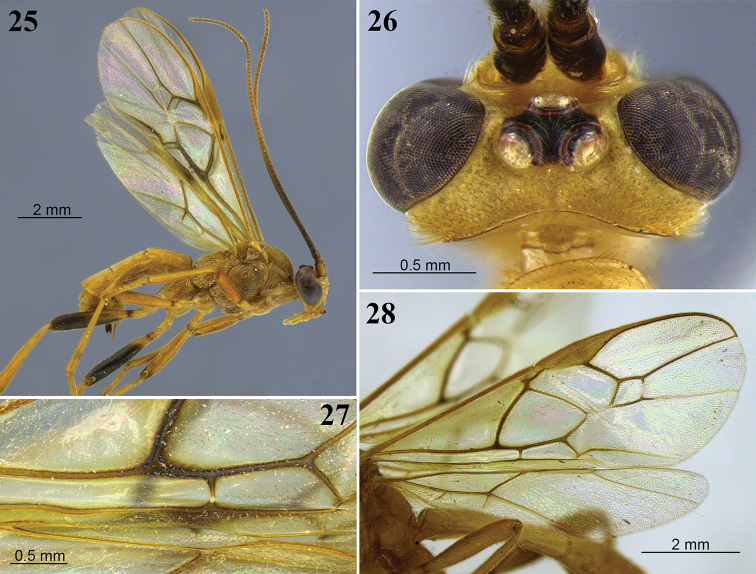
*Aleiodes barrosi* sp. nov. **25** lateral habitus **26** head, dorsal view **27** fore wing close-up basomedially **28** wings.

##### Distribution.

Known from localities in Brazil, Costa Rica, and Peru.

##### Etymology.

The name is a patronym for Marina Moraes Barros Lutz, one of the collectors of the holotype specimen.

#### Aleiodes brevicarina

Taxon classificationAnimaliaHymenopteraBraconidae

Shimbori & Shaw sp. nov.

61DDB503-62D1-5091-BA6B-7DA1A8104E52

http://zoobank.org/CC5929A9-D8ED-4EBE-AB4C-B4502DA8CC3E

Figs 29–32

##### Type material.

Holotype, female (DCBU #20780) top label: “FAZ. CANCHIM SÃO CARLOS – SP 29.III.1985 A.S. Soares col.”, bottom label “Mata (Luz) [handwritten].”

Paratypes. 3 females, 4 male (DCBU #s: 20778, 20779, 20781-20784, 20787), same as holotype; 1 male (DCBU #20785), same data except “11.II.1983”; 1 male (DCBU #20786), same data except “... cerrado, Varredura, 23.I.1981 N.W. Perioto col.”

##### Description.

Body length 7.8–8.1 mm. Fore wing length 6.4–7.0 mm.

***Head.*** In dorsal view eye length/temple 3.9–4.1. Eye height/head width 0.41. Eye height/minimum distance between eyes 1.2–1.3. OD/POL 3.7–5.0. OD/OOL 3.1–4.2. Frons excavated. Frons lateral carina present. Occipital carina dorsally complete and nearly straight. Occiput in dorsal view nearly straight, not indented medially. Occipital carina ventrally meeting hypostomal carina. Mid-longitudinal crest at upper face present. Hypoclypeal depression/face width 0.35–0.37. Malar space/eye height 0.16–0.17. Face height/width 0.72–0.77. Clypeus height/width 0.63–0.73. Clypeus convex, granulate. Sculpture of head shiny granular-coriaceous. Face transversely rugose-striate around median crest.

***Antenna.*** Antennal segments 55–56. Antenna/body length 1.1. Scape/pedicel length 2.0–2.1. Length of first/second flagellomere 1.2–1.3. Fourth flagellomere length/apical width 1.7–1.8. Tip of apical segment of antenna pointed.

***Mesosoma.*** Length/height 1.5–1.6. Width of mesoscutum/width of head 0.73–0.75. Mesoscutum length/width 1.0–1.1. Pronotal collar/vertex 0.7–0.8. Prescutellar sulcus with complete mid-longitudinal carina, and 2–4 pairs of rather incomplete carinae laterally. Mesoscutum posterior border with distinct complete carina. Metanotum with mid-longitudinal carina complete, connecting to a carinate pit posteriorly. Metanotum mid-pit present, delimited by carinae. Mid-longitudinal carina of propodeum present at basal 0.5 or less. Ventral mid-line of mesopleuron set within shallow smooth sulcus; pit at ventral mid-line absent. Notauli weakly indicated anteriorly, indistinctly crenulate. Sternaulus absent. Sculpture of mesosoma mostly granulate. Pronotum rugose laterally, pronotal groove sparsely crenulate anteriorly, short subventral longitudinal carina present. Mesopleuron mostly rugose. Subalar groove crenulate. Mid-posterior region of mesoscutum rugose, with irregular mid-longitudinal carina and a pair of irregular carinae along notauli. Mesoscutellar trough entirely costate. Metanotum mostly smooth and weakly crenulate, costate laterally. Propodeum mostly rugose.

***Wings*** (Figs 31, 32). Fore wing: Stigma length/height 3.6–3.7. Vein r/2RS 1.1–1.2. Vein r/RS+Mb 1.4. Vein 3RSa/2RS ~ 1.6. Vein 3RSa/2M 0.8. Vein 3RSa/3RSb ~ 0.4. Vein 1CUa/1CUb 0.9. Vein 1CUa/2CUa 1.8–1.9. Vein 1cu-a weakly inclivous. Vein 1M weakly curved basally. Vein RS+Ma sinuate. Vein M+CU virtually straight. Vein 1-1A nearly straight. Vein 1a absent. Second submarginal cell trapezoidal. Subbasal cell glabrous, with two parallel rows of short setae subapically, and a narrow patch of setae just below vein 1CUa. (Fig. 32). Basal cell mostly evenly setose, sparsely setose posteriorly. Hind wing: Vein RS bent at basal 0.3, with vein r present. Marginal cell narrowest at base. Vein M+CU/1M 1.5–1.8. Vein M+CU/r-m 1.2–1.4. Vein m-cu present and pigmented, although not tubular. Vein m-cu position relative to vein r-m interstitial or nearly so. Vein 2-1A absent. Basal cell evenly, rather sparsely setose, posteriorly with small bare area (Fig. 31).

**Figures 29–32.**
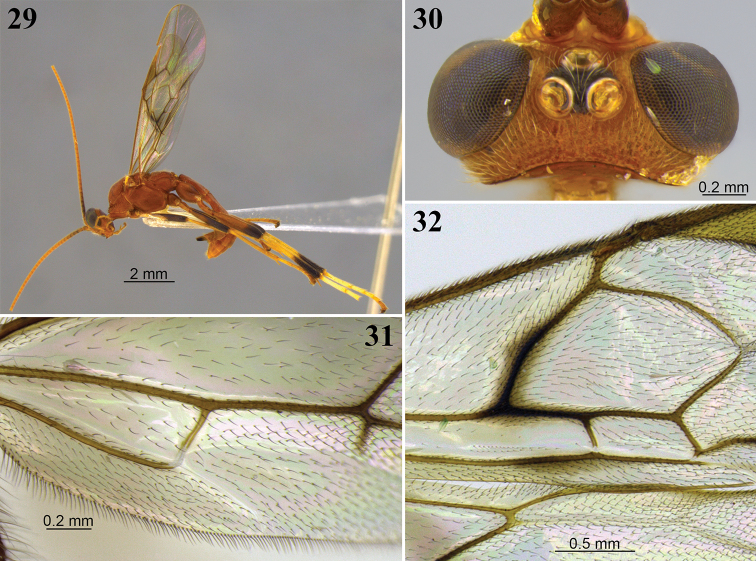
*Aleiodes brevicarina* sp. nov. **29** lateral habitus **30** head, dorsal view **31** hind wing close-up basally **32** wings medially.

***Hind legs.*** Femur length/width 4.8–5.5. Length of tibia/tarsi ~ 0.9. Length of basitarsus/tarsi 2–4 0.65–0.75. Sculpture of hind coxa dorsally granulate. Tarsal claws not pectinate.

***Metasoma.***T1 length/apical width 1.3–1.4. T2 length/apical width 0.8–1.0. T3 length/apical width 0.5–0.6. Mid-longitudinal carina extending until near apex of T3. Metasoma sculpture T1 rugose, T2 and most of T3 striate-rugose, sculpture weaker at T3, T4 granular-coriaceous, remainder of metasoma smooth. Ovipositor sheath/hind basitarsus ~ 0.37. Apex of ovipositor sheaths truncate; apical point absent.

***Color*** (Figs 29, 30). Brownish orange. Palpi yellow. Antenna mostly brownish orange, but basally brown and tip slightly darker. Wings weakly infuscate, veins brown, stigma yellow. Fore and middle legs with femur dark brown, tibia and tarsi brownish yellow or pale yellow; fifth tarsomeres light brownish orange. Hind legs with femur mostly dark brown apically, tibia pale yellow with apical ~ 0.3 dark brown, tarsi 1–4 mostly pale yellow, fifth tarsomeres light brown. Ovipositor sheaths dark brown.

**Male.** Essentially as in female except body length 7.0–8.1 mm; fore wing length 6.0–6.8 mm; antenna with 51–53.

##### Diagnosis.

*Aleiodes brevicarina* is one of a small group of species with similarly colored, distinctively banded hind legs (Fig. 29), including *A. joaquimi*, *A. maculosus*, and *A. ovatus*. This species differs from these other species with similarly banded hind legs in having propodeum with a very short longitudinal carina, less than half of its length.

##### Distribution.

Known only from type locality at Canchim Farm (Embrapa Pecuária Sudeste), São Paulo state, Brazil.

##### Etymology.

The name *brevicarina* is Latin for short ridge, being a reference to the short median carina on the propodeum in this species.

#### Aleiodes coariensis

Taxon classificationAnimaliaHymenopteraBraconidae

Shimbori & Shaw sp. nov.

65EACEE9-9AAB-523B-8A78-153096145608

http://zoobank.org/41AEFC83-8976-4A5E-B0C6-1620FF5CA038

Figs 33–36

##### Type material.

Holotype, female (DCBU #20788) “BR, AM, Coari, rio Urucu, Petrobras, ROC-29, 5–10/II/1992, P. Bührnneim. N.O. Aguiar & N.Fé col.”

Paratypes. 1 female (CNCI) “BRAZIL, Mato Grosso, Sinop, XI.1975 M. Alvarenga, Mal. Trap”; 1 female, 1 male (MUSM) “PERU: MD, Rio Los Amigos, CICRA, Aeródromo, 276m, 12°33'36"S, 70°06'17.5"W 22–28.vii.2006, Light trap, A. Asenjo”; 1 female (MUSM), same data except “… 380m … 2009, Manual, S. Carbonel”; 1 female (MUSM) “PERU: PU, Sandia, San Pedro de Putina Punco, P.N. Bahuaja Sonene 13°23'29.4"S, 69°29'00.1"W 322m 11–24.ix.2011 E. Guilhermo y E. Razuri.”

##### Description.

Body length 7.5–9.2 mm. Fore wing length 6.7–7.7 mm.

***Head*** (Fig. 36). In dorsal view eye length/temple 4.6–4.9. Eye height/head width 0.40–0.42. Eye height/minimum distance between eyes 1.2–1.4. OD/POL 2.4–3.4. OD/OOL 2.8–3.4. Frons excavated. Frons lateral carina present. Occipital carina dorsally complete, weakly curved. Occiput in dorsal view nearly straight, not indented medially. Occipital carina ventrally meeting hypostomal carina. Mid-longitudinal crest at upper face present. Hypoclypeal depression/face width 3.7–3.9. Malar space/eye height 0.18–0.19. Face height/width 0.84–0.87. Clypeus height/width 0.6–0.7. Clypeus convex, granulate. Sculpture of head shiny granular-coriaceous. Face transversely rugose-striate, medially granular-coriaceous below crest.

***Antenna.*** Antennal segments 57–59. Antenna/body length 1.3. Scape/pedicel length 2.2–2.5. Length of first/second flagellomere 1.1. Fourth flagellomere length/apical width 1.5–1.6. Tip of apical segment of antenna pointed.

***Mesosoma.*** Length/height ~ 1.7. Width of mesoscutum/width of head 0.7. Mesoscutum length/width ~ 1.0. Pronotal collar/vertex 0.8. Prescutellar sulcus with 5–7 distinct carinae. Mesoscutum posterior border with distinct complete carina. Metanotum with mid-longitudinal carina complete, connecting to a carinate pit posteriorly, or carina bisecting posterior pit, although weaker posteriorly. Metanotum mid-pit present, delimited by carinae. Mid-longitudinal carina of propodeum complete, or nearly complete. Ventral mid-line of mesopleuron without sulcus anteriorly, shallow smooth sulcus present posteriorly; pit at ventral mid-line absent. Notauli weakly indicated anteriorly, indistinctly crenulate. Sternaulus weakly indicated anteriorly, rugose. Sculpture of mesosoma mostly granulate. Metapleuron rugose posteriorly. Pronotum rugose laterally, pronotal groove sparsely crenulate anteriorly, short subventral longitudinal carina present. Mesopleuron rugose below subalar groove. Subalar groove crenulate. Mid-posterior region of mesoscutum rugose, with a short mid-longitudinal carina posteriorly. Mesoscutellar trough entirely costate. Metanotum costate, or mostly smooth and weakly crenulate. Propodeum mostly rugose.

***Wings*** (Fig. 35). Fore wing: Stigma length/height 3.6–3.7. Vein r/2RS 1.2–1.4. Vein r/RS+Mb 1.3–1.4. Vein 3RSa/2RS 1.5–1.7. Vein 3RSa/2M 0.8. Vein 3RSa/3RSb 0.3–0.4. Vein 1CUa/1CUb 0.85. Vein 1CUa/2CUa 1.6. Vein 1cu-a weakly inclivous. Vein 1M weakly curved basally. Vein RS+Ma distinctly curved. Vein M+CU virtually straight. Vein 1-1A very weakly sinuate apically. Vein 1a absent. Second submarginal cell trapezoidal. Subbasal cell glabrous, with two parallel rows of short setae subapically, and a row of setae just below vein 1CUa and M+CU apically, plus a row of setae apically just above vein 1-1A. Basal cell mostly evenly setose, although setae sparser posteriorly, rarely with more or less large glabrous region posteriorly. Hind wing: Vein RS bent at basal 0.3, with vein r present. Marginal cell narrowest at base. Vein M+CU/1M 1.7–1.8. Vein M+CU/r-m 1.3–1.4. Vein m-cu present and pigmented, although not tubular. Vein m-cu position relative to vein r-m interstitial, or just antefurcal. Vein 2-1A absent. Basal cell evenly, rather sparsely setose, posteriorly with small bare area.

***Hind legs.*** Femur length/width 5.0–5.3. Length of tibia/tarsi 0.85–0.95. Length of basitarsus/tarsi 2–4 0.75. Sculpture of hind coxa dorsally shiny granular-coriaceous. Tarsal claws not pectinate.

***Metasoma.***T1 length/apical width 1.1–1.2. T2 length/apical width 0.8–0.9. T3 length/apical width 0.65. Mid-longitudinal carina extending until basal 0.7 of T3. Metasoma sculpture T1 rugose, T2 and most of T3 striate-rugose, sculpture weaker at T3, remainder terga granular-coriaceous. Ovipositor sheath/hind basitarsus 0.3–0.5. Apex of ovipositor sheaths truncate; apical point absent.

***Color.*** Body entirely brownish yellow, including palpi and tegula. Antenna dark brown basally, gradually lightening toward light brown apex. All three femora apically dark brown, dark region larger at hind femur. Wings tinged yellow; most veins yellow, except vein 1M basally and 1CUa dark brown, apex of 1-1A brown, and veins r, 2RS, 3RS, 2M and part of 2CUb light brown; distinct infuscate spot around vein 1M, more faintly infuscate areas around veins r and 2CUa, and bellow apex of vein 1-1A; stigma varying from entirely yellow to mostly dark brown expect basal 0.3 yellow. Ovipositor sheaths dark brown.

**Male.** Essentially as in female with stigma mostly dark brown, although dark spot at stigma smaller. Body length 8.0–8.6 mm; fore wing 6.7–7.0 mm; antenna with 61 segments.

##### Diagnosis.

*Aleiodes coariensis* is the only species in the *A. bakeri* species subgroup with all femora at least partially marked with dark brown color (Fig. 34). There is also a distinctive infuscate spot on the fore wing near the base of vein 1M (Fig. 35). See the key for additional diagnostic characters.

**Figures 33–36.**
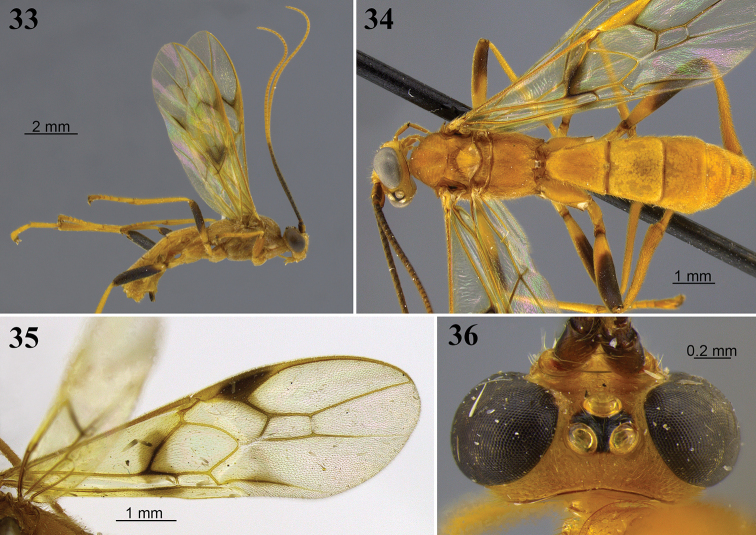
*Aleiodes coariensis* sp. nov. **33** lateral habitus **34** dorsal habitus **35** fore wing **36** head, dorsal view.

##### Distribution.

This species is known from localities in Brazil and Peru (Amazonian region).

##### Etymology.

The name *coariensis* refers to the municipality of Coari, in Amazonas State in northwestern Brazil, the type-locality of this species.

#### Aleiodes goiasensis

Taxon classificationAnimaliaHymenopteraBraconidae

Shimbori & Shaw sp. nov.

AC848933-D77E-5872-BBC6-97F51622450B

http://zoobank.org/D64CAA48-0F68-4784-87CE-AF235F500FE4

Figs 37–40

##### Type material.

Holotype, female (MZUSP) “Cabeceiras (Lagôa Formosa) Goiás 24–27.X.1964 Exp. Dep. Zool.”

Paratypes. 5 females (MZUSP), same as holotype; 1 female (DCBU #21878) “Brasil Pará Serra Norte N-1C. Pedra 5-IX-1983.”

##### Description.

Body length 7.2–8.8 mm. Fore wing length 6.3–7.6 mm.

***Head.*** In dorsal view eye length/temple 3.6–3.8. Eye height/head width 0.39–0.43. Eye height/minimum distance between eyes 1.1–1.3. OD/POL 2.6–2.8. OD/OOL 3.2–3.5. Frons excavated. Frons lateral carina present. Occipital carina dorsally complete and curved. Occiput in dorsal view weakly indented medially. Occipital carina ventrally meeting hypostomal carina. Mid-longitudinal crest at upper face present. Hypoclypeal depression/face width 0.34–0.38. Malar space/eye height 0.16–0.17. Face height/width 0.7. Clypeus height/width ~ 0.6. Clypeus convex, granulate. Sculpture of head mostly granulate, vertex granular-rugose, frons shiny granular-coriaceous. Face mostly transversely rugose-striate, granulate medially.

***Antenna.*** Antennal segments 54–61. Antenna/body length 1.1. Scape/pedicel length 2.4–2.6. Length of first/second flagellomere 1.2–1.3. Fourth flagellomere length/apical width 1.3–1.5. Tip of apical segment of antenna pointed.

***Mesosoma.*** Length/height 1.56–1.67. Width of mesoscutum/width of head 0.67–0.70. Mesoscutum length/width 1.2. Pronotal collar/vertex 0.7. Prescutellar sulcus with complete mid-longitudinal carina, and 2–4 pairs of rather incomplete carinae laterally. Mesoscutum posterior border with distinct complete carina. Metanotum with mid-longitudinal carina complete, connecting to a carinate pit posteriorly, sometimes bisecting posterior pit. Metanotum mid-pit present, delimited by carinae. Mid-longitudinal carina of propodeum present at basal 0.8, or complete. Ventral mid-line of mesopleuron set within shallow smooth sulcus; pit at ventral mid-line present, shallow. Notauli weakly indicated anteriorly, indistinctly crenulate. Sternaulus absent. Sculpture of mesosoma mostly granulate. Pronotum rugose laterally, pronotal groove curvedly crenulate anteriorly, short subventral longitudinal carina present. Mesopleuron mostly rugose. Subalar groove crenulate. Mid-posterior region of mesoscutum rugose with long and irregular mid-longitudinal carina. Mesoscutellar trough costate near scutellum. Metanotum mostly smooth, with one or two pairs of lateral carinae. Propodeum mostly rugose.

***Wings.*** Fore wing: Stigma length/height 3.2–3.6. Vein r/2RS 1.5. Vein r/RS+Mb 1.4–1.5. Vein 3RSa/2RS 1.4–1.5. Vein 3RSa/2M 0.8. Vein 3RSa/3RSb 0.31–0.34. Vein 1CUa/1CUb 0.8–0.9. Vein 1CUa/2CUa 1.6–1.7. Vein 1cu-a inclivous. Vein 1M weakly curved basally. Vein RS+Ma distinctly curved. Vein M+CU virtually straight. Vein 1-1A sinuate. Vein 1a absent. Second submarginal cell short and trapezoidal. Subbasal cell glabrous, with two parallel rows of short setae subapically, and a narrow patch of setae just below vein 1CUa. Basal cell with more or less large glabrous region posteriorly, sometimes with sparse setae; costal and apical regions evenly setose. Hind wing: Vein RS bent at basal 0.3, with vein r present. Marginal cell narrowest at base. Vein M+CU/1M 1.6–1.7. Vein M+CU/r-m 1.4. Vein m-cu present, spectral. Vein m-cu position relative to vein r-m interstitial, or antefurcal. Vein 2-1A absent. Basal cell sparsely setose, bare posteriorly.

***Hind legs.*** Femur length/width 4.7–4.8. Length of tibia/tarsi ~ 1.0. Length of basitarsus/tarsi 2–4 0.75. Sculpture of hind coxa dorsally granulate. Tarsal claws not pectinate.

***Metasoma.***T1 length/apical width ~ 1.1. T2 length/apical width 0.75. T3 length/apical width 0.6. Mid-longitudinal carina extending until basal 0.5 of T3. Metasoma sculpture T1 rugose, T2 and most of T3 striate-rugose, sculpture weaker at T3, remainder terga granular-coriaceous. Ovipositor sheath/hind basitarsus 0.1–0.2. Apex of ovipositor sheaths truncate; apical point absent.

***Color.*** Brownish orange. Stemmaticum black. Antenna dark brown basally, gradually lightening toward brownish yellow apex; pedicel dark brown; scape dark brown, ventrally yellow. Wings weakly tinged yellow; stigma and most veins yellow but veins 1M at basal 0.7, 1CUa, apex of 1-1A and of 2CUb, and sometimes vein r brown to dark brown; infuscate areas around base of vein 1M and below apex of vein 1-1A. Ovipositor sheaths dark brown.

**Male.** Unknown

##### Diagnosis.

*Aleiodes goiasensis* is similar to *A. nigristemmaticum* (Enderlein) but differs by having the fore wing vein r distinctly longer than 2RS (Fig. 39), the occipital carina distinctly curved mid-dorsally (Fig. 38), and the hind femur relatively shorter (4.7–4.8 × longer than wide). In contrast, in *A. nigristemmaticum* specimens the fore wing vein r is approximately equal to vein 2RS length, the occipital carina dorsally is mostly straight or only slightly bent, and the hind femur is 5.5–5.7 × longer than wide.

**Figures 37–40.**
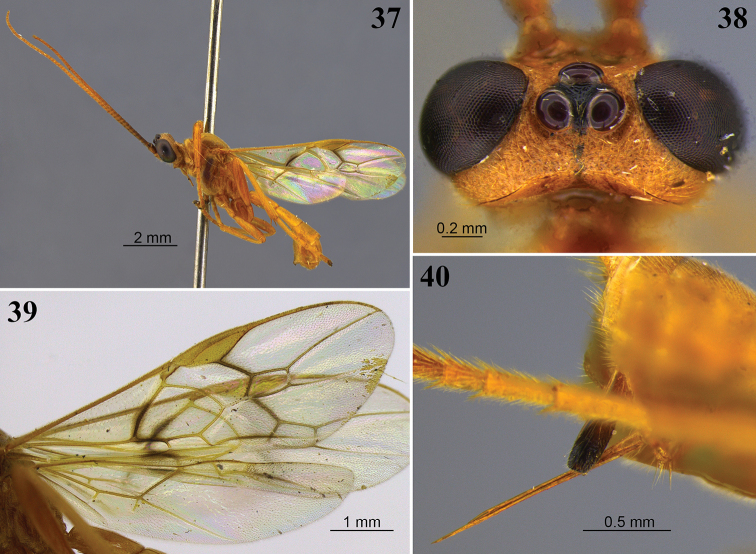
*Aleiodes goiasensis* sp. nov. **37** lateral habitus **38** head, dorsal view **39** wings **40** apex of metasoma showing ovipositor and sheath.

##### Distribution.

*Aleiodes goiasensis* is known only from central Brazil.

##### Etymology.

The name refers to Goiás, a state in mid-west Brazil, and the type locality for this new species.

#### Aleiodes gonodontivorus

Taxon classificationAnimaliaHymenopteraBraconidae

Shaw & Shimbori sp. nov.

20F3F0F6-8ADC-5444-98E4-133BB7774A27

http://zoobank.org/F7196D5E-362C-46CD-9E34-DB554180CC54

Figs 41–47

##### Type material.

Holotype, female (UWIM) “COSTA RICA: Puntarenas Pen. Osa, 23 km N. Pto. Jimenez, La Palma, 10m viii.ix.1991, P. Hanson Malaise, in large trees.”

Paratype data: 1 female (CNCI) Voucher: D.H. Janzen & W. Hallwachs, DB http://janzen.sas.upenn.edu, Area de Conservacion Guanacaste, COSTA RICA, 08-SRNP-56870, DHJPAR0029068. 17 females (pinned) with same data as except database code numbers as follows: 02-SRNP-15182; 02-SRNP-16572; 04-SRNP-22853; 05-SRNP-21738, DHJPAR0009352; 05-SRNP-57663, DHJPAR0009351; 06-SRNP-33504, DHJPAR0016434; 07-SRNP-21855, DHJPAR0021131; 07-SRNP-55246, DHJPAR0016925; 07-SRNP-55995, DHJPAR0021153; 07-SRNP-57169, DHJPAR0021156; 07-SRNP-55235, DHJPAR0016919; 07-SRNP-56915, DHJPAR0021154; 08-SRNP-21657, DHJPAR0028027; 08-SRNP-21975, DHJPAR0028034; 08-SRNP-21658, DHJPAR0028026; 08-SRNP-21742, DHJPAR0028035; 08-SRNP-56872, DHJPAR0028025 [BOLD ID: replace: ASHYE262-08; additional data: Sector Mundo Nuevo, Vado Huacas, 10.755 -85.391, 490 m, ex. *Gonodonta fulvangula* (Erebidae), 3.viii.2008, J. Cortez col.] (CNCI). 6 males (pinned) with same data except code numbers as follows: 90-SRNP-1226; 94-SRNP-6152; 07-SRNP-56881, DHJPAR0021155; 08-SRNP-21758, DHJPAR0028028; 08-SRNP-21980, DHJPAR0028029 (CNCI). 5 females (in alcohol vials) with same data except code numbers as follows: 05-SRNP-58906, DHJPAR0021181; 06-SRNP-22766, DHJPAR0029041; 08-SRNP-57558, DHJPAR0029063; 08-SRNP-56966, DHJPAR0029060; 08-SRNP-57556, DHJPAR0029062 (CNCI). 4 males (in alcohol vials) with same data except code numbers as follows: 06-SRNP-32956, DHJPAR0029042; 06-SRNP-32931, DHJPAR0029049; 08-SRNP-56881, DHJPAR0029066 [BOLD ID: replace: ASHYF744-09; additional data: Sector Mundo Nuevo, Vado Huacas, 10.755 -85.391, 490 m, ex. *Gonodonta fulvangula* (Erebidae), 4.viii.2008, D. Guadamuz col.]; 08-SRNP-56740, DHJPAR0029065 (CNCI). 1 female, Mexico, Campeche, Escárcega, El Tormento, 18°36'30.1"N, 90°48'45.7"W, ex. *Gonodonta nitidimacula* on *Piper amalago*, 25. 8. 2018, D. Campos. 1 male (INBIO) “P.N. Manuel Antonio, 80 m, Quepos, Prov. Punt., COSTA RICA, May 1993. G. Varela. L-S 370900, 448800”; 1 male (MZUSP) “Brasil: BA: Andarai, Mata Carrasco (Castanha), 13–14.XII.1990 Brandão, Diniz & Oliveira”; 1 female (DCBU #21872) “BIOTA – FAPESP Recife, PE, Brasil Pque. Estadual de Dois Irmãos 21.VII.2002 Varredura – Amostra 1 S.T.P. Amarante e equipe col.”; 1 female (DCBU #21873), same data except “... 22.VII.2002 ... Amostra 7.”

##### Description.

Body length 6.5–8.1 mm. Fore wing length 5.6–6.2 mm.

***Head*** (Figs 42, 43, 45). In dorsal view eye length/temple 3.6–4.5. Eye height/head width 0.41–0.43. Eye height/minimum distance between eyes 1.2–1.4. OD/POL 2.9–3.8. OD/OOL 2.3–3.8. Frons excavated. Frons lateral carina present; W-shaped carina present or absent, usually poorly defined. Occipital carina dorsally complete and curved (Fig. 45). Occiput in dorsal view weakly indented medially. Occipital carina ventrally meeting hypostomal carina. Mid-longitudinal crest at upper face present. Hypoclypeal depression/face width 0.35–0.39. Malar space/eye height 0.15–0.19. Face height/width 0.68–0.76. Clypeus height/width 0.67–0.69. Clypeus convex, granulate. Sculpture of head mostly granulate. Face transversely rugose-striate around median crest.

**Figures 41–47.**
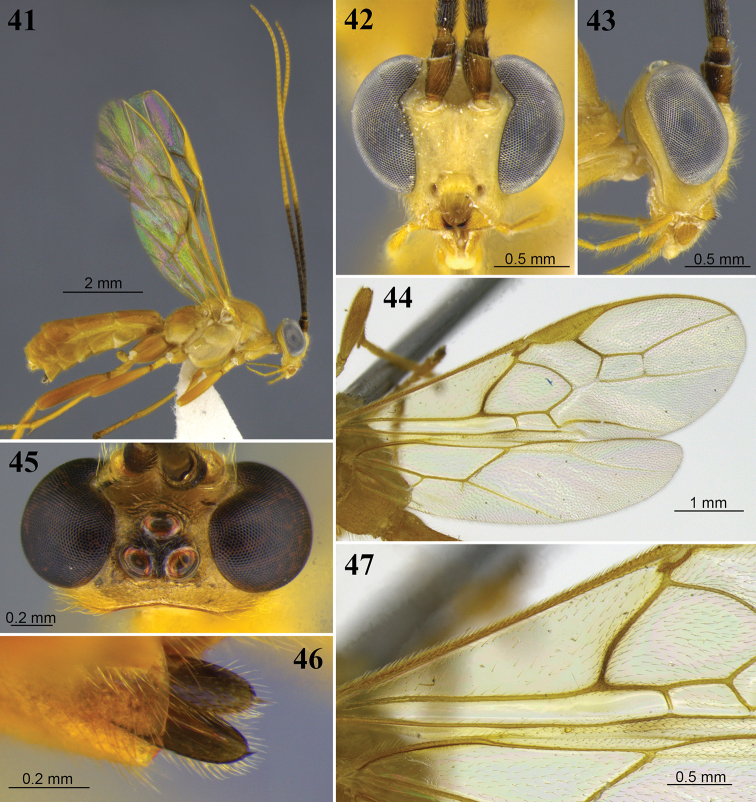
*Aleiodes gonodontivorus* sp. nov. **41** lateral habitus **42** head, anterior view **43** head, lateral view **44** wings **45** head, dorsal view **46** apex of metasoma showing ovipositor and sheath with apical point **47** fore wing basally.

***Antenna.*** Antennal segments 52–54. Antenna/body length 1.1–1.2. Scape/pedicel length 2.3–2.6. Length of first/second flagellomere 1.0–1.2. Fourth flagellomere length/apical width 1.5–1.6. Tip of apical segment of antenna pointed, or nipple-shaped.

***Mesosoma.*** Length/height ~ 1.6. Width of mesoscutum/width of head 0.7–0.8. Mesoscutum length/width ~ 1.1. Pronotal collar/vertex 0.9. Prescutellar sulcus with complete mid-longitudinal carina plus two or three pairs of lateral carinae more or less defined, or entirely costate, lateral carina oblique and nearly reaching anterior border. Mesoscutum posterior border with distinct complete carina. Metanotum with complete mid-longitudinal carina, sometimes interrupted at middle; carinate posterior pit sometimes bisected by carina. Metanotum mid-pit present, delimited by carinae. Mid-longitudinal carina of propodeum nearly complete. Ventral mid-line of mesopleuron set within shallow smooth sulcus; pit at ventral mid-line present, shallow. Notauli weakly indicated anteriorly, indistinctly crenulate. Sternaulus weakly indicated anteriorly, rugose. Sculpture of mesosoma mostly granulate. Pronotum granulate-rugose laterally, pronotal groove curvedly crenulate anteriorly, short subventral longitudinal carina present. Mesopleuron rugose below subalar groove. Subalar groove crenulate. Mid-posterior region of mesoscutum rugose, with a short mid-longitudinal carina posteriorly. Mesoscutellar trough entirely costate. Metanotum costate. Propodeum mostly rugose.

***Wings*** (Figs 44, 47). Fore wing: Stigma length/height 3.4–3.6. Vein r/2RS 1.2–1.3. Vein r/RS+Mb 1.2–1.4. Vein 3RSa/2RS 1.4–1.7. Vein 3RSa/2M 0.79–0.86. Vein 3RSa/3RSb 0.32–0.43. Vein 1CUa/1CUb 0.9–1.0. Vein 1CUa/2CUa 1.6–1.9. Vein 1cu-a weakly inclivous. Vein 1M weakly curved basally. Vein RS+Ma distinctly curved. Vein M+CU virtually straight. Vein 1-1A sinuate. Vein 1a absent. Second submarginal cell trapezoidal. Subbasal cell glabrous, with two parallel rows of short setae subapically, and a narrow patch of setae just below vein 1CUa. Basal cell with more or less large glabrous region posteriorly, sometimes with sparse setae; costal and apical regions evenly setose. Hind wing: Vein RS bent at basal 0.3, with vein r present. Marginal cell narrowest at base. Vein M+CU/1M 1.5–1.7. Vein M+CU/r-m 1.3–1.4. Vein m-cu present, spectral. Vein m-cu position relative to vein r-m interstitial, or antefurcal. Vein 2-1A absent. Basal cell sparsely setose, bare posteriorly.

***Hind legs.*** Femur length/width 5.0–5.3. Length of tibia/tarsi 0.9–1.0. Length of basitarsus/tarsi 2–4 0.70–0.75. Sculpture of hind coxa dorsally shiny granulate, apically striate. Tarsal claws not pectinate.

***Metasoma.***T1 length/apical width 1.1–1.2. T2 length/apical width 0.7–0.9. T3 length/apical width 0.5–0.6. Mid-longitudinal carina extending until basal 0.7 of T3. Metasoma sculpture T1, T2 and basal 0.7 of T3 rugose-costate, or sculpture weaker at T3, remainder terga granular-coriaceous. Ovipositor sheath/hind basitarsus 0.3–0.5. Apex of ovipositor sheaths roughly rounded with distinct apical point (Fig. 46).

***Color.*** Brownish yellow. Stemmaticum black. Antenna with basal 14–16 flagellomeres black, apical segments yellow; pedicel black; scape black, ventrally brownish yellow. Wings weakly tinged yellow; stigma pale yellow, most veins yellow but veins 1M at basal half, apex of 2CUb, and sometimes vein r brown; faint infuscate areas around base of vein 1M and below apex of vein 1-1A. Ovipositor sheaths dark brown.

**Male.** Essentially as in female, 10–16 black basal flagellomeres. Body length 6.6–7.3 mm; fore wing length 5.4–5.6 mm; antenna with 50 segments.

##### Diagnosis.

*Aleiodes gonodontivorus* resembles *A. nigristemmaticum* (Enderlein) but is readily recognizable by the distinctly and abruptly contrasting bicolored antenna (Fig. 41). In *A. nigristemmaticum* specimens the flagellum is dark basally but becomes gradually lighter over many flagellomeres. *Aleiodes gonodontivorus* may also be easily distinguished by the short second submarginal cell (Fig. 44), and the fore wing vein r being distinctly longer than vein 2RS (Fig. 44). In *A. nigristemmaticum* the veins r and 2RS are of similar length. *Aleiodes gonodontivorus* is also similar to *A. lidiae* but these two species can be easily separated by the characters given in couplet 17 of the key and they are also discussed in the diagnosis for *A. lidiae*.

##### Biology.

Parasitoids of caterpillars of *Gonodonta bidens* (Geyer) [8-SRNP-57556, 57558], *G. correcta* Walker [06-SRNP-32931], *G. fulvangula* (Geyer) [4-SRNP-22853; 7-SRNP-21855, 5691557169; 8-SRNP-21738, 21742, 21758, 21975, 21980, 56740, 56870, 56872, 56881, 56966], *G. immacula* (Guenée) [8-SRNP-58906; 90-SRNP-1226], *G. incurva* (Sepp) [2-SRNP-15182; 5-SRNP-57663; 6-SRNP-22766, 33504; 7-SRNP-55235, 55246, 55995; 8-SRNP-21657, 21658; 94-SRNP-6152], *G. maria* (Guenée) [7-SRNP-56881], *G. nitidimacula* Guenée, *G. pyrgo* (Cramer) [2-SRNP-16752], and *G. uxor* (Cramer) [6-SRNP-32956] (Erebidae, Calpinae), which feed on species of *Piper* (Piperaceae), *Annona* (Annonaceae) and on *Ocotea veraguensis* (Lauraceae).

##### Distribution.

*Aleiodes gonodontivorus* is known from localities in Costa Rica and Brazil.

##### Etymology.

The name is from *Gonodonta* Hubner, 1818 (a genus of moths in the family Erebidae and a recorded host for this new species), and the Latin word *vorus* meaning to eat or devour.

#### Aleiodes hyalinus

Taxon classificationAnimaliaHymenopteraBraconidae

Shimbori & Shaw sp. nov.

4C9CF723-A683-535D-9DA4-3BEDD626A2FD

http://zoobank.org/5D2F5A96-801A-481A-9609-17C89C3BD377

Figs 48–50

##### Type material.

Holotype, female (DCBU #21839) “Rio Mogi Guaçu Luís Antônio-SP luz, 28.XII.1989 L.A. Joaquim, col.”

Paratypes. 1 female (DCBU #21838), same as holotype; 1 male (DCBU #21840), same data except “18.II.1988”; 1 female (DCBU #21812) “Faz. Jacutinga São Carlos – SP 28.IX.1987, luz, U. Fernandes col.”; 1 male (DCBU #21815) “Faz. Canchim São Carlos – SP 29.III.1985 A.S. Soares, col.”; 1 male (DCBU #21813), same data except “3.II.1987”; 3 females, 1 male (CNCI) “BRAZIL, 960m Bahia, Encruzilhada XI.1972 M. Alvarenga”; 1 female (CNCI) “Brazil, Pedra Azul, M. Gerais XI.1972”; 1 female (DZUP) “Jundiaí do Sul, PR, Brasil Fazenda Monte Verde 30.XI.1986 Luminosa, Lev. Ent. PROFAUPAR.”

##### Description.

Body length 5.8–8.3 mm. Fore wing length 5.0–6.9 mm.

***Head.*** In dorsal view eye length/temple 4.6–6.0. Eye height/head width 0.41–0.43. Eye height/minimum distance between eyes 1.3–1.4. OD/POL 2.0–3.6. OD/OOL 3.7–9.0. Frons excavated. Frons lateral carina weakly indicated. Occipital carina dorsally complete, weakly curved. Occiput in dorsal view nearly straight, not indented medially. Occipital carina ventrally meeting hypostomal carina. Mid-longitudinal crest at upper face present. Hypoclypeal depression/face width 0.33–0.36. Malar space/eye height 0.15–0.19. Face height/width 0.72–0.77. Clypeus height/width 0.6–0.7. Clypeus convex, granulate. Sculpture of head mostly granular-coriaceous, vertex granular-rugose, frons shiny granular-coriaceous. Face transversely rugose-striate, medially granular-coriaceous below crest.

***Antenna.*** Antennal segments 47–54. Antenna/body length 1.1. Scape/pedicel length 1.8–2.1. Length of first/second flagellomere 1.1–1.2. Fourth flagellomere length/apical width 1.6–1.7. Tip of apical segment of antenna nipple-shaped.

***Mesosoma.*** Length/height 1.5–1.6. Width of mesoscutum/width of head 0.68–0.78. Mesoscutum length/width 1.0–1.1. Pronotal collar/vertex 0.67–0.75. Prescutellar sulcus with complete mid-longitudinal carina, and a few irregular and incomplete carinae laterally. Mesoscutum posterior border with distinct complete carina. Metanotum with mid-longitudinal carina complete, connecting to a carinate pit posteriorly, or with complete mid-longitudinal carina, sometimes interrupted at middle. Metanotum mid-pit present, delimited by carinae. Mid-longitudinal carina of propodeum present and basal 0.5 or less, or nearly complete. Ventral mid-line of mesopleuron set within shallow smooth sulcus. Pit at ventral mid-line present, or weakly indicated. Notauli weakly indicated anteriorly, indistinctly crenulate. Sternaulus absent. Sculpture of mesosoma mostly granulate. Pronotum granulate ventrally, pronotal groove mostly crenulate, short subventral longitudinal carina present. Mesopleuron mostly rugose. Subalar groove crenulate. Mid-posterior region of mesoscutum rugose, with a short mid-longitudinal carina posteriorly. Mesoscutellar trough costate near scutellum. Metanotum mostly smooth and weakly crenulate. Propodeum mostly rugose.

***Wings.*** Fore wing (Fig. 50): Stigma length/height 3.4–3.6. Vein r/2RS 1.1–1.3. Vein r/RS+Mb 1.2–1.4. Vein 3RSa/2RS 1.5–1.9. Vein 3RSa/2M 0.77–0.87. Vein 3RSa/3RSb 0.37–0.44. Vein 1CUa/1CUb 0.8–0.9. Vein 1CUa/2CUa 1.6–1.8. Vein 1cu-a inclivous. Vein 1M weakly, evenly curved. Vein RS+Ma distinctly curved. Vein M+CU weakly sinuate. Vein 1-1A weakly sinuate at apex. Second submarginal cell trapezoidal. Vein 1a absent. Subbasal cell glabrous, with two parallel rows of short setae subapically, and a line of setae just below most part of veins M+CU/1CUa. Basal cell mostly evenly setose, more sparsely setose posteriorly. Hind wing: Vein RS bent at basal 0.3, with vein r present. Marginal cell narrowest at base. Vein M+CU/1M 1.5. Vein M+CU/r-m 1.2–1.3. Vein m-cu present, spectral, or partly tubular. Vein m-cu position relative to vein r-m distinctly antefurcal. Vein 2-1A absent. Basal cell sparsely setose, bare posteriorly.

**Figures 48–50.**
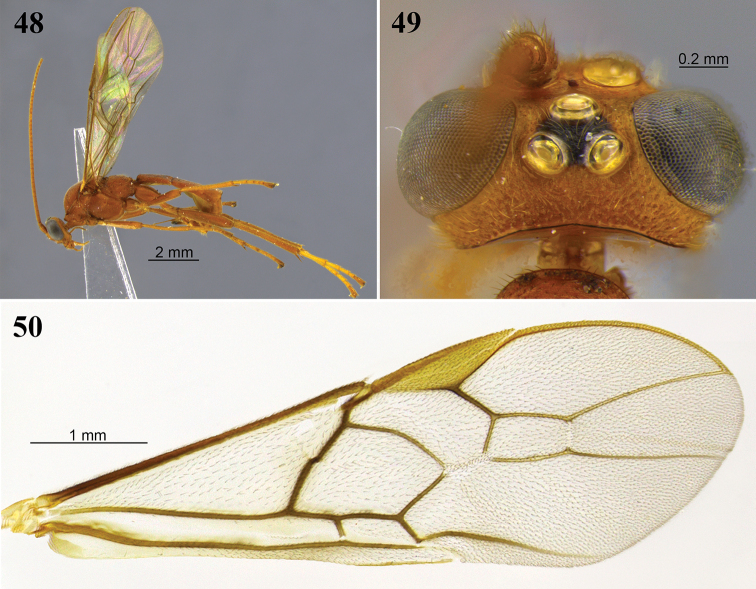
*Aleiodes hyalinus* sp. nov. **48** lateral habitus **49** head, dorsal view **50** fore wing.

***Hind legs.*** Femur length/width 4.4–4.8. Length of tibia/tarsi ~ 0.9. Length of basitarsus/tarsi 2–4 ~ 0.7. Sculpture of hind coxa dorsally granulate. Tarsal claws not pectinate.

***Metasoma.***T1 length/apical width 1.1–1.2. T2 length/apical width 0.75–0.80. T3 length/apical width 0.45–0.60. Mid-longitudinal carina extending until basal 0.7 of T3. Metasoma sculpture T1 rugose, T2 and most of T3 striate-rugose, remainder terga granular-coriaceous. Ovipositor sheath/hind basitarsus 0.42–0.54. Apex of ovipositor sheaths truncate, without apical point.

***Color*** (Fig. 48). Body reddish brown. Stemmaticum black (Fig. 49). Tegula dark brown. Wings subhyaline, veins light brown and stigma honey yellow (Fig. 50). Ovipositor sheaths dark brown.

**Male.** Essentially as in female. Body length 5.9–6.8 mm; fore wing length 4.9–5.7 mm; antenna with 45–51 segments.

##### Diagnosis.

*Aleiodes hyalinus* is most similar to *A. santarosensis*, but its wings are entirely subhyaline with all veins honey yellow to light brown, without distinct darker regions (Fig. 50). By contrast, the wings are tinged with yellow and with dark markings in *A. santarosensis* (Figs 76, 78). The body is brownish orange or reddish brown in *A. hyalinus* (Fig. 48), as opposed to being entirely yellow in *A. santarosensis* (Figs 76, 77). The ovipositor sheath is mostly dark brown to black in *A. hyalinus* (Fig. 48), as opposed to being light brown in *A. santarosensis* (Fig. 76), and the basal cell is evenly setose in *A. hyalinus* (Fig. 50), while in *A. santarosensis* the basal cell has a large bare area entirely lacking setae (Fig. 78).

##### Distribution.

*Aleiodes hyalinus* is known only from localities in Brazil.

##### Etymology.

The name *hyalinus* is Latin for glass-like or clear being a reference to the lack of coloration in the wings of this species.

#### Aleiodes inga

Taxon classificationAnimaliaHymenopteraBraconidae

Shimbori & Shaw sp. nov.

500CDA1A-94F0-5F83-B2B1-4B2E44696F20

http://zoobank.org/82072909-4984-4D62-A11C-EA89327F75CD

Figs 51–54

##### Type material.

Holotype data: Female (CNCI). Voucher: D.H. Janzen & W. Hallwachs, DB http://janzen.sas.upenn.edu, Area de Conservacion Guanacaste, COSTA RICA, 07-SRNP-42836, DHJPAR0029056.

Paratype: 1 female (pinned) with same data as holotype except database code number as follows: 07-SRNP-42756, DHJPAR0023529 [BOLD ID: replace: ASHYM281-08; additional data: Sector Rincon Rain Forest, Puente Rio Negro, 10.904 -85.303, 340 m, ex. *Rosema deolis* (Notodontinae), 15.xi.2007, J. Perez col.] (CNCI); 6 males (in alcohol vials) with same data as holotype except database code numbers as follows: 07-SRNP-43021, DHJPAR0029053; 07-SRNP-42801, DHJPAR0029052; 07-SRNP-34415, DHJPAR0029057 [BOLD ID: replace: ASHYE936-09; additional data: Sector Pitilla, Pasmompa, 11.019 -85.41, 440 m, ex. *Epitausa dilina* (Erebidae), 22.i.2008, C. Moraga and M. Rios col.]; 07-SRNP-42751, DHJPAR0029050 [BOLD ID: replace: ASHYE929-09; additional data: Sector Rincon Rain Forest, Puente Rio Negro, 10.904 -85.303, 340 m, ex. *Helia argentipes* (Erebidae), 21.xi.2007, M. Carmona col.]; 07-SRNP-66111, DHJPAR0028755; 07-SRNP-42758, DHJPAR0029055 [BOLD ID: replace: ASHYE934-09; additional data: Sector Rincon Rain Forest, Puente Rio Negro, 10.904 -85.303, 340 m, ex. *Helia argentipes* (Erebidae), 22.xi.2007, J. Perez col.] (CNCI).

##### Description.

Body length 6.6–6.7 mm. Fore wing length 5.3–5.5 mm.

***Head*** (Fig. 52). In dorsal view eye length/temple 4.8–5.1. Eye height/head width 0.44–0.46. Eye height/minimum distance between eyes 1.4–1.5. OD/POL 2.5. OD/OOL 3.3. Frons excavated. Frons lateral carina absent. Occipital carina dorsally complete, weakly curved. Occiput in dorsal view nearly straight, not indented medially. Occipital carina ventrally meeting hypostomal carina. Mid-longitudinal crest at upper face present. Hypoclypeal depression/face width 0.37. Malar space/eye height 0.18–0.19. Face height/width 0.81–0.85. Clypeus height/width 0.70–0.75. Clypeus convex, granulate. Sculpture of head shiny granular-coriaceous. Face mostly granular-coriaceous, transversely rugose-striate around median crest.

***Antenna.*** Antennal segments 51–54. Antenna/body length 1.1–1.2. Scape/pedicel length 2.1–2.4. Length of first/second flagellomere 1.1. Fourth flagellomere length/apical width 1.6. Tip of apical segment of antenna pointed.

***Mesosoma.*** Length/height 1.6–1.7. Width of mesoscutum/width of head 0.66–0.69. Mesoscutum length/width ~ 1.1. Pronotal collar/vertex 0.78–0.83. Prescutellar sulcus with complete mid-longitudinal carina, and a few irregular and incomplete carinae laterally. Mesoscutum posterior border with distinct complete carina. Metanotum with mid-longitudinal carina complete, connecting to a carinate pit posteriorly, sometimes bisecting posterior pit. Metanotum mid-pit present, delimited by carinae. Mid-longitudinal carina of propodeum nearly complete. Ventral mid-line of mesopleuron without sulcus anteriorly, shallow smooth sulcus present posteriorly; pit at ventral mid-line weakly indicated. Notauli weakly indicated anteriorly, indistinctly crenulate. Sternaulus weakly indicated anteriorly, rugose. Sculpture of mesosoma mostly granulate. Pronotum with pronotal groove mostly crenulate, short subventral longitudinal carina present. Mesopleuron rugose below subalar groove. Subalar groove sparsely crenulate. Mid-posterior region of mesoscutum rugose. Mesoscutellar trough entirely costate. Metanotum mostly smooth, with one or two pairs of lateral carinae. Propodeum mostly rugose.

***Wings.*** Fore wing: Stigma length/height 3.5–3.7. Vein r/2RS 1.1–1.3. Vein r/RS+Mb 1.2–1.3. Vein 3RSa/2RS 1.9–2.0. Vein 3RSa/2M 0.83. Vein 3RSa/3RSb 0.5. Vein 1CUa/1CUb 0.8–0.9. Vein 1CUa/2CUa 1.8–1.9. Vein 1cu-a nearly vertical. Vein 1M weakly curved basally. Vein RS+Ma weakly curved. Vein M+CU virtually straight. Vein 1-1A weakly sinuate at apex. Vein 1a absent. Second submarginal cell long and trapezoidal. Subbasal cell glabrous, with two parallel rows of short setae subapically, and a narrow patch of setae just below vein 1CUa. Basal cell mostly evenly setose, sparsely setose posteriorly, with a bare spot posteriorly. Hind wing: Vein RS bent at basal 0.3, with vein r present. Marginal cell narrowest at base. Vein M+CU/1M 1.4–1.6. Vein M+CU/r-m 1.4–1.5. Vein m-cu present, spectral. Vein m-cu position relative to vein r-m antefurcal. Vein 2-1A absent. Basal cell sparsely setose, bare posteriorly.

***Hind legs.*** Femur length/width 4.3–4.4. Length of tibia/tarsi ~ 0.9. Length of basitarsus/tarsi 2–4 0.70–0.75. Sculpture of hind coxa dorsally granulate. Tarsal claws not pectinate.

***Metasoma.***T1 length/apical width 1.2–1.3. T2 length/apical width 0.85. T3 length/apical width 0.60–0.65. Mid-longitudinal carina extending until basal 0.5 of T3. Metasoma sculpture T1 rugose, T2 and most of T3 striate-rugose, remainder terga granular-coriaceous. Ovipositor sheath/hind basitarsus 0.3. Apex of ovipositor sheaths truncate; apical point absent.

**Figures 51–54.**
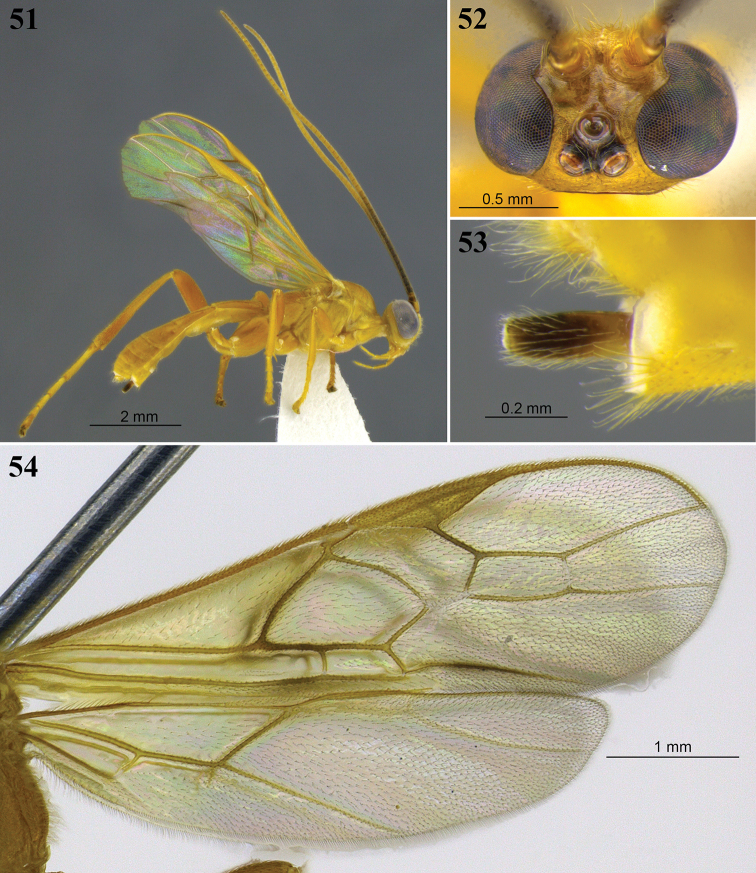
*Aleiodes inga* sp. nov. **51** lateral habitus **52** head, dorsal view **53** apex of metasoma showing ovipositor and truncate sheath without apical point **54** wings.

***Color.*** (Figs 51–53) Brownish yellow. Stemmaticum black. Antenna dark brown basally, gradually lightening toward brownish yellow apex; pedicel dark brown; scape dark brown, ventrally yellow. Wings weakly tinged yellow; stigma yellow; most veins yellow but veins 1M at basal half, apex of 2CUb, and sometimes vein r brown; faint infuscate areas around base of vein 1M and below apex of vein 1-1A. Ovipositor sheaths dark brown (Fig. 53).

**Male.** Essentially as in female. Body length 6.5–6.7 mm; fore wing 5.2 mm; antenna with 51–52 segments.

##### Diagnosis.

*Aleiodes inga* is unique within the subgroup in having the fore wing vein r longer than 2RS and much shorter than 3RSa, the second submarginal cell rectangular and comparatively long (Fig. 54) and the frons without lateral carina (Fig. 52). Other characters are similar to *A. nigristemmaticum*.

##### Biology.

The most common host caterpillar for this species is *Helia argentipes* (Walker) (Erebidae, Erebinae) [7-SRNP-42758, 42751, 42801, 42836], but there are also records from *Epitausa dilina* (Herrich-Schäffer) (Erebidae, Eulepidotinae) [7-SRNP-34415], feeding on *Inga edulis* and *I. oerstediana* (Fabaceae), and *Letis mycerina* (Cramer) [10-SRNP-65094] (Erebidae) feeding on *Inga oerstediana*. A database record from caterpillars of the Área de Conservación Guanacaste (http://janzen.sas.upenn.edu) of *Rosema deolis* (Cramer) (Notodontidae) [7-SRNP-42756] is refuted based on the morphology of the accompanying mummy.

##### Distribution.

This species is only known from northwest Costa Rica.

##### Etymology.

The name is a reference to *Inga*, a genus of small tropical trees in the Fabaceae family and the recorded host plant for some Erebidae host caterpillars of this new species.

#### Aleiodes joaquimi

Taxon classificationAnimaliaHymenopteraBraconidae

Shimbori & Shaw sp. nov.

408C54BE-5AE5-597D-9455-7CD6946E5468

http://zoobank.org/3C90EA2C-3A02-4C98-812D-040FA74537D0

Figs 55–58

##### Type material.

Holotype, female (DCBU #20794) “Sta. Maria Madalena, RJ, Brasil. P.E. Desengano 18.IV.2002 (luz) L.A. Joaquim & S.A. Soares cols.”

Paratype, female (CNCI) “BRAZIL: Bahia, Encruzilhada, XI.1972 M. Alvarenga.”

##### Description.

Body length 8.6–9.0 mm. Fore wing length 7.0–7.2 mm.

***Head.*** In dorsal view eye length/temple 4.1. Eye height/head width 0.39–0.43. Eye height/minimum distance between eyes 1.1–1.3. OD/POL 3.2–3.4. OD/OOL 2.6–3.4. Frons excavated. Frons lateral carina present in addition to W-shaped carina. Occipital carina dorsally complete and curved. Occiput in dorsal view weakly indented medially. Occipital carina ventrally nearly touching hypostomal carina. Mid-longitudinal crest at upper face present. Hypoclypeal depression/face width 0.4. Malar space/eye height 0.11–0.14. Face height/width 0.85. Clypeus height/width 0.69. Clypeus convex, granulate. Sculpture of head shiny granular-coriaceous. Face transversely rugose-striate, medially granular-coriaceous below crest.

***Antenna.*** Antenna with 59 antennomeres. Antenna/body length 1.0–1.1. Scape/pedicel length 2.5. Length of first/second flagellomere 1.1. Fourth flagellomere length/apical width 1.1. Tip of apical flagellomere pointed.

***Mesosoma.*** Length/height 1.45–1.50. Width of mesoscutum/width of head 0.7–0.8. Mesoscutum length/width ~ 1.0. Pronotal collar/vertex 0.6–0.7. Prescutellar sulcus with complete mid-longitudinal carina, and a few irregular and incomplete carinae laterally. Mesoscutum posterior border with distinct complete carina. Metanotum with mid-longitudinal carina complete, connecting to a carinate pit posteriorly. Metanotum mid-pit present, delimited by carinae. Mid-longitudinal carina of propodeum complete. Ventral mid-line of mesopleuron smooth, without distinct sulcus; pit at ventral mid-line weakly indicated. Notauli weakly indicated anteriorly, indistinctly crenulate. Sternaulus absent. Sculpture of mesosoma mostly granulate. Pronotum pronotal groove strongly crenulate anteriorly, short subventral longitudinal carina present. Mesopleuron mostly rugose. Subalar groove crenulate. Mid-posterior region of mesoscutum rugose with long and irregular mid-longitudinal carina. Mesoscutellar trough entirely costate. Metanotum mostly smooth, with one or two pairs of lateral carinae. Propodeum mostly rugose.

***Wings.*** Fore wing: Stigma length/height 3.3–3.5. Vein r/2RS 1.6. Vein r/RS+Mb 1.4–1.5. Vein 3RSa/2RS 1.2–1.3. Vein 3RSa/2M 0.71–0.75. Vein 3RSa/3RSb 0.24. Vein 1CUa/1CUb 0.6. Vein 1CUa/2CUa 1.1–1.2. Vein 1cu-a vertical. Vein 1M weakly, evenly curved. Vein RS+Ma virtually straight. Vein M+CU weakly sinuate. Vein 1-1A strongly sinuate. Vein 1a absent. Second submarginal cell short and trapezoidal. Subbasal cell glabrous, with a row of setae just below vein 1CUa and a row of setae apically just above vein 1-1A. Basal cell with more or less large glabrous region posteriorly, sometimes with sparse setae; costal and apical regions evenly setose. Hind wing: Vein RS bent at basal 0.3, with vein r present. Marginal cell narrowest at base. Vein M+CU/1M 1.6. Vein M+CU/r-m 1.4–1.5. Vein m-cu present and pigmented, although not tubular. Vein m-cu position relative to vein r-m interstitial. Vein 2-1A present, although very short (Fig. 58). Basal cell sparsely setose, bare posteriorly.

**Figures 55–58.**
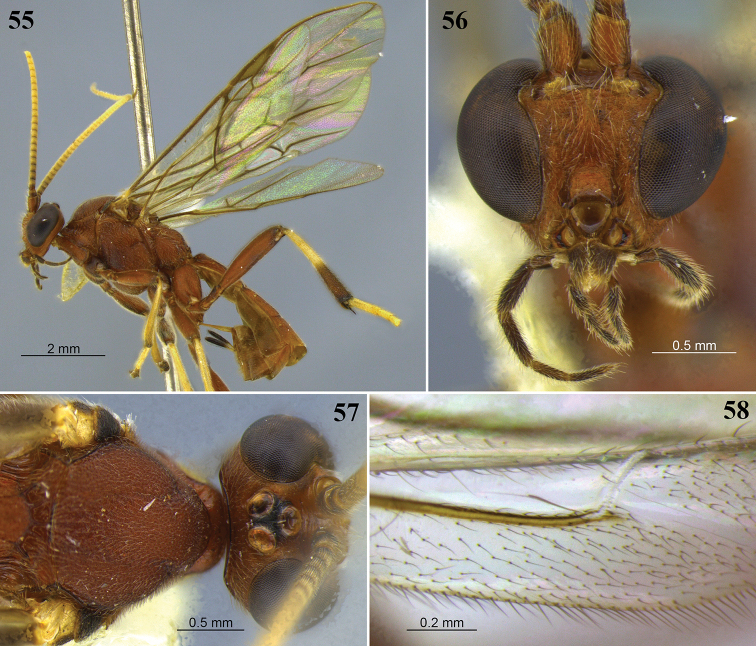
*Aleiodes joaquimi* sp. nov. **55** lateral habitus **56** head, anterior view **57** head, dorsal view **58** hind wing, posteriorly.

***Hind legs.*** Femur length/width 4.8–5.3. Length of tibia/tarsi ~ 1.0. Length of basitarsus/tarsi 2–4 ~ 0.8. Sculpture of hind coxa dorsally granulate. Tarsal claws not pectinate.

***Metasoma.***T1 length/apical width ~ 1.2. T2 length/apical width 0.7. T3 length/apical width 0.55. Mid-longitudinal carina extending until T2 or basal 0.5 of T3. Metasoma sculpture: T1–2 rugose-striate, T3 granulate, remainder metasoma smooth. Ovipositor sheath/hind basitarsus 0.5–0.6. Apex of ovipositor sheaths roughly rounded; apical point present, although very short.

***Color*** (Figs 55–57). Dark reddish brown. Palpi and tegula dark brown. Antenna mostly pale yellow, apex and base brown. All tibiae pale yellow with dark reddish brown apex, dark region larger in posterior legs; tarsi 1–4 whitish yellow, fifth tarsomere dark brown. Wings weakly tinged brown, veins brown, no infuscate regions. Ovipositor sheaths dark brown.

**Male.** Unknown

##### Diagnosis.

*Aleiodes joaquimi* differs from similar species with banded hind legs by its deep reddish brown color (Figs 55–57), absence of infuscate spots on wings (Fig. 55), hind wing vein 2-1A present, although short (Fig. 58), and vein 1CUb relatively long, ~ 1.7 times longer than vein 1CUa (no more than 1.25 times in other species). It is most similar to *A. barrosi*, and the differences between these two species are discussed in the diagnosis for *A. barrosi*.

##### Distribution.

The Atlantic Forest in Bahia and Rio de Janeiro states in Brazil

##### Etymology.

The name is an honorary patronym for Luiz A. Joaquim, one of the collectors of the holotype specimen.

#### Aleiodes lidiae

Taxon classificationAnimaliaHymenopteraBraconidae

Shimbori & Shaw sp. nov.

864ADA07-C0F4-589C-9A37-B115E4B8FA5F

http://zoobank.org/0120A76E-85EC-4F3E-B5A8-E61704045AB1

Figs 59–63

##### Type material.

Holotype, female (MUSM) “PERU: MD, Madama, 12°29'3846"S, 65°1'34"W, 182m [19–20], vii.2009, M. Alvarado.”

Paratypes, 1 female (MUSM) “PERU: CU, La Convención, Echarate, CC. Timpia. 72°49'34.56"S, 12°06'47.04"W 519m. 20–21.x.2009. Light. M. Alvarado y E Rázuri.” 1 female (MUSM) “PERU: JU, Pachitea River-System, Stat. Panguana am. Rio Llullapichis, trop. Tiefland-Regenwald. 260m. 9°37'S, 74°56'W, 2020.x.2009, G. Riedel.”

##### Description.

Body length 6.7–7.8 mm. Fore wing length 6.5–7.0 mm.

***Head*** (Figs 60, 61). In dorsal view eye length/temple 4.0–5.0. Eye height/head width 0.41–0.43. Eye height/minimum distance between eyes 1.2–1.4. OD/POL 2.5–3.0. OD/OOL 2.5–4.0. Frons excavated. Frons lateral carina present. Occipital carina dorsally complete, weakly curved. Occiput in dorsal view nearly straight, not indented medially. Occipital carina ventrally meeting hypostomal carina. Mid-longitudinal crest at upper face present. Hypoclypeal depression/face width 0.35–0.39. Malar space/eye height 0.16–0.17. Face height/width 0.76–0.82. Clypeus height/width 0.7. Clypeus convex, granulate. Sculpture of head shiny granular-coriaceous. Face weakly rugose, transversely rugose-striate around median crest.

**Figures 59–63.**
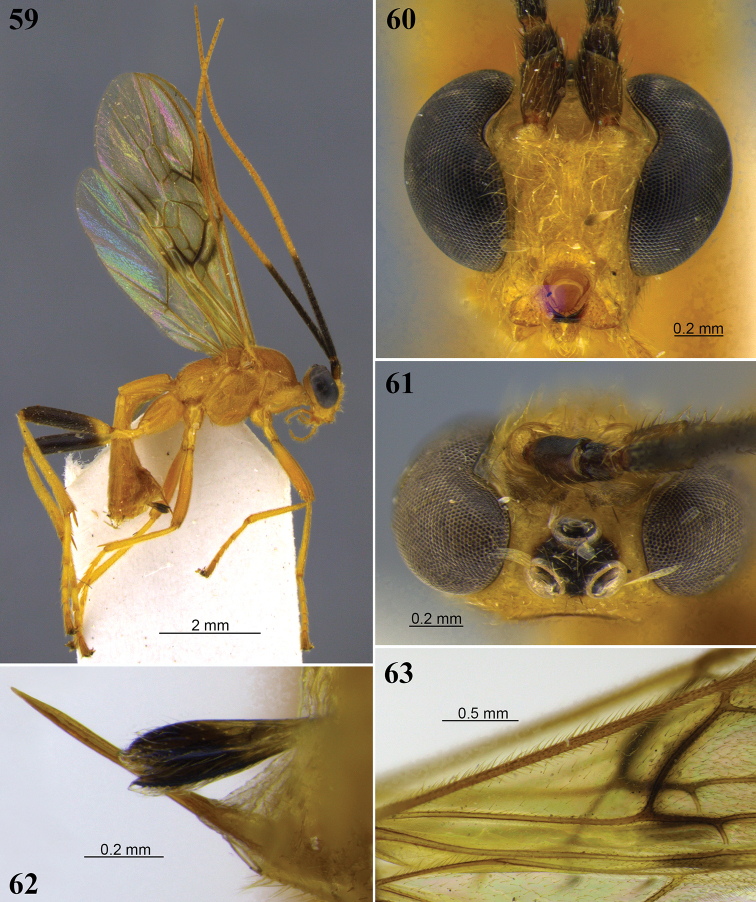
*Aleiodes lidiae* sp. nov. **59** lateral habitus **60** head, anterior view **61** head, dorsal view **62** Apex of metasoma showing ovipositor and sheath with apical point **63** wings, basally.

***Antenna.*** Antennal segments 53–56. Antenna/body length 1.2. Scape/pedicel length 1.9–2.0. Length of first/second flagellomere 1.1–1.2. Fourth flagellomere length/apical width 1.3–1.5. Tip of apical segment of antenna pointed.

***Mesosoma.*** Length/height 1.7–1.8. Width of mesoscutum/width of head 0.7. Mesoscutum length/width 1.2. Pronotal collar/vertex 0.8. Prescutellar sulcus with 3–5 distinct carinae. Mesoscutum posterior border with distinct complete carina. Metanotum with mid-longitudinal carina complete, connecting to a carinate pit posteriorly, carina bisecting posterior pit, although weaker posteriorly. Metanotum mid-pit present, delimited by carinae. Mid-longitudinal carina of propodeum present at basal 0.7, absent posteriorly, or nearly complete. Ventral mid-line of mesopleuron set within shallow smooth sulcus; pit at ventral mid-line present, shallow. Notauli weakly indicated anteriorly, indistinctly crenulate. Sternaulus weakly indicated anteriorly, rugose. Sculpture of mesosoma mostly granulate. Metapleuron rugose posteriorly. Pronotum rugose laterally, pronotal groove sparsely crenulate anteriorly, short subventral longitudinal carina present. Mesopleuron rugose below subalar groove. Subalar groove crenulate. Mid-posterior region of mesoscutum rugose, with a short mid-longitudinal carina posteriorly. Mesoscutellar trough entirely costate. Metanotum mostly smooth, with one or two pairs of lateral carinae. Propodeum mostly rugose.

***Wings.*** Fore wing: Stigma length/height 3.3. Vein r/2RS 1.2–1.4. Vein r/RS+Mb 1.3–1.4. Vein 3RSa/2RS ~ 1.7. Vein 3RSa/2M 0.86–0.88. Vein 3RSa/3RSb 0.40–0.44. Vein 1CUa/1CUb 1.0. Vein 1CUa/2CUa 1.7–1.9. Vein 1cu-a weakly inclivous. Vein 1M weakly curved basally. Vein RS+Ma distinctly curved. Vein M+CU virtually straight. Vein 1-1A nearly straight. Vein 1a absent. Second submarginal cell trapezoidal. Subbasal cell glabrous, with two parallel rows of short setae subapically, a row of setae just below of vein 1CUa and M+CU apically, plus a row of setae apically just above vein 1-1A. Basal cell mostly glabrous, setose below costal vein and around dark spot near vein 1M. Hind wing: Vein RS bent at basal 0.3, with vein r present. Marginal cell narrowest at base. Vein M+CU/1M 1.3–1.4. Vein M+CU/r-m 1.2. Vein m-cu present, spectral. Vein m-cu position relative to vein r-m antefurcal. Vein 2-1A absent. Basal cell sparsely setose, bare posteriorly.

***Hind legs.*** Femur length/width 4.8–5.0. Length of tibia/tarsi 0.96. Length of basitarsus/tarsi 2–4 ~ 0.7. Sculpture of hind coxa dorsally shiny granular-coriaceous. Tarsal claws not pectinate.

***Metasoma.***T1 length/apical width 1.0–1.1. T2 length/apical width 0.7–0.9. T3 length/apical width 0.5–0.6. Mid-longitudinal carina extending until basal 0.7 of T3. Metasoma sculpture T1 rugose, T2 and most of T3 striate-rugose, sculpture weaker at T3, remainder terga granular-coriaceous. Ovipositor sheath/hind basitarsus 0.3–0.5. Apex of ovipositor sheaths roughly rounded; apical point present, distinct (Fig. 62).

***Color*** (Figs 59–61). Brownish yellow. Stemmaticum black. Antenna with basal 11–13 flagellomeres black, apical segments yellow; pedicel black; scape black, ventrally brownish yellow. Wings tinged yellow; stigma and most veins orange to yellow; veins 1M, 1CUa, apex of 1-1A and r dark brown, veins 2RS, 3RS and 2M sometimes brown, apex of 2CUb brown; infuscate areas around base of vein 1M and below apex of vein 1-1A. Hind femur mostly dark brown, roughly basal 0.2 ventrally and 0.25 dorsally brownish orange. Ovipositor sheaths dark brown.

**Male.** Essentially as in female. Body length 6.8 mm; fore wing length 5.6 mm; antenna broken.

##### Diagnosis.

*Aleiodes lidiae* is most similar to *A. gonodontivorus*, but differing by having the hind femur mostly dark brown (Fig. 59) and conspicuous infuscate spots on the fore wing (Figs 59, 63). It also resembles *A. andinus*. The differences between these two species are discussed in the diagnosis given for *A. andinus*.

##### Distribution.

This species in known only from localities in Peru.

##### Etymology.

The name is an honorary patronym for our friend and fellow braconidologist, Lidia Sulca.

#### Aleiodes mabelae

Taxon classificationAnimaliaHymenopteraBraconidae

Shimbori & Shaw sp. nov.

BE369670-F20B-56D2-BC18-0EA7002AF1BD

http://zoobank.org/70539E94-7397-4489-8377-CAB3FCEAFD8A

Figs 64 , 65

##### Type material.

Holotype, female (MUSM) “PERU: CUSCO, La Convención, Echarate, C. Segakiato. 11°45'38.6"S, 73°14'57.7"W 908m. 01.ii.2011. M. Alvarado & E Rázuri.”

Paratypes. 2 females (MUSM) same as holotype except “28.ii.92011”; 1 female (MUSM), same as holotype except “… C.C. Timpia. 72°49'34.56"/ 12°06'47.04" 519m. 20–21.x.2009. Light …”; 1 female (MUSM) “PERU: JU, Satipo, San Andres, 11.33056S, 074.68665W, 975 m. 24.x.2012; UV&MV lights; E. Nearms & S. Carbonel”; 1 male (MUSM) “PERU: UC, Coronel Portillo, Puerto Alegre 19.vii.2008 08°44'7"S, 74°09'5"W 196m M. Alvarado; 1 male (MUSM) “PERU: MD, Rio Los Amigos, CICRA, 270m 1–16.ix.2007 12°34'8"S, 70°06'0"W Manual collect. S. Carbonel”.

##### Description.

Body length 6.9–7.5 mm. Fore wing length 6.1–6.8 mm.

***Head.*** In dorsal view eye length/temple 3.3–3.6. Eye height/head width 0.39–0.43. Eye height/minimum distance between eyes 1.1–1.2. OD/POL 2.0–2.7. OD/OOL 2.0–2.2. Frons weakly excavated. Frons lateral carina absent, or very weakly indicated. Occipital carina dorsally incomplete. Occiput in dorsal view nearly straight, not indented medially. Occipital carina not curved toward ocelli. Occipital carina ventrally meeting hypostomal carina. Mid-longitudinal crest at upper face present. Hypoclypeal depression/face width 0.31–0.35. Malar space/eye height 0.21–0.23. Face height/width 0.70–0.75. Clypeus height/width 0.63–0.69. Clypeus convex, granulate. Sculpture of head shiny granular-coriaceous. Face transversely rugose-striate, medially granular-coriaceous below crest.

***Antenna.*** Antennal segments 51–53. Antenna/body length 1.2. Scape/pedicel length 2.5–2.6. Length of first/second flagellomere 1.1–1.3. Fourth flagellomere length/apical width 1.6–1.7. Tip of apical segment of antenna pointed.

***Mesosoma.*** Length/height ~ 1.6. Width of mesoscutum/width of head 0.72–0.77. Mesoscutum length/width 1.0–1.1. Pronotal collar/vertex 0.7–0.8. Prescutellar sulcus with complete mid-longitudinal carina, and a few irregular and incomplete carinae laterally. Mesoscutum posterior border with distinct complete carina. Metanotum with complete mid-longitudinal carina, sometimes interrupted at middle. Metanotum mid-pit present, delimited by carinae. Mid-longitudinal carina of propodeum complete, sometimes irregular apically. Ventral mid-line of mesopleuron without sulcus anteriorly, shallow smooth sulcus present posteriorly; pit at ventral mid-line absent. Notauli weakly indicated anteriorly, indistinctly crenulate. Sternaulus weakly indicated anteriorly, rugose. Sculpture of mesosoma mostly granulate. Pronotum mostly smooth, granulate ventrally, pronotal groove entirely crenulate. Mesopleuron rugose below subalar groove. Subalar groove crenulate. Mid-posterior region of mesoscutum rugose, with a short mid-longitudinal carina posteriorly. Mesoscutellar trough weakly costate laterally. Metanotum mostly smooth, with one or two pairs of lateral carinae. Propodeum mostly granulate, rugose posteriorly.

***Wings.*** Fore wing: Stigma length/height 3.5–3.8. Vein r/2RS 0.75–0.85. Vein r/RS+Mb 1.0–1.2. Vein 3RSa/2RS 1.7–1.8. Vein 3RSa/2M 0.86–0.94. Vein 3RSa/3RSb 0.43–0.48. Vein 1CUa/1CUb 0.9–1.1. Vein 1CUa/2CUa 1.65–1.75. Vein 1cu-a vertical or weakly reclivous. Vein 1M nearly straight. Vein RS+Ma virtually straight. Vein M+CU virtually straight. Vein 1-1A nearly straight. Vein 1a absent. Second submarginal cell rectangular. Subbasal cell mostly glabrous, with sparse setae basally, a small setose patch at the infuscate region bellow vein 1CUa, and two or three irregular rows of short setae subapically above vein 1-1A. Basal cell evenly setose. Hind wing: Vein RS bent at basal 0.3, with vein r present. Marginal cell narrowest at base. Vein M+CU/1M 1.4–1.5. Vein M+CU/r-m 1.6–1.8. Vein m-cu present and pigmented, although not tubular. Vein m-cu position relative to vein r-m distinctly antefurcal. Vein 2-1A absent. Basal cell evenly and rather sparsely setose, with a small bare spot posteriorly.

***Hind legs.*** Femur length/width 4.7–5.1. Length of tibia/tarsi ~ 1.0. Length of basitarsus/tarsi 2–4 0.81–0.88. Sculpture of hind coxa dorsally shiny granular-coriaceous. Tarsal claws not pectinate.

***Metasoma.***T1 length/apical width 1.1–1.2. T2 length/apical width 0.8–0.9. T3 length/apical width 0.6–0.7. Mid-longitudinal carina extending until basal 0.5 of T3. Metasoma sculpture T1 rugose, T2 and most of T3 striate-rugose, remainder metasoma smooth. Ovipositor sheath/hind basitarsus 0.36–0.56. Ovipositor sheaths relatively narrow and truncate at apex; apical point very short, in most specimens hardly visible.

**Figures 64, 65.**
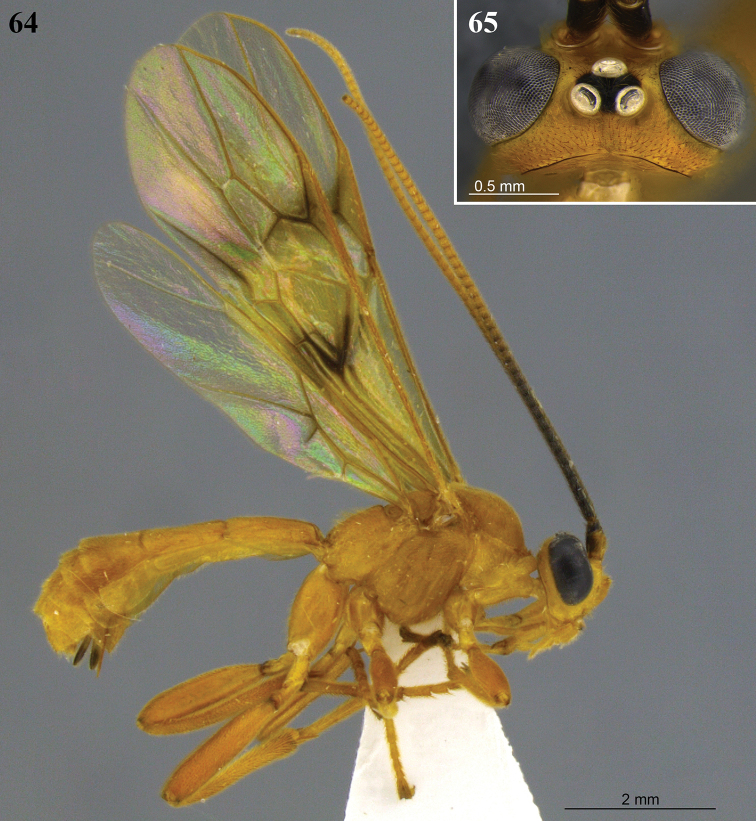
*Aleiodes mabelae* sp. nov. **64** lateral habitus **65** head, dorsal view showing interrupted occipital carina.

***Color*** (Fig. 64). Brownish yellow. Antenna dark brown basally, gradually lightening toward yellow to light brown apex; scape ventrally lighter. Apex of hind tibia darker apically, varying from dark brown to only faintly darker; hind femur, and sometimes of mid femur, dark brown in some specimens. Tarsal claws brown. Wings tinged yellow; most veins yellow, infuscate spots at fore wing veins 1M/1CUa, r, apex of 1-1A and 2CUb, membrane around these veins distinctly infuscate; stigma mostly dark brown or entirely brownish yellow. Ovipositor sheaths dark brown.

**Male.** Essentially as in females with dark stigma and apex of hind femur and tibia dark brown. Body length 7.0 mm, fore wing length 5.5 mm; antenna broken, with 33+ segments.

##### Diagnosis.

*Aleiodes mabelae* is similar to *A. bakeri* in having the occipital carina interrupted mid-dorsally (Fig. 65), and the vein 1a absent from the fore wing. However, these two species are readily separated by the color of the antenna, which is dark brown basally in *A. mabelae* (Fig. 64) but entirely brownish yellow in *A. bakeri* (Figs 21, 22). Also, the longitudinal carina of the propodeum is complete in *A. mabelae*, whereas it is incomplete in *A. bakeri*.

##### Comments.

Specimens collected at higher elevations (~ 900–1000 m) have the stigma and all legs yellow, while specimens from lower elevations (~ 200–500 m) have the stigma mostly dark brown, and the apex of the hind tibia and femur dark brown.

##### Distribution.

This species is known only from localities in Peru.

##### Etymology.

This species is named in honor to our friend, and fellow entomologist, Mabel Alvarado, collector of most of the type specimens of this new species.

#### Aleiodes maculosus

Taxon classificationAnimaliaHymenopteraBraconidae

Shimbori & Shaw sp. nov.

FC821C3A-DA91-526C-B80C-5A0193A49EEF

http://zoobank.org/D8493855-F91E-49AB-A512-733343B00DFD

Figs 66–68

##### Type material.

Holotype, female (CNCI) “BRAZIL, Encruzilhada, 980m, Bahia, XI.1975, M. Alvarenga”

Paratypes. 1 female (CNCI), same as holotype; 2 males (DCBU #s: 20789, 20793) “FAZ. CANCHIM SÃO CARLOS – SP luz 3.II.1984 A.S. Soares col.”; 1 male (DCBU #20790), same data except “29.III.1985”; 1 male (DCBU #20791), same data except “... cerrado, Varredura, 23.I.1981 N.W. Perioto col”; 1 male (DZUP) “Fênix, PR, Brasil Res. Est. ITCF Arm. Luminosa 3.X.1986 Projeto PROFAUPAR”; 1 male (CNCI) “Nova Teutonia 27°11'S, 52°23'W Brazil, 300–500m 3-IX-1948 Fritz Plaumann.”

##### Description.

Body length 8.0–8.1 mm. Fore wing length 6.4–6.9 mm.

***Head*** (Figs 67, 68). In dorsal view eye length/temple 4.6–5.0. Eye height/head width 0.39–0.43. Eye height/minimum distance between eyes 1.3. OD/POL 5.5–6.5. OD/OOL 3.7–4.3. Frons excavated. Frons lateral carina present in addition to W-shaped carina. Occipital carina dorsally complete and nearly straight, or weakly bent mid-dorsally. Occiput in dorsal view nearly straight, not indented medially. Occipital carina (complete). Occipital carina ventrally nearly touching hypostomal carina. Mid-longitudinal crest at upper face present. Hypoclypeal depression/face width 0.35. Malar space/eye height 0.16–0.18. Face height/width 0.77. Clypeus height/width 0.67–0.69. Clypeus convex, granulate. Sculpture of head shiny granular-coriaceous. Face weakly rugose, with bulging granulate are above clypeus and below crest, transversely rugose-striate around median crest.

**Figures 66–68.**
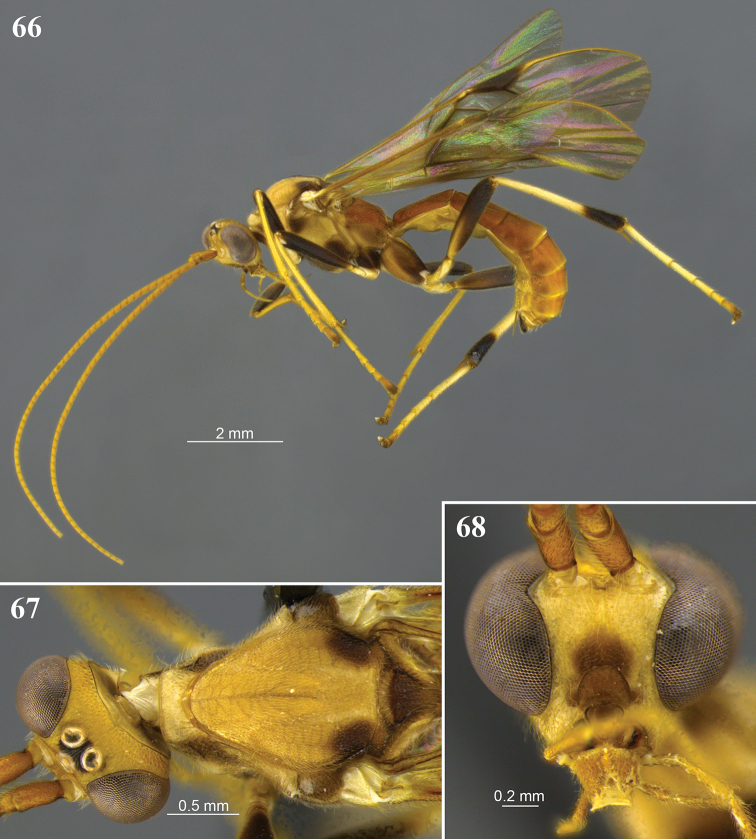
*Aleiodes maculosus* sp. nov. **66** lateral habitus **67** head and anterior mesosoma, dorsal view **68** head, anterior view.

***Antenna.*** Antennal segments 55. Antenna/body length 1.1. Scape/pedicel length 2.1. Length of first/second flagellomere 1.1. Fourth flagellomere length/apical width 1.9–2.0. Tip of apical segment of antenna nipple-shaped.

***Mesosoma.*** Length/height ~ 1.7. Width of mesoscutum/width of head 0.71–0.75. Mesoscutum length/width ~ 1.1. Pronotal collar/vertex 0.7–0.9. Prescutellar sulcus with complete median carina plus 3 pairs of distinct but weaker lateral carinae. Mesoscutum posterior border with distinct complete carina. Metanotum with mid-longitudinal carina complete, connecting to a carinate pit posteriorly, bisecting the posterior pit in paratype. Metanotum mid-pit present, delimited by carinae. Mid-longitudinal carina of propodeum complete. Ventral mid-line of mesopleuron set within shallow smooth sulcus; pit at ventral mid-line absent. Notauli weakly indicated anteriorly, indistinctly crenulate. Sternaulus absent. Sculpture of mesosoma mostly granulate. Pronotum rugose laterally, short subventral longitudinal carina present. Mesopleuron mostly rugose. Subalar groove crenulate. Mid-posterior region of mesoscutum rugose, with a short mid-longitudinal carina posteriorly. Mesoscutellar trough entirely costate. Metanotum mostly smooth and weakly crenulate. Propodeum mostly rugose.

***Wings.*** Fore wing: Stigma length/height 3.3–3.5. Vein r/2RS 1.0–1.1. Vein r/RS+Mb 1.2–1.3. Vein 3RSa/2RS 1.8–1.9. Vein 3RSa/2M 0.80–0.85. Vein 3RSa/3RSb 0.41–0.45. Vein 1CUa/1CUb 0.80–0.85. Vein 1CUa/2CUa 1.7. Vein 1cu-a weakly inclivous. Vein 1M weakly curved near middle. Vein RS+Ma sinuate. Vein M+CU virtually straight. Vein 1-1A very weakly sinuate apically. Vein 1a absent. Second submarginal cell trapezoidal. Subbasal cell glabrous, with two parallel rows of short setae subapically, and a narrow patch of setae just below vein 1CUa. Basal cell mostly setose but glabrous region just above vein M+CU. Hind wing: Vein RS Bent at basal 0.3, with vein r present. Marginal cell narrowest at base. Vein M+CU/1M 1.4–1.5. Vein M+CU/r-m 1.4. Vein m-cu present and tubular. Vein m-cu position relative to vein r-m interstitial, or antefurcal. Vein 2-1A absent. Basal cell evenly setose with a small bare spot posteriorly.

***Hind legs.*** Femur length/width 4.8–5.0. Length of tibia/tarsi ~ 1.0. Length of basitarsus/tarsi 2–4 0.65. Sculpture of hind coxa dorsally granulate. Tarsal claws pectinate basally.

***Metasoma.***T1 length/apical width 1.3–1.4. T2 length/apical width ~ 0.8. T3 length/apical width 0.5–0.6. Mid-longitudinal carina extending until basal 0.7 of T3. Metasoma sculpture T1 rugose, T2 and most of T3 striate-rugose, or remainder terga granular-coriaceous. Ovipositor sheath/hind basitarsus ~ 0.4. Apex of ovipositor sheaths truncate; apical point absent.

***Color*** (Fig. 66). Body mottled pale yellow, orange and dark brown. Head pale yellow, clypeus and part of face just above clypeus brown, mandibles pale brown with dark brown teeth. Antenna yellow. Mesosoma mostly pale yellow except propodeum orange; dark brown markings at propleuron and pronotum anteriorly, mesopleuron below subalar groove and ventrally, posterior corners of mesoscutum, and scutellum; metanotum and part of metapleuron pale brown. Metasoma orange, pale yellow ventrally. Wings slightly infuscate, most veins dark brown, costal vein brownish orange, basal veins yellow; stigma mostly dark brown with both tips whitish yellow; tegula brown or dark brown. Legs with trochanter, trochantellus, tibia and tarsi 1–4 whitish yellow; coxae and femora dark brown, but light yellow at base; fifth tarsomeres yellow; exceptions: hind coxa mostly brown, hind tibia with apical 0.25 dark brown.

**Male.** Very similar to female but fifth tarsomeres usually dark brown. Body length 7.1–7.9 mm, fore wing length 5.8–6.3; 49–52 antennomeres.

##### Diagnosis.

*Aleiodes maculosus* can be easily distinguished by its mottled pale yellow, orange and dark brown body colors (Figs 66–68). It is most similar to *A. brevicarina*, but differs in having the fore coxa dark brown, stigma dark brown (Fig. 66), palpi yellow to pale brown (Figs 66, 68), face light yellow with mid-ventral brown spot which extends to clypeus and mandibles (Fig. 68), tegula infuscate (Fig. 67), and antenna entirely yellow (Fig. 66) (the antenna is basally dark brown in *A. brevicarina*). *Aleiodes maculosus* has a complete longitudinal carina on the propodeum, whereas the propodeal carina of *A. brevicarina* is quite short, extending over less than half the length of the propodeum.

##### Distribution.

This species is known only from localities in Brazil.

##### Etymology.

The specific epithet *maculosus* is Latin for dappled or spotted, a reference to the mottled color pattern in this species (Figs 66–68).

#### Aleiodes nigristemmaticum

Taxon classificationAnimaliaHymenopteraBraconidae

(Enderlein, 1920)

205BE352-B441-58BD-AAFD-93989DFBBBA1

Figs 69–72

Rhogas nigristemmaticum Enderlein, 1920: 156.
Aleiodes nigristemmaticum : Marsh & S.R. Shaw, 1998: 400. New combination, lectotype designation, and distribution.

##### Type material.

Lectotype, female. Mexico, Chiapas (PASW). Examined by SRS (see Marsh and S.R. Shaw, 1998).

##### Non-type material examined.

In addition to the specimens studied by Marsh and S.R. Shaw (1998), which were re-examined, the following specimens were studied: 1 female, BOLIVIA: Santa Cruz, Nuflo de Chavez, xi.1963 (CNCI). 30 females, BRAZIL: Encruzilhada, 960m, light culls, xi.1972, M. Alvarenga (CNCI). 1 female, BRAZIL: M. Gerais, Pedra Azul, xi.1972 (CNCI). 15 females and 2 males, BRAZIL: Mato Grosso do Sul [MS], Aquidauana, 20°25'54"S, 55°39'21"W, Malaise trap, 26.x.2011, Lamas & Nihei cols. (DCBU). 1 female, BRAZIL: Ceará [CE], Crato, Chapada do Araripe, 07°13'56"S, 39°26'16.5"W, light trap, 10.ii.2013, A.S. Soares & E.M. Shimbori cols. (DCBU). 1 female, BRAZIL: Bahia [BA], Morro do Chapéu, Pq. Est. Morro do Chapéu, 11°24'44"S, 41°19'55"W, light trap, 19.iv.2013 (DCBU). 6 females and 3 males, BRAZIL: Piauí [PI], Coronel José Dias, PARNA Serra da Capivara, Pedra Furada, 08°50'11"S, 42°32'55"W, light trap, 20.iii.2013, A.S. Soares & E.M. Shimbori cols. (DCBU). 2 males, BRAZIL: Pernambuco [PE], Agrestina, Fazenda Amapá, 11–17.vi.1971, Exp[edition] ABC-MZUSP (MZUSP). 6 females, BRAZIL: Goiás [GO], Cabeceiras (Lagoa Formosa), 24–27.x.1964, Exp[edition] Dep. Zool. (MZUSP). 5 females, BRAZIL: São Paulo [SP], Luiz Antônio, Mogi Guaçu River, light: 1 female, 27.iii.1987, L.A. Joaquim col., 1 female, 18.ii.1988, L.A. Joaquim col., 3 females, 2.iii.1994, A.S. Soares col. (DCBU); 1 female, BRAZIL: São Paulo [SP], Caraguatatuba, 40m, (Res. Flor.), 2.iv.1962, K. Lenko col. (MZUSP). 1 female, COLOMBIA: Vichada PNN, El Tupparo Bosque Sabana, 5°21'N, 67°51'W, 100m, Malaise, 15–19.vii.2000, W. Villalba leg. M511 (IHCB). 1 male, CUBA: Soledad, 25.ii.1925, Geo. Salt (CNCI). 1 male, DOMINICAN REPUBLIC: La Vega Province, Bonao, 05.ix.1997, UV light, hotel courtyard, Baranowski R. (CNCI). 1 female, REP. DOMINICANA: La Cumbre, 600m, L. Masner (CNCI). 1 male, HONDURAS: Comayagua, along road north of Meambar, 13 December 1987, R.D. Cave, col. (UWIM). 1 female, MEXICO: Chiapas, 16°58'N, 91°47'W, 6–9.xi.1978, J. Rawlins (CNCI). 1 female, 2 males, VENEZUELA: Cagua Edo. Aragua, i.1974, light trap (UWIM).

##### Description of non-type specimens.

Body length 5.9–7.5 mm. Fore wing length 4.8–6.3 mm.

***Head.*** In dorsal view eye length/temple 3.0–4.0. Eye height/head width 0.39–0.44. Eye height/minimum distance between eyes 1.0–1.3. OD/POL 2.0–3.3. OD/OOL 2.0–2.8. Frons excavated. Frons lateral carina present. Occipital carina dorsally complete and nearly straight, or weakly bent mid-dorsally. Occiput in dorsal view nearly straight, not indented medially. Occipital carina ventrally meeting hypostomal carina. Mid-longitudinal crest at upper face present. Hypoclypeal depression/face width 0.33–0.36. Malar space/eye height 0.20–0.24. Face height/width 0.7–0.8. Clypeus height/width 0.7–0.8. Clypeus convex, granulate. Sculpture of head shiny granular-coriaceous. Face transversely rugose-striate at dorsal half, or mostly transversely rugose-striate, medially granular-coriaceous below crest.

***Antenna.*** Antennal segments 51–55. Antenna/body length ~ 1.2. Scape/pedicel length 1.8–1.9. Length of first/second flagellomere 1.1–1.2. Fourth flagellomere length/apical width 1.8–2.0. Tip of apical segment of antenna nipple-shaped.

***Mesosoma.*** Length/height 1.7–1.8. Width of mesoscutum/width of head 0.6–0.7. Mesoscutum length/width 1.0–1.2. Pronotal collar/vertex 0.6–0.9. Prescutellar sulcus with complete mid-longitudinal carina plus two or three pairs or lateral carinae more or less defined. Mesoscutum posterior border with distinct complete carina. Metanotum with mid-longitudinal carina present anteriorly. Metanotum mid-pit present, delimited by carinae. Mid-longitudinal carina of propodeum complete or nearly complete, usually irregular posteriorly. Ventral mid-line of mesopleuron set within smooth sulcus; pit at ventral mid-line absent, or weakly indicated. Notauli weakly indicated anteriorly, indistinctly crenulate. Sternaulus absent. Sculpture of mesosoma mostly granulate. Pronotum mostly rugose-costate laterally, short subventral longitudinal carina present. Mesopleuron rugose centrally and anteriorly. Subalar groove sparsely crenulate. Mid-posterior region of mesoscutum rugose, with a short mid-longitudinal carina posteriorly. Mesoscutellar trough entirely costate. Metanotum mostly smooth, with one or two pairs of lateral carinae. Propodeum rugose posteriorly, or mostly rugose.

***Wings.*** Fore wing: Stigma length/height 3.1–3.4. Vein r/2RS 0.9–1.1. Vein r/RS+Mb 1.2–1.5. Vein 3RSa/2RS 1.3–1.6. Vein 3RSa/2M 0.76–0.85. Vein 3RSa/3RSb 0.36–0.44. Vein 1CUa/1CUb 0.8–0.9. Vein 1CUa/2CUa 1.4–1.8. Vein 1cu-a weakly inclivous, or nearly vertical. Vein 1M weakly curved basally. Vein RS+Ma distinctly curved. Vein M+CU virtually straight. Vein 1-1A very weakly sinuate apically. Vein 1a absent. Second submarginal cell trapezoidal. Subbasal cell glabrous, with two parallel rows of short setae subapically, and a narrow patch of setae just below vein 1CUa, very few scattered setae may be present medially. Basal cell mostly evenly setose, sparsely setose posteriorly, with a bare spot posteriorly. Hind wing: Vein RS bent at basal 0.3, with vein r present. Marginal cell narrowest at base. Vein M+CU/1M 1.3–1.6. Vein M+CU/r-m 1.2–1.7. Vein m-cu present, spectral. Vein m-cu position relative to vein r-m antefurcal, or nearly interstitial. Vein 2-1A absent. Basal cell evenly, rather sparsely setose, posteriorly with small bare area.

***Hind legs.*** Femur length/width 5.3–5.6. Length of tibia/tarsi 0.9–1.0. Length of basitarsus/tarsi 2–4 0.7–0.8. Sculpture of hind coxa dorsally granulate. Tarsal claws not pectinate.

***Metasoma.***T1 length/apical width 1.1–1.3. T2 length/apical width 0.8–0.9. T3 length/apical width 0.5–0.7. Mid-longitudinal carina extending until basal 0.7 of T3. Metasoma sculpture T1, T2 and basal 0.7 of T3 rugose-costate, remainder terga granular-coriaceous. Ovipositor sheath/hind basitarsus 0.3–0.5. Apex of ovipositor sheaths truncate; apical point absent.

**Figures 69–72.**
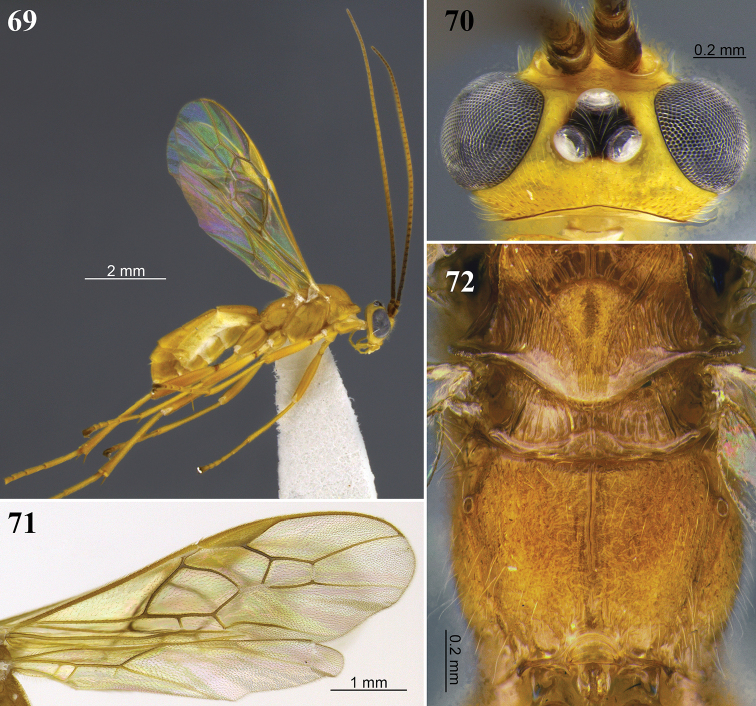
*Aleiodes nigristemmaticum* (Enderlein). **69** Lateral habitus **70** head, dorsal view **71** wings **72** mesosoma and propodeum, dorsal view.

***Color*** (Figs 69–72). Body entirely pale yellow to brownish yellow (variation among specimens); antenna varying from mostly yellow to entirely dark brown, usually dark brown basally gradually lighter to apically pale yellow, but commonly darker at apex (lighter only at middle); scape yellow or honey yellow with brown lateral stripe, pedicel brown or dark brown. Wings hyaline, veins and stigma yellow, but vein r and 1M darker, stigma rarely with a nearly central infuscate dot. Legs mostly brownish yellow, usually trochanter and trochantellus slightly lighter and femur slightly darker than remainder legs; all fifth tarsomeres mostly brown, darker than remainder tarsi.

**Male.** Essentially as in female. Body length 5.5–7.0 mm; fore wing length 4.3–5.4 mm; antenna with 48–50 segments.

##### Diagnosis.

Traditionally this very common and widespread species has been recognized by the predominantly yellow body color, yellow stigma, and sharply contrasting black ocellar triangle (hence the name *nigristemmaticum*) (Figs 69, 70). The following characters are also useful for distinguishing this species from others treated in this paper: fore wing vein r approximately as long as 2RS and shorter than 3RSa, second submarginal cell trapezoidal (Fig. 71); antenna brown basally, gradually lightening to light brown apically, scape and pedicel mostly honey yellow with brown lateral stripe (Fig. 69); fourth flagellomere 1.8–1.9 × longer than wide; occiput not receding mid-dorsally (Fig. 70); fifth tarsomeres darker than remainder of tarsi in all legs (Fig. 69).

##### Biology.

Parasitoid of *Mocis latipes* (Guenée.) and *Mocis* spp (Erebidae, Erebinae), mostly feeding on grasses (Poaceae), including several crops. Additional details regarding biological information are given by Marsh and S.R. Shaw (1998).

##### Distribution.

USA (Florida and Mississippi); Mexico; Honduras; Cuba; Costa Rica; Venezuela; Peru, Brazil, Bolivia, Ecuador, Dominican Republic, Panama, Puerto Rico, Honduras, Colombia, and Suriname. Its widespread distribution may be a reflection of the distribution and pest status of the host, which feeds on grasses, as well as crops such as corn and rice. This species is recorded from Florida to Southern Brazil.

#### Aleiodes ovatus

Taxon classificationAnimaliaHymenopteraBraconidae

Shimbori & Shaw sp. nov.

F91B5843-7CE6-50AB-A315-C04B06C33AC0

http://zoobank.org/5E0F8CE2-4982-4F19-BAF5-FE78706B0537

Figs 73–75

##### Type material.

Holotype, female (UEFS #33424) “Brasil, BA, Seabra, 12°27'S, 41°44'W 15.XI.2007 Leg. Alvim, E.”

Paratypes. 2 females, 1 male (UEFS #s:33396, 33406, 33404), same as holotype; 1 male (DCBU #20792) “FAZ. CANCHIM SÃO CARLOS – SP luz 11.II.1983 A.S. Soares col.”

##### Description.

***Body*** length 6.8–7.0 mm. Fore wing length 5.9–6.3 mm.

***Head.*** In dorsal view eye length/temple 4.2–4.5. Eye height/head width 0.43–0.45. Eye height/minimum distance between eyes 1.4–1.5. OD/POL 2.9–3.1. Ocelli exceptionally large, OD/OOL 3.7–4.6 (Fig. 74). Frons excavated. Frons lateral carina present. Occipital carina dorsally complete and nearly straight. Occiput in dorsal view nearly straight, not indented medially. Occipital carina ventrally meeting hypostomal carina. Mid-longitudinal crest at upper face present. Hypoclypeal depression/face width 0.35. Malar space/eye height 0.16–0.17. Face height/width 0.83–0.86. Clypeus height/width 0.6–0.7. Clypeus convex, granulate. Sculpture of head mostly shiny granulate, vertex granulate-rugose, frons shiny granular-coriaceous. Face mostly transversely rugose-striate, granulate medially.

***Antenna.*** Antennal segments 54. Antenna/body length 1.3. Scape/pedicel length 2.0. Length of first/second flagellomere 1.2–1.3. Fourth flagellomere length/apical width 1.7. Tip of apical flagellomere pointed.

***Mesosoma.*** Length/height ~ 1.7. Width of mesoscutum/width of head 0.67–0.72. Mesoscutum length/width ~ 1.1. Pronotal collar/vertex 0.7–0.8. Prescutellar sulcus with 5–7 distinct carinae. Mesoscutum posterior border with distinct complete carina. Metanotum with mid-longitudinal carina present anteriorly, with carinate pit mid-posteriorly. Metanotum mid-pit present, delimited by carinae. Mid-longitudinal carina of propodeum present at basal 0.7, absent posteriorly. Ventral mid-line of mesopleuron set within shallow smooth sulcus; pit at ventral mid-line weakly indicated. Notauli present anteriorly, shallowly and weakly crenulate. Sternaulus absent. Sculpture of mesosoma mostly granulate. Pronotum rugose laterally, short subventral longitudinal carina present. Mesopleuron mostly rugose. Subalar groove crenulate. Mid-posterior region of mesoscutum rugose with long and irregular mid-longitudinal carina. Mesoscutellar trough entirely costate. Metanotum mostly smooth and weakly crenulate. Propodeum mostly rugose.

***Wings*** (Fig. 75). Fore wing: Stigma length/height 3.3. Vein r/2RS 0.9–1.0. Vein r/RS+Mb 1.2–1.3. Vein 3RSa/2RS 1.6–1.7. Vein 3RSa/2M 0.85–0.89. Vein 3RSa/3RSb 0.41–0.45. Vein 1CUa/1CUb 0.9–1.0. Vein 1CUa/2CUa 1.9–2.0. Vein 1cu-a weakly inclivous. Vein 1M weakly curved basally. Vein RS+Ma sinuate. Vein M+CU virtually straight. Vein 1-1A weakly sinuate at apex. Vein 1a absent. Second submarginal cell trapezoidal. Subbasal cell glabrous, with two parallel rows of short setae subapically, and a narrow patch of setae just below vein 1CUa. Basal cell mostly evenly setose, sparsely setose posteriorly. Hind wing: Vein RS Bent at basal 0.3, with vein r present. Marginal cell narrowest at base. Vein M+CU/1M 1.6–1.7. Vein M+CU/r-m 1.3–1.4. Vein m-cu present and pigmented, although not tubular. Vein m-cu position relative to vein r-m postfurcal, or interstitial. Vein 2-1A absent. Basal cell evenly, rather sparsely setose, posteriorly with small bare area.

***Hind legs.*** Femur length/width 4.8–5.0. Length of tibia/tarsi 0.9–1.0. Length of basitarsus/tarsi 2–4 ~ 0.7. Sculpture of hind coxa dorsally mostly shiny granular-coriaceous, finely striate apically. Tarsal claws pectinate basally.

***Metasoma.***T1 length/apical width ~ 1.3. T2 length/apical width 0.8–1.0. T3 length/apical width 0.6–0.7. Mid-longitudinal carina extending until basal ~ 0.7 of T3. Metasoma sculpture T1, T2, and basal ~ 0.7 of T3 rugose-costate, remainder metasoma smooth. Ovipositor sheath/hind basitarsus 0.25–0.40. Apex of ovipositor sheaths truncate and narrow; apical point absent.

**Figures 73–75.**
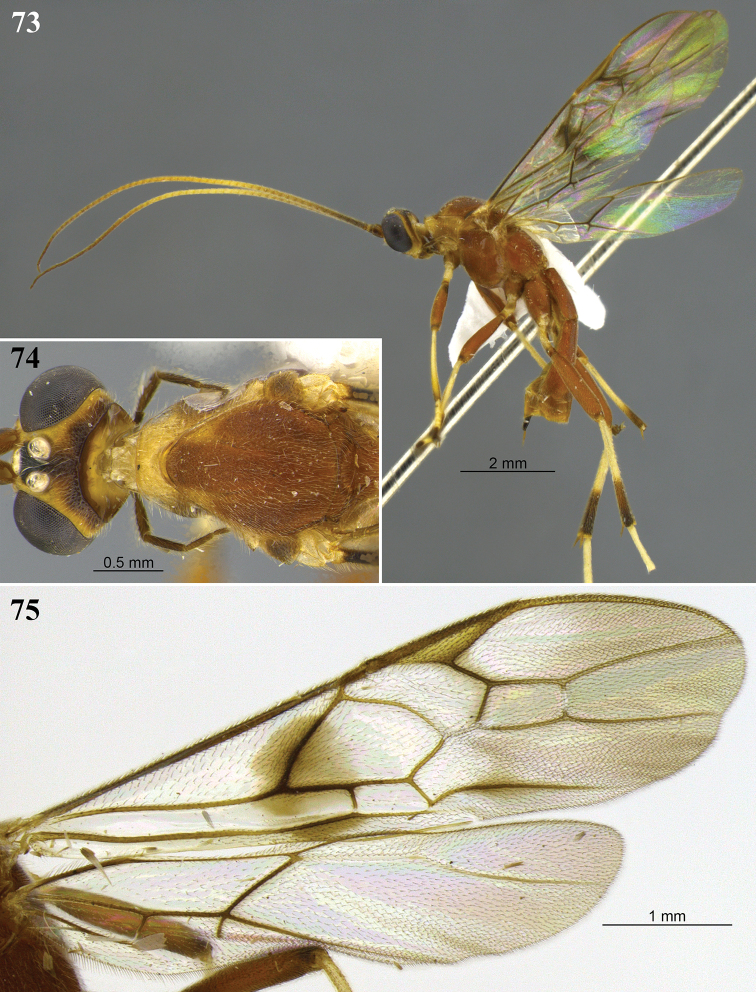
*Aleiodes ovatus* sp. nov. **73** lateral habitus **74** head and anterior mesosoma, dorsal view **75** wings.

***Color*** (Figs 73–75). Brownish orange. Head light yellow with a dark brown spot dorsally from stemmaticum and along occipital carina on vertex (Fig. 74), and a brown spot on face, covering clypeus and part of face on each side of the clypeus; palpi dark brown. Antenna brown basally, lightening to light yellow medially, then darkening to brown apex. Pronotum mostly pale yellow except laterally brownish orange; anterior corner of mesopleuron pale yellow; propleuron mostly brown with light yellow borders. Legs with trochanter, trochantellus, most of tibia, and tarsomeres 1–4 whitish yellow; all fifth tarsi dark brown; all tibiae dark brown apically, dark region smaller in frontal and mid legs; hind trochanter and trochantellus with brown lateral spots. Wings weakly tinged brown, veins and stigma brown; fore wing with an infuscate oval spot around junction of veins 1M and 1CU (Fig. 75). Ovipositor sheaths black.

**Male.** Essentially as in female. Body length 6.8–7.0 mm, fore wing length 5.5 mm.

##### Diagnosis.

*Aleiodes ovatus* is similar to *A. brevicarina* and *A. maculosus* in having a whitish yellow hind tibia with dark brown apex (as in Figs 29, 66, 73). *Aleiodes ovatus* can be distinguished from both species by the oval infuscate spot on fore wing (Fig. 75). In the other two species dark coloration is present only along the veins (Figs 32, 66) and does not form a large spot. Also distinctive for *A. ovatus* is the mostly light yellow head with a dark brown vertex (except for orbits) (Fig. 74), whereas in *A. brevicarina* and *A. maculosus* the vertex does not have any dark brown markings (Figs 30, 67).

##### Distribution.

This species is known only from localities in Brazil.

##### Etymology.

The name *ovatus* is Latin for oval or egg-shaped, being a reference to the distinctive oval marking on the fore wing in this species (Fig. 77).

#### Aleiodes santarosensis

Taxon classificationAnimaliaHymenopteraBraconidae

Shaw & Shimbori sp. nov.

CDBEF121-A6EA-50A9-8C91-B340F12D616F

http://zoobank.org/0DB7477F-54D6-46EB-885E-410E5826A6F2

Figs 76–78

##### Type material.

Holotype, female (UWIM) “Costa Rica, Guanacaste, Pr. Guan. Conservation Area Santa Rosa hdq. 200m lighttrap, 7-VII-1997 L. J. van der Ent.”

Paratypes. 4 females, 12 males (UWIM), same as holotype; 2 females, 1 male (UWIM), same data except “6.VII.1997”; 1 female, 2 males (UWIM), same data except “27–30.VI.1997”; 1 male (UWIM), same data, except “… at Dorms UV Light, 3 June 1995 Dadelahi, Prie, Zitani”; 1 female (INBIO) “3km NO de Nacaome, 100m, P. N. Barra Honda, Prov. Guan. COSTA RICA, 3 a 30 mayo 1993 M. Reyes L-N 239000, 386000.”

##### Description.

Body length 6.8–8.3 mm. Fore wing length 6.0–6.9 mm.

***Head*** (Fig. 77). In dorsal view eye length/temple 4.9–6.3. Eye height/head width 0.42–0.44. Eye height/minimum distance between eyes 1.3–1.4. OD/POL 3.8–5.2. OD/OOL 2.9–3.8. Frons excavated. Frons lateral carina absent. Occipital carina dorsally complete and nearly straight. Occiput in dorsal view nearly straight, not indented medially. Occipital carina ventrally meeting hypostomal carina. Mid-longitudinal crest at upper face present. Hypoclypeal depression/face width 0.35. Malar space/eye height 0.16. Face height/width 0.7. Clypeus height/width 0.75. Clypeus convex, granulate. Sculpture of head shiny granular-coriaceous. Face transversely rugose-striate around median crest.

***Antenna.*** Antennal segments 51–54. Antenna/body length 1.1–1.2. Scape/pedicel length 2.0–2.1. Length of first/second flagellomere 1.1–1.2. Fourth flagellomere length/apical width 1.4–1.6. Tip of apical flagellomere pointed.

***Mesosoma.*** Length/height ~ 1.6. Width of mesoscutum/width of head 0.7. Mesoscutum length/width 1.1–1.2. Pronotal collar/vertex 1.0–1.1. Prescutellar sulcus with 7–9 distinct carinae. Mesoscutum posterior border with distinct complete carina. Metanotum with mid-longitudinal carina complete, connecting to a carinate pit posteriorly. Metanotum mid-pit present, delimited by carinae. Mid-longitudinal carina of propodeum present at basal ~ 0.7, absent posteriorly. Ventral mid-line of mesopleuron set within shallow smooth sulcus; pit at ventral mid-line weakly indicated. Notauli weakly indicated anteriorly, indistinctly crenulate. Sternaulus weakly indicated anteriorly, rugose. Sculpture of mesosoma mostly granulate. Pronotum granulate ventrally, pronotal groove mostly crenulate, short subventral longitudinal carina present. Mesopleuron rugose below subalar groove. Subalar groove sparsely crenulate. Mid-posterior region of mesoscutum rugose, with a short mid-longitudinal carina posteriorly, with irregularly carinate notauli. Mesoscutellar trough entirely costate. Metanotum mostly smooth, with one or two pairs of lateral carinae. Propodeum mostly rugose.

***Wings.*** Fore wing: Stigma length/height 3.3–3.4. Vein r/2RS 1.25–1.45. Vein r/RS+Mb 1.2–1.6. Vein 3RSa/2RS ~ 1.7. Vein 3RSa/2M 0.85–0.89. Vein 3RSa/3RSb ~ 0.4. Vein 1CUa/1CUb 0.8–0.9. Vein 1CUa/2CUa 1.8–1.9. Vein 1cu-a inclivous. Vein 1M weakly curved basally, or weakly, evenly curved. Vein RS+Ma distinctly curved. Vein M+CU virtually straight. Vein 1-1A weakly sinuate at apex. Vein 1a absent. Second submarginal cell trapezoidal. Subbasal cell glabrous, with two parallel rows of short setae subapically. Basal cell mostly glabrous, setose below costal vein and around dark spot near vein 1M. Hind wing: Vein RS bent at basal 0.3, with vein r present. Marginal cell narrowest at base. Vein M+CU/1M 1.6–1.8. Vein M+CU/r-m 1.4. Vein m-cu present, spectral, or partly tubular. Vein m-cu position relative to vein r-m interstitial, or antefurcal. Vein 2-1A absent. Basal cell sparsely setose, bare posteriorly.

***Hind legs.*** Femur length/width 4.6. Length of tibia/tarsi ~ 0.9. Length of basitarsus/tarsi 2–4 ~ 0.7. Sculpture of hind coxa dorsally granulate. Tarsal claws not pectinate. Metasoma. T1 length/apical width ~ 1.1. T2 length/apical width ~ 0.8. T3 length/apical width 0.5–0.6. Mid-longitudinal carina extending until basal ~ 0.7 of T3. Metasoma sculpture T1 rugose, T2 and most of T3 striate-rugose, or sculpture weaker at T3, or remainder terga granular-coriaceous. Ovipositor sheath/hind basitarsus 0.42–0.54. Apex of ovipositor sheaths truncate; apical point absent.

***Color.*** Brownish yellow or brownish orange. Antenna entirely brownish yellow. Legs mostly brownish orange, tibia and tarsi whitish yellow except hind tibia gradually darkening from whitish yellow basally to brownish orange apically. Wings weakly tinged yellow, vein brownish yellow except fore wing veins 1M, 1CU, apex of 1-1A, 2CUb medially, r, and veins of second submarginal cell brown or dark brown. Ovipositor sheaths dark brown.

**Male.** Essentially as in female. Body length 6.7–7.8 mm; fore wing length 5.3–6.0 mm; antenna with 49–52 segments.

##### Diagnosis.

**Figures 76–78.**
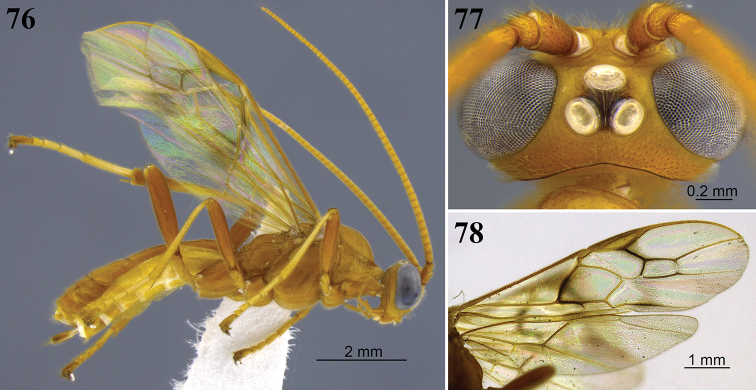
*Aleiodes santarosensis* sp. nov. **76** lateral habitus **77** head, dorsal view **78** wings.

*Aleiodes santarosensis* is a mainly brownish yellow species, with the whole antenna brownish yellow and whitish yellow tibia and tarsi 1–4 (Fig. 76). This is the only species in this study with tibia basally and tarsi 1–4 whitish yellow but without dark brown regions on apex of hind femur and tibia (Fig. 76). It is similar to *A. brevicarina*, from which is can be distinguished also by the large glabrous regions on the discal and basal cells of fore wing (Fig. 78). In contrast, these cells are mostly evenly setose in *A. brevicarina* (Fig. 32).

##### Distribution.

Known only from localities in northwest Costa Rica.

##### Etymology.

The name *santarosensis* refers to Santa Rosa National Park in Guanacaste Province of northwest Costa Rica, the type-locality of this species.

#### Aleiodes taurus

Taxon classificationAnimaliaHymenopteraBraconidae

Shimbori & Penteado-Dias sp. nov.

44F22E98-800C-52F4-8218-8470B50EA2D3

http://zoobank.org/EE216129-501C-4DFA-B1AD-DBD764182066

Figs 79–84

##### Type material.

Holotype, female (DCBU #21814) “FAZ. CANCHIM SÃO CARLOS – SP luz, MATA, 19.X.1982 A.S. Soares col.”

##### Description.

Body length 7.7 mm. Fore wing length 6.4 mm.

***Head*** (Fig. 80). In dorsal view eye length/temple 3.4–3.9. Eye height/head width 0.4. Eye height/minimum distance between eyes 1.1–1.2. OD/POL 2.2–2.5. OD/OOL 2.4–2.8. Frons excavated. Frons lateral carina present in addition to W-shaped carina. Occipital carina dorsally complete and nearly straight. Occiput in dorsal view nearly straight, not indented medially. Occipital carina ventrally meeting hypostomal carina. Mid-longitudinal crest at upper face present. Hypoclypeal depression/face width 0.37. Malar space/eye height 0.19. Face height/width 0.75. Clypeus height/width ~ 0.6. Clypeus convex, granulate. Sculpture of head vertex coarsely granulate, frons rugose. Face transversely rugose-striate at dorsal half, granulate medially.

***Antenna.*** Antennal segments 30+ (antenna broken). Antenna/body length? (antenna broken). Scape/pedicel length 2.6. Length of first/second flagellomere 1.2. Fourth flagellomere length/apical width 1.7. Tip of apical segment of antenna missing.

***Mesosoma.*** Length/height ~ 1.6. Width of mesoscutum/width of head 0.74. Mesoscutum length/width ~ 1.0. Pronotal collar/vertex 0.8. Prescutellar sulcus with entirely costate, lateral carina oblique and nearly reaching anterior border. Mesoscutum posterior border with distinct complete carina. Metanotum with complete mid-longitudinal carina, carinate posterior pit bisected by carina. Metanotum mid-pit present, delimited by carinae. Mid-longitudinal carina of propodeum present and basal 0.5 or less. Ventral mid-line of mesopleuron set within shallow smooth sulcus; pit at ventral mid-line present, shallow. Notauli present anteriorly, shallowly and weakly crenulate. Sternaulus weakly indicated anteriorly, rugose. Sculpture of mesosoma mostly granulate. Pronotum rugose laterally, pronotal groove crenulate laterally, with two parallel subventral carinae. Mesopleuron rugose below subalar groove. Subalar groove sparsely crenulate. Mid-posterior region of mesoscutum rugose, with a short mid-longitudinal carina posteriorly. Mesoscutellar trough entirely costate. Metanotum costate. Propodeum mostly rugose.

***Wings.*** Fore wing: Stigma length/height 3.4. Vein r/2RS 1.5. Vein r/RS+Mb 1.4. Vein 3RSa/2RS 2.1. Vein 3RSa/2M 0.9. Vein 3RSa/3RSb 0.44. Vein 1CUa/1CUb 1.0. Vein 1CUa/2CUa 1.9. Vein 1cu-a nearly vertical. Vein 1M weakly curved basally. Vein RS+Ma distinctly curved. Vein M+CU virtually straight. Vein 1-1A nearly straight. Vein 1a absent. Second submarginal cell rectangular, slightly widening toward apex. Subbasal cell glabrous, with two parallel rows of short setae subapically, and a narrow patch of setae just below vein 1CUa. Basal cell with more or less large glabrous region posteriorly, sometimes with sparse setae; costal and apical regions evenly setose. Hind wing: Vein RS bent at basal 0.3, with vein r present. Marginal cell narrowest at base. Vein M+CU/1M 1.5. Vein M+CU/r-m 1.5. Vein m-cu present, spectral. Vein m-cu position relative to vein r-m just antefurcal. Vein 2-1A absent. Basal cell sparsely setose, bare posteriorly.

***Hind legs.*** Femur length/width 5.2. Length of tibia/tarsi ~ 0.9. Length of basitarsus/tarsi 2–4 0.65. Sculpture of hind coxa dorsally mostly shiny granular-coriaceous, finely striate apically. Tarsal claws not pectinate.

***Metasoma.***T1 length/apical width ~ 1.2. T2 length/apical width 0.7. T3 length/apical width 0.6. Mid-longitudinal carina extending until basal 0.7 of T3. Metasoma sculpture: T1, T2 and basal 0.7 of T3 rugose-costate, sculpture weaker at T3, remainder metasoma smooth. Ovipositor sheath/hind basitarsus 0.55. Apex of ovipositor sheaths roughly rounded; apical point present, distinct.

**Figures 79–84.**
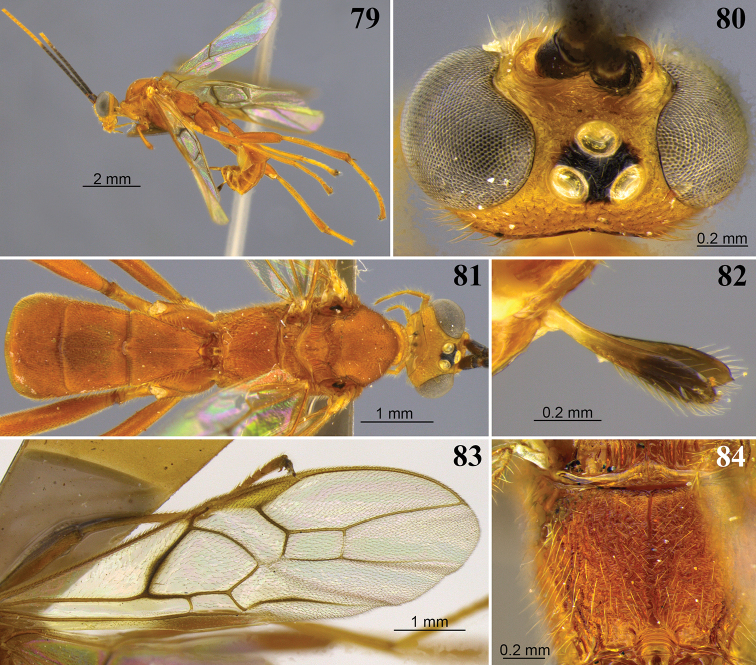
*Aleiodes taurus* sp. nov. **79** lateral habitus **80** head, dorsal view **81** dorsal habitus **82** ovipositor sheaths showing rounded ends with apical point **83** wings **84** propodeum, dorsal view.

***Color*** (Figs 79–84). Brownish orange. Head and pronotal collar yellow, stemmaticum black. Antenna with basal 12 or 13 flagellomeres black, apical segments brownish orange; pedicel black; scape dark brown to black, ventrally brownish orange. Base of tibiae and tarsi 1–4 slightly lighter than remainder legs. Wings subhyaline; stigma and most veins orange to yellow; vein 1M almost entirely dark brown, veins 1CUa, r, 2RS, and apex of 2CUb brown; infuscate areas around base of vein 1M and below apex of vein 1-1A. Ovipositor sheaths dark brown.

**Male.** Unknown.

##### Diagnosis.

*Aleiodes taurus* is most similar to *A. gonodontivorus*. The main distinguishing characters are the differently shaped second submarginal cell, long and widening apically, with vein 3RSa 2.1 × longer than vein 2RS (Fig. 83), and the propodeum with very short longitudinal carina (Fig. 84) in *A. taurus*. In *A. gonodontivorus* the vein 3RSa is at most 1.7 × longer than 2RS (Fig. 44), and the longitudinal carina of propodeum is nearly complete.

##### Distribution.

This species is known only from the type-locality in Brazil.

##### Etymology.

The name is from the Latin word *taurus* meaning bull, being a reference to the collecting locality. The holotype was collected in a forest fragment at the research station of the Brazilian Agricultural Research Corporation - EMBRAPA, formerly a farm named Fazenda Canchim, in which a breed of beef cattle was developed, the Canchim, between 1940 and 1970. This area now comprises one of the largest remaining fragments of forest in the municipality of São Carlos.

## Supplementary Material

XML Treatment for Aleiodes andinus

XML Treatment for Aleiodes angustus

XML Treatment for Aleiodes asenjoi

XML Treatment for Aleiodes bahiensis

XML Treatment for Aleiodes bakeri

XML Treatment for Aleiodes barrosi

XML Treatment for Aleiodes brevicarina

XML Treatment for Aleiodes coariensis

XML Treatment for Aleiodes goiasensis

XML Treatment for Aleiodes gonodontivorus

XML Treatment for Aleiodes hyalinus

XML Treatment for Aleiodes inga

XML Treatment for Aleiodes joaquimi

XML Treatment for Aleiodes lidiae

XML Treatment for Aleiodes mabelae

XML Treatment for Aleiodes maculosus

XML Treatment for Aleiodes nigristemmaticum

XML Treatment for Aleiodes ovatus

XML Treatment for Aleiodes santarosensis

XML Treatment for Aleiodes taurus
